# Supplementary material for: Comparative transcriptomics in serial organs uncovers early and pan-organ developmental changes associated with organ-specific morphological adaptation
Source: Nat Commun. 2025 Jan 17;16:768. doi: 10.1038/s41467-025-55826-w (PMC11742040; doi:10.1038/s41467-025-55826-w)
Supplement: Supplementary file 1 — Supplementary Information [file 41467_2025_55826_MOESM1_ESM.pdf]

SUPPLEMENTARY INFORMATION

Comparative transcriptomics in serial organs uncovers early and pan-organ developmental changes associated with organ-specific evolutionary novelty

Tables

Supplementary Table 1: primers and sequences used to synthetize probes

Probe	size	Primers for RT-PCR/synthetized sequence
musWif1	641bp	Fwd ATCCTACCTTGCCTGCTCCT<br>Rev<br>CAGAAGCCAGGAGTGACACA
musDkk1	410bp	Fwd TGGCCGTGTTTACAATGATG<br>Rev AAAATGGCTGTGGTCAGAGG
mesocricetusBMP4	585bp	CTGGTAACCGAATGCTGATGGTCGTTTTATTATGCCAAGTCCTGCTAGGAGGCGCGAGC<br>C<br>ATGCTAGTTTGATACCTGAGACCGGGAAGAAAAAAGTCGCCGAGATTCAGGGCCACGC<br>GG<br>GAGGACGCCGCTCAGGGCAGAGCCATGAGCTCCTGCGGGATTTTGAGGCGACACTTCT<br>GC<br>AGATGTTTGGGCTGCGCCGCCGTCCGCAGCCAAGCAAGAGCGCCGTCATTCCGGATTAC<br>A<br>TGAGGGATCTTTACCGGCTCCAGTCTGGGGAGGAGGAAGAGGAAGAGCAGAGCCAGG<br>GAA<br>TGGGGCTGGAGTACCCCGAGCGTCCAGCCAGCCGGGCCAACACTGTGAGGAGTTTCCA<br>TC<br>ACGAAGAACATCTGGAGAACATCCCAGGGACCAGTGAGAACTCCGCCTTTCGTTTCCTT<br>T<br>TCAACCTCAGCAGCATCCCAGAGAATGAGGTGATCTCCTCTGCGGAGCTCCGCCTGTTT<br>C<br>GGGAGCAGGTGGACCAGGGCCCCGACTGGGAGCGGGGCTTCCACCGGATCAACATTT<br>ATG<br>AAGTTATGAAGCCCCCAGCAGAAATGGTGCCTCGGCACCTCATCA
musBMP4	585bp	Fwd: CTGGTAACCGAATGCTGATGGTC<br>Rev:TGATGAGGTGTCCAGGAACCATT

Supplementary Table 2 : quantification of mesenchyme volume on 3D reconstructed tooth germs

Sample Name	Species	age	embryo weight	tooth	M1 epithelium volume (um3)	M1 total volume (um3)	% epithelium	% mesenchyme
12-47-25	mouse	15	403-405mg	Lower	2,33E+07	6,93E+07	33,60 %	66,40%
12-47-25	mouse	15	403-405mg	Upper	1,80E+07	7,38E+07	24,00 %	76,00%
16-45-19	mouse	15,5	439mg	Upper	2,72E+07	1,02E+08	27,00 %	73,00%
17_02_22	mouse	15,5	439mg	Lower	2,83E+07	6,54E+07	43,00 %	57,00%
15-20-48	mouse	15,5	439mg	Lower	2,65E+07	7,87E+07	33,60 %	66,40%
15-45-51	mouse	15,5	439mg	Upper	2,67E+07	9,42E+07	28,00 %	72,00%
16-17-15	hamster	12,5	431mg	Lower	1,91E+07	7,45E+07	25,00 %	75,00%
16-43-07	hamster	12,5	431mg	Upper	2,64E+07	1,13E+08	23,00 %	77,00%
12-43-17	hamster	13,25	541-547mg	Lower	5,17E+07	1,72E+08	30,00 %	70,00%
16-54-22	hamster	13,25	541-547mg	Lower	4,91E+07	1,60E+08	30,00 %	70,00%
15-00-34	hamster	13,25	541-547mg	Upper	4,75E+07	1,83E+08	26,00 %	74,00%
15-00-34	hamster	13,25	541-547mg	Upper	4,36E+07	1,58E+08	28,00 %	72,00%

Sample Name	Species	age	embryo weight	tooth	M1 epithelium volume (um3)	M1 total volume (um3)	% epitheli um	% mesenchyme
12-47-25	mouse	15	403-405mg	Lower	2,33E+07	6,93E+07	33,60 %	66,40%
12-47-25	mouse	15	403-405mg	Upper	1,80E+07	7,38E+07	24,00 %	76,00%
16-45-19	mouse	15,5	439mg	Upper	2,72E+07	1,02E+08	27,00 %	73,00%
17_02_22	mouse	15,5	439mg	Lower	2,83E+07	6,54E+07	43,00 %	57,00%
15-20-48	mouse	15,5	439mg	Lower	2,65E+07	7,87E+07	33,60 %	66,40%
15-45-51	mouse	15,5	439mg	Upper	2,67E+07	9,42E+07	28,00 %	72,00%
16-32-40	hamster	13,25	541-547mg	Upper	4,41E+07	1,79E+08	24,00 %	76,00%
12_04_30	hamster	13	548mg	Lower	4,55E+07	1,31E+08	34,00 %	66,00%
16-27-27	hamster	13	548mg	Upper	2,67E+07	8,56E+07	31,00 %	69,00%

Supplementary figures

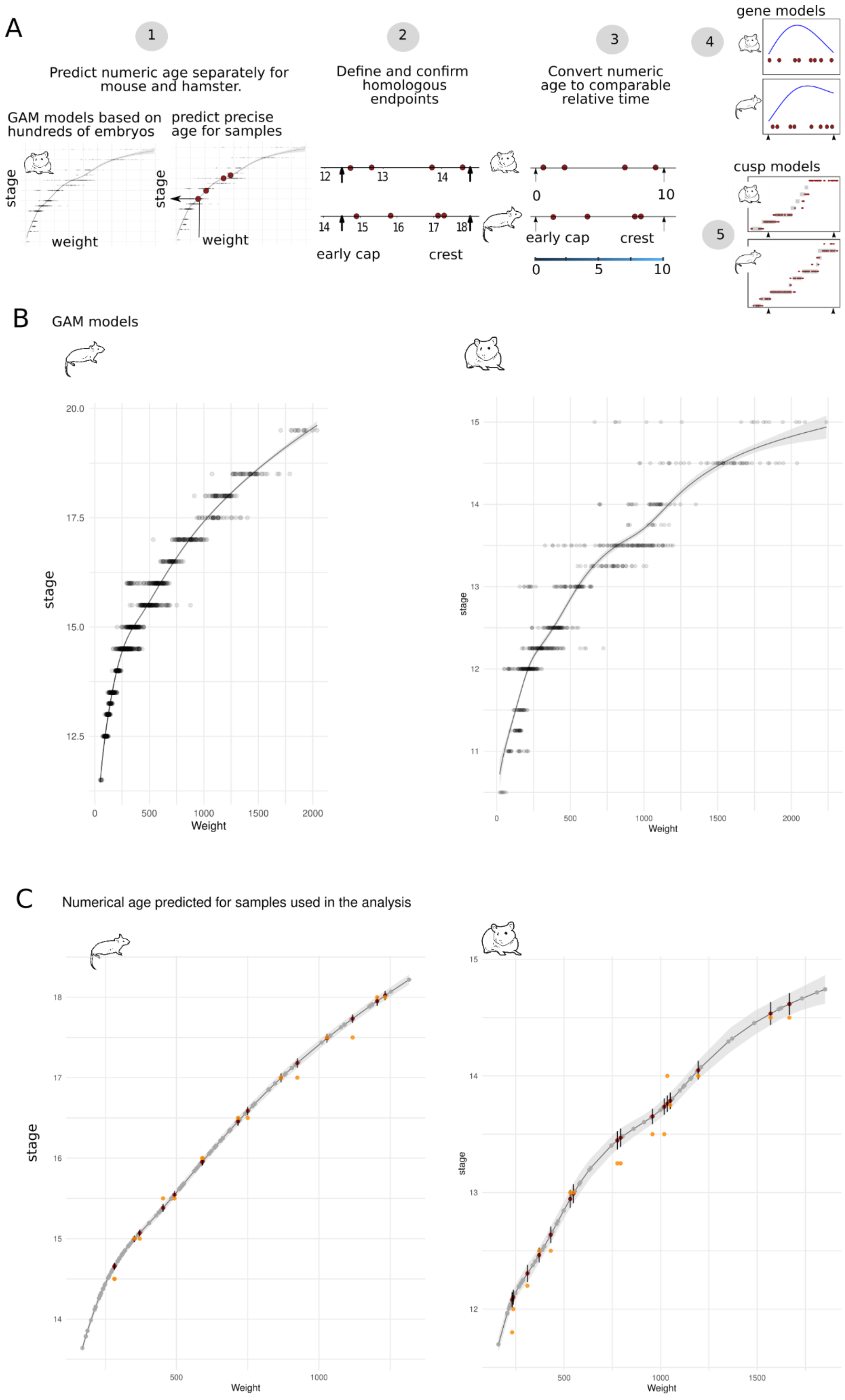
A
1
Predict numeric age separately for mouse and hamster.
GAM models based on hundreds of embryos
predict precise age for samples
stage
weight
2
Define and confirm homologous endpoints
12
13
14
15
16
17
18
early cap
crest
3
Convert numeric age to comparable relative time
0
10
early cap
crest
0
5
10
4
gene models
5
cusp models
B
GAM models
20.0
17.5
15.0
12.5
stage
0
500
1000
1500
2000
Weight
15
14
13
12
11
stage
0
500
1000
1500
2000
Weight
C
Numerical age predicted for samples used in the analysis
18
17
16
15
14
stage
500
1000
Weight
15
14
13
12
stage
500
1000
1500
Weight

Supplementary Figure **1 | Comparing dynamics of morphogenesis and gene expression across teeth and species.**

To compare the gene expression dynamics and cusp patterning we choose to work on fine-scale dynamics rather than comparing samples taken at supposedly homologous stages.

A: We estimate embryos' developmental age from their body weight through a model established by using hundreds of embryos in each species. Numeric age is predicted separately for mouse and hamster (1). Homologous start and end points are milestones defined based on morphology and confirmed by RNA-seq (early cap stage and crest formation, Figures 1&2) (2). Numeric ages are rescaled to comparable relative time of development, from 0 to 10 in each species (3). Relative times are estimated for the 64 RNA-seq samples used to build gene temporal profiles using polynomials (4). Relative times are also estimated for a series of fixed embryos that were hybridised against a *Fgf4* probe to reveal PEK and SEKs. The relative duration of each stage was modelled with continuous Markov processes which assume that very few embryos should be sampled at very transient stages, while many at longer stages.

B: Relationship between embryonic stage (days post coitum, dpc) and weight (mg) was fitted in each species by a GAM model on boxCox transformed values, based on 1047 mouse and 636 hamster embryos respectively.

C: Predicted developmental ages are shown for the samples used to model cusp patterning (grey points) and for the samples used in RNA-seq analysis (whole tooth germs, dark red points). Confidence intervals (95% percentiles) are indicated for each prediction. Days post coitum are indicated in orange for samples used in RNA-seq analysis, for comparison.

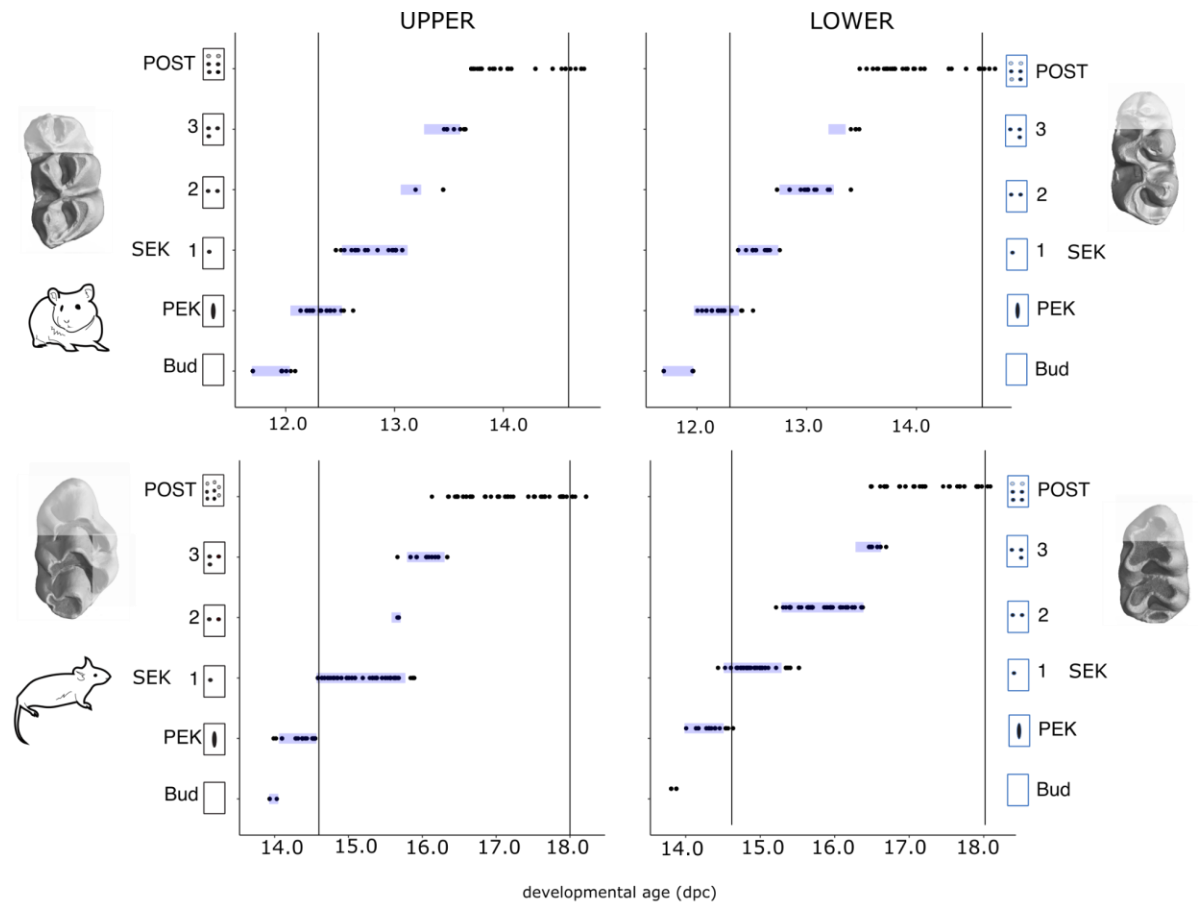

Supplementary Figure 2: **Dynamics and pattern of lingual and posterior cusp addition during early development of lower and upper molars in mouse and hamster.**

To focus on conserved differences in early bucco-lingual/posterior development of lower *versus* upper molar we modelled the patterning of the same four posterior cusps, discarding information about anterior cusps and supplementary lingual cusps. Each panel represents a series of *Fgf4*-hybridised samples (black dots) with their developmental age and signalling centre stage. Stage duration was modelled using markov processes with three different rates (slow, medium and rapid corresponding to long, medium and short stages). Stage durations are represented in blue, and centred on for each stage on developmental time with maximum probability. Developmental age was estimated through a relationship between embryonic weight and age post coitum (dpc). The mouse upper molar shows a shorter PEK stage, longer 1-SEK stage and shorter 2-SEK stage

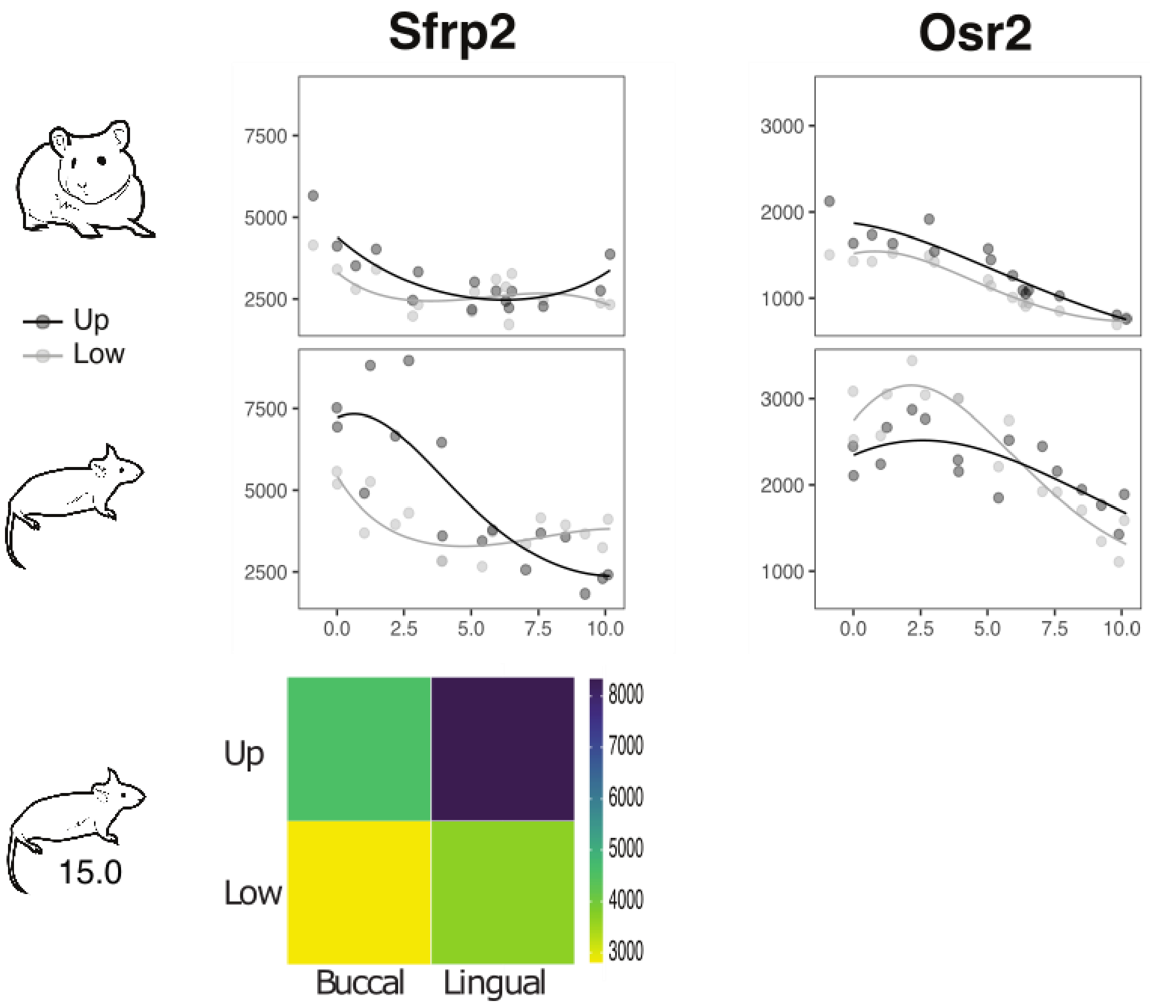

Supplementary Figure **3: *Sfrp2* and *Osr2* transcriptomic profiles**

Top: transcriptomic profiles in mouse and hamster. Each dot represents one sample. x: relative developmental time y: RNAseq basemeans.

Bottom: spatial profile in the bucco-lingual dataset at 15.0 dpc in mouse. The colour scale represents normalised read numbers for each gene, both are more strongly expressed in the lingual side.

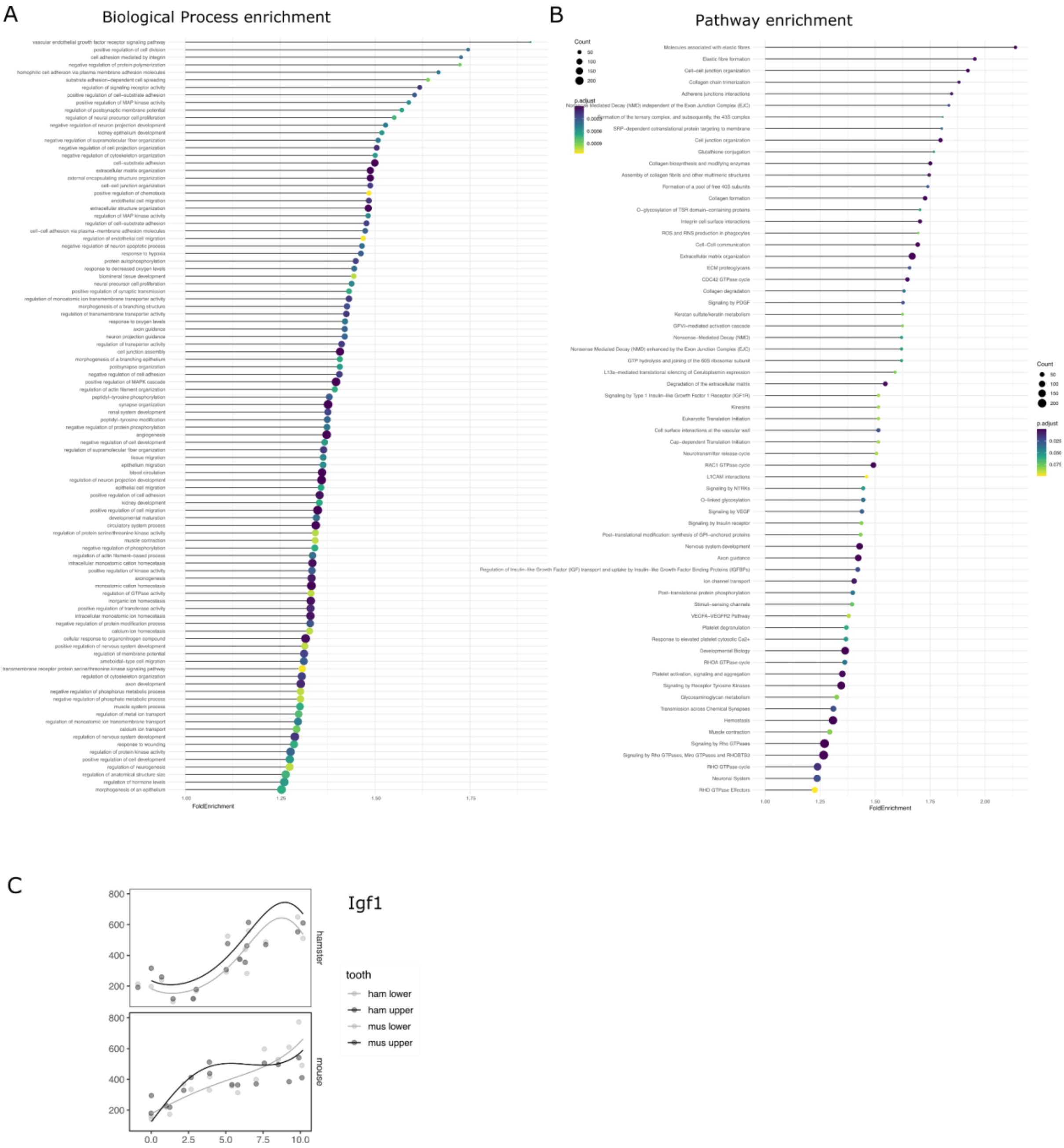

Supplementary Figure **4:** ***Gene ontology analysis of co-evolving genes***

A: Biological process enrichment for the 4970 co-evolving genes detected in Fig4.

B: Pathway enrichment for the 4970 co-evolving genes detected in Fig4.

C: IGF-1 gene transcriptional profile in this study.

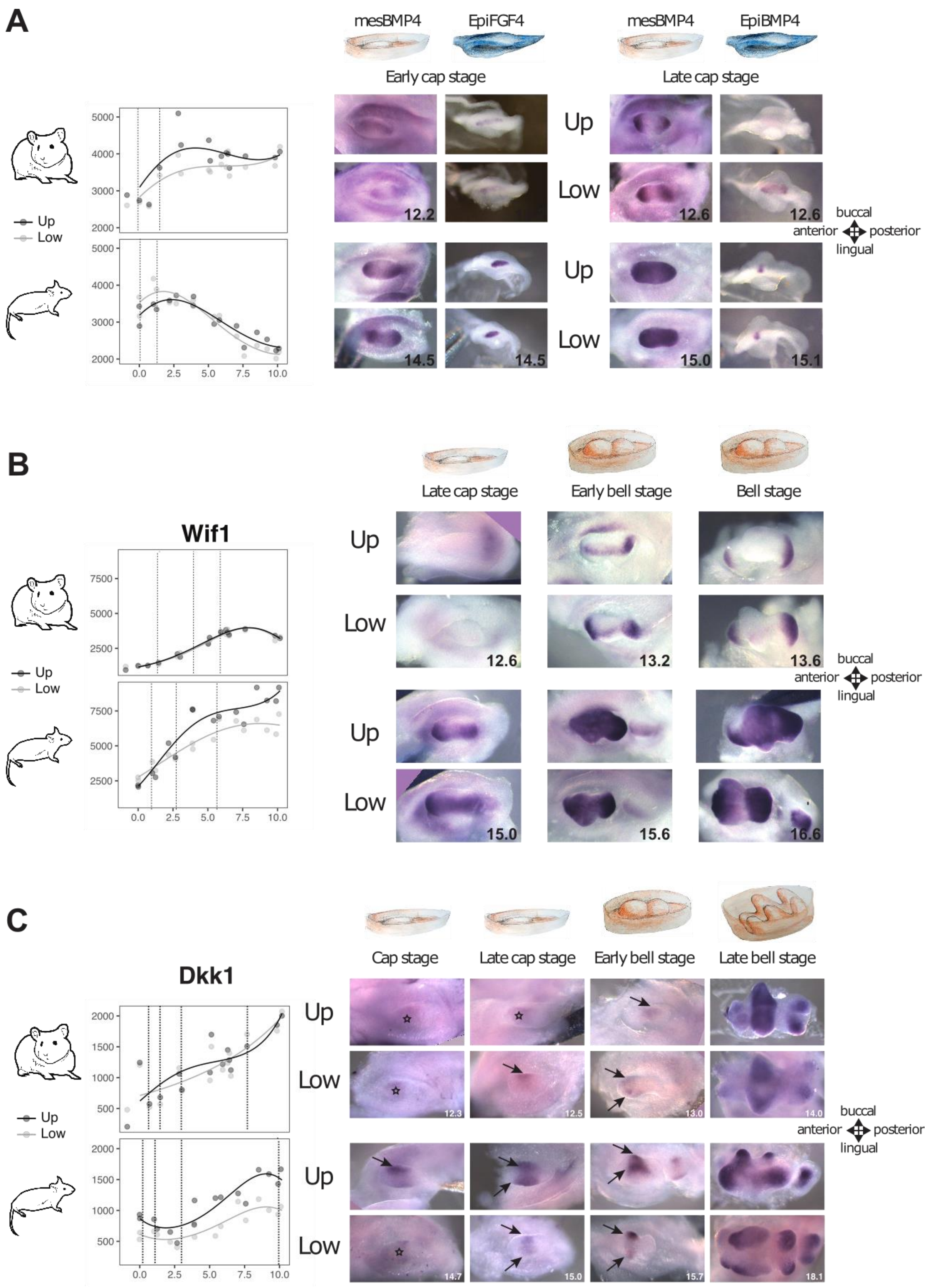

Supplementary Figure **5: The expression changes marking the initiation of cusp patterning are anticipated in mouse molar mesenchyme.**

A,B,C Left: Transcriptomic profiles for *Bmp4, Wif1* and *Dkk1*. Each dot represents one sample. x: relative developmental time y: RNAseq basemeans. Although *Bmp4* and *Wif1* are also expressed in the epithelium, the transcriptomic profile is mainly driven by the mesenchymal expression representing a larger number of cells. Dashed lines on the profile indicate timepoints of samples shown on the right.

A,B,C Right: top view of the mesenchyme and/or epithelium, hybridized with the indicated probe. Pictures are centered on the dental mesenchyme, whose initial ovoid shape progresses to a tooth shape through cusp formation.

A: Precocious expression of *Bmp4* in mouse molars. Left: *Bmp4* expression is higher and peaks earlier in both mouse molars. Right: in situ hybridization for a *Bmp4* or *Fgf4* probe, on mesenchymal (left columns) or epithelial (right columns) parts of cap stage tooth germs taken early or late, at similar advancement of epithelial caping for mouse and hamster (compare epithelia). Shortly after cap transition, *Bmp4* mesenchymal expression is low in the hamster, but already strong in the anterior and posterior part of the mouse dental mesenchyme. Later expression is increased in the hamster and resembles early cap stage in the mouse, but in the mouse, *Bmp4* is now seen in the whole dental mesenchyme.

B-Left: *Wif1* expression is higher in mouse and increases earlier as compared to hamster. Right: In situ hybridization of confirmed the more precocious and ubiquitous expression of *Wif1* in mouse.

C: Focused expression of the Wnt inhibitor *Dkk1* is observed earlier in mouse molars, especially in mouse upper molar. Left: *Dkk1* is more highly expressed in mouse upper molars (cusp model as in Figure 4D, adjusted p-value = 0.01). Right: Consistent with the transcriptomic profile, *Dkk1* expression is higher in the upper mouse molar relative to its lower counterpart throughout development. At early cap stage, *Dkk1* expression is well focused (arrow) buccally in the mouse upper molar, where the first upper molar cusp forms. It is more diffusely expressed (star) in the mouse lower molar mesenchyme, as in hamster molars. Focused *Dkk1* expression and cusp formation is delayed in the hamster as compared to mouse, for both teeth.

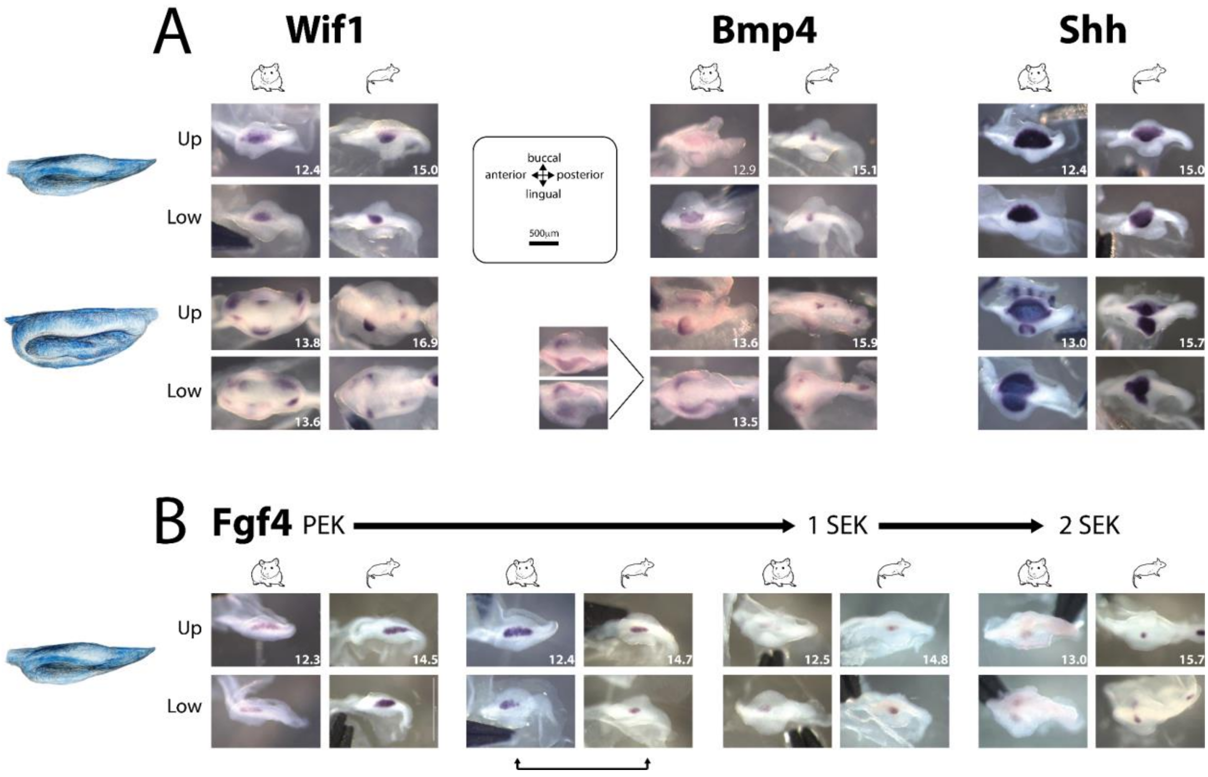

Supplementary Figure **6: Mouse molars show a precocious transition to SEK stage, with more focalized expression of signalling molecules.**

A: *Wif1*, *Bmp4* and *Shh* expression territories are more focalized in mouse SEKs. This is seen in early stages with a single SEK (left) or in later stages with more SEKs (right). Mouse and hamster samples are paired for similar advancement of epithelial growth. In hamster, the upper molar is markedly delayed compared to lower molar, therefore when available, we selected slightly older upper samples for the upper molar. Numeric age (days) is indicated in the bottom right of the upper molar picture when lower and upper samples were taken from the same embryo, or in both pictures, when samples were taken from different embryos. For the sake of comparison, *Bmp4* samples shown in Figure 4A are shown next to *Wif1* and *Shh* samples.

B: Transition from the PEK to the 2 SEK stage as seen on tooth germ epithelial parts hybridised against *Fgf4*. Same samples as in Figure 3B, numeric age is added in the corner. Double arrow: The elongated PEK-like expression of *Fgf4* is still visible in hamster, but roundish SEK-like expression of *Fgf4* is already seen in mouse.

Related to Figure 4/

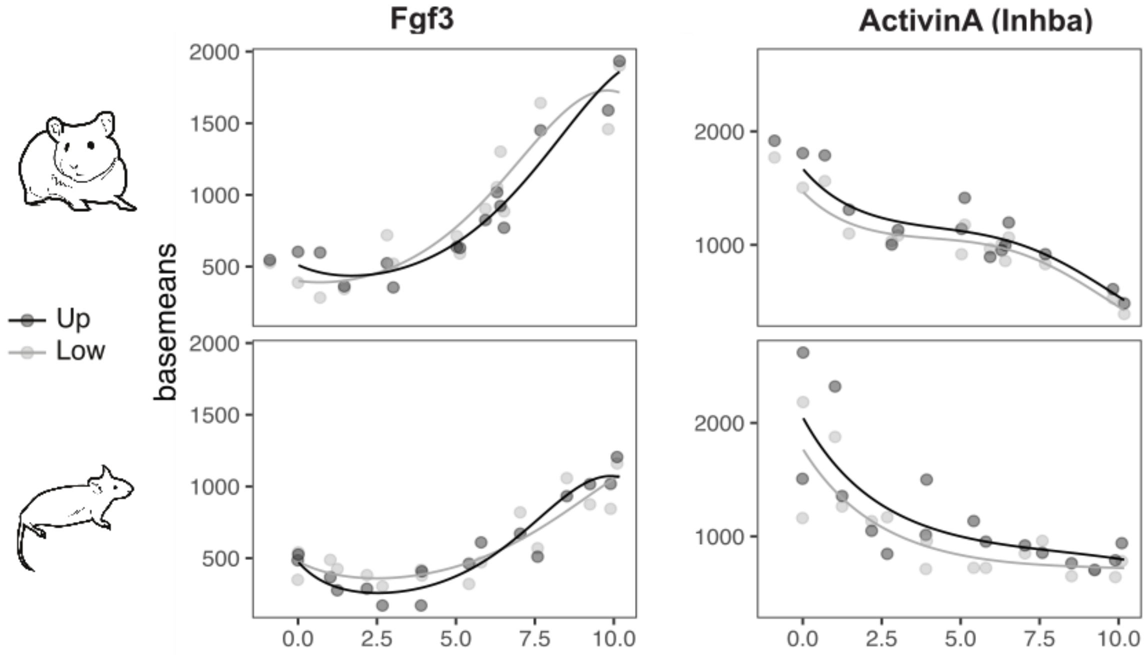

Supplementary Figure **7: Transcriptional profiles for *Fgf3* and *Inhba* (coding for ACTIVINβA) suggest that they are not involved in the mouse upper molar evolution.** Below we explain why these genes were potential candidates based on the literature and why we rule them out.

Fgf3 - It was shown previously that in a heterozygous mutant for the *Fgf3* gene, the first upper molar is smaller and loses its most anterior supplementary cusp, converted into a crest, as seen for the first murine rodents appearing in the paleontological record (*Potwarmus* genus, [57]). In the homozygous mutant, the first upper molar is even smaller and its morphology is severely altered: a longitudinal crest is present, as in murine ancestors or in hamster (the central cusps linked by this crest are malformed), the most anterior cusp is totally suppressed (no crest) but the posterior supplementary cusp is present and large. The overall morphology is thus very far from any ancestral shape, but these shape modifications suggest that making supplementary cusps is developmentally associated with suppressing the longitudinal crest. The molar size is markedly reduced. The suppression of the supplementary cusps is unsurprising, as they form last and therefore will mechanically be the most affected by any reduction of tooth growth. The authors propose that mouse upper molar evolution may have relied on increased FGF3 activity. In our transcriptomes, *Fgf3* showed no increased expression in mouse as compared to hamster (nor in lower/upper mouse molar). The expression levels and profiles are remarkably similar during the cap and bell stage, and hamster expression levels increase more rapidly during the late bell stage than they do in mouse. This is consistent with a role of *Fgf3* in crown growth, the crown being higher in hamster, but suggests that *Fgf3* was not involved in murine dental plan evolution.

Inhba - It was shown that adding ActivinβA to the culture medium of lower molars induces the formation of small supplementary cusps on the lingual side [16]. In our transcriptomes, the mean expression level and profile are not significantly different between mouse and hamster (nor lower and upper molar in each species).
