# Supplementary material for: Comparative transcriptomics in serial organs uncovers early and pan-organ developmental changes associated with organ-specific morphological adaptation
Source: Nat Commun. 2025 Jan 17;16:768. doi: 10.1038/s41467-025-55826-w (PMC11742040; doi:10.1038/s41467-025-55826-w)
Supplement: Supplementary file 2 — Description of Additional Supplementary Files [file 41467_2025_55826_MOESM2_ESM.pdf]

Description of Additional Supplementary Files:

Supplementary Data 1: an excel file with results of the tests performed in Figure 4.
