# Supplementary material for: Comparative transcriptomics in serial organs uncovers early and pan-organ developmental changes associated with organ-specific morphological adaptation
Source: Nat Commun. 2025 Jan 17;16:768. doi: 10.1038/s41467-025-55826-w (PMC11742040; doi:10.1038/s41467-025-55826-w)
Supplement: Supplementary file 4 — Reporting Summary [file 41467_2025_55826_MOESM4_ESM.pdf]

nature portfolio

Corresponding author(s): Sophie PANTALACCI

Last updated by author(s): Aug 6, 2024

Reporting Summary

Nature Portfolio wishes to improve the reproducibility of the work that we publish. This form provides structure for consistency and transparency in reporting. For further information on Nature Portfolio policies, see our Editorial Policies and the Editorial Policy Checklist.

Statistics

For all statistical analyses, confirm that the following items are present in the figure legend, table legend, main text, or Methods section.

n/a	Confirmed	
☐	☒	The exact sample size (*n*) for each experimental group/condition, given as a discrete number and unit of measurement
☐	☒	A statement on whether measurements were taken from distinct samples or whether the same sample was measured repeatedly
☐	☒	The statistical test(s) used AND whether they are one- or two-sided<br>*Only common tests should be described solely by name; describe more complex techniques in the Methods section.*
☒	☐	A description of all covariates tested
☐	☒	A description of any assumptions or corrections, such as tests of normality and adjustment for multiple comparisons
☐	☒	A full description of the statistical parameters including central tendency (e.g. means) or other basic estimates (e.g. regression coefficient) AND variation (e.g. standard deviation) or associated estimates of uncertainty (e.g. confidence intervals)
☐	☒	For null hypothesis testing, the test statistic (e.g. *F*, *t*, *r*) with confidence intervals, effect sizes, degrees of freedom and *P* value noted<br>*Give P values as exact values whenever suitable.*
☐	☒	For Bayesian analysis, information on the choice of priors and Markov chain Monte Carlo settings
☐	☒	For hierarchical and complex designs, identification of the appropriate level for tests and full reporting of outcomes
☒	☐	Estimates of effect sizes (e.g. Cohen's *d*, Pearson's *r*), indicating how they were calculated

Our web collection on statistics for biologists contains articles on many of the points above.

Software and code

Policy information about availability of computer code

Data collection	Data collection did not involve a code.
Data analysis	All custom code (run in R) used in this study are made available for each figure, together with the accompagnying data, so that all the panels can be reproduced. They were uplodad as Data source file. (zip folder containing one folder per figure, with code and data).<br>see also https://github.com/msemon/DriftHamsterMouse.<br><br>We used the following custom codes:<br>- the custom code for reconstructing the temporal dynamics from a series of fixed embryos with MMC modeling is available in the folder related to Fig. 1.<br>- the custom code for modeling temporal profiles with spline coefficient and test nested models is available in the folder related to Fig2 and 4.<br>We also used the following packages (see the corresponding figure folder):<br>- mapping and cropping on orthologous gene portions: Kallisto (version 0.44.0) and macse_v2.01<br>- DE analysis: DEseq2<br>- functional enrichment (clusterProfiler (version 4.10.1), enrichplot (1.22.0) and ReactomePA (1.46.0))<br>- measuring pathway activation ROMA (rRoma_0.0.4.2000, https://github.com/Albluca/rRoma)<br>- deconvolutions: DeconRNASeq (DeconRNASeq_1.32.0

For manuscripts utilizing custom algorithms or software that are central to the research but not yet described in published literature, software must be made available to editors and reviewers. We strongly encourage code deposition in a community repository (e.g. GitHub). See the Nature Portfolio guidelines for submitting code & software for further information.

Data

Policy information about availability of data

All manuscripts must include a data availability statement. This statement should provide the following information, where applicable:

- Accession codes, unique identifiers, or web links for publicly available datasets
- A description of any restrictions on data availability
- For clinical datasets or third party data, please ensure that the statement adheres to our policy

Raw data are publically available in ENA with project accession number: PRJEB52633.
All quantitative data in this manuscript have been made available for each figure in the data source fille: zip folder containing one folder per figure, with code and data .
See also https://github.com/msemon/DriftHamsterMouse.
See also supplementary file 1 and supplementary tables 1 and 2.

Research involving human participants, their data, or biological material

Policy information about studies with human participants or human data. See also policy information about sex, gender (identity/presentation), and sexual orientation and race, ethnicity and racism.

Reporting on sex and gender	Research did not involve human participants, data or biological material
Reporting on race, ethnicity, or other socially relevant groupings	Research did not involve human participants, data or biological material
Population characteristics	Research did not involve human participants, data or biological material
Recruitment	Research did not involve human participants, data or biological material
Ethics oversight	Research did not involve human participants, data or biological material

Note that full information on the approval of the study protocol must also be provided in the manuscript.

Field-specific reporting

Please select the one below that is the best fit for your research. If you are not sure, read the appropriate sections before making your selection.

☒ Life sciences ☐ Behavioural & social sciences ☐ Ecological, evolutionary & environmental sciences

For a reference copy of the document with all sections, see nature.com/documents/nr-reporting-summary-flat.pdf

Life sciences study design

All studies must disclose on these points even when the disclosure is negative.

Sample size	No sample-size calculation<br>Two replicates for the transcriptomic study were enough to very efficiently model the temporal dynamics of the vast majority of genes with spline coefficients.<br>Due to ethical reasons, sample size for in situ hybridization and 3D reconstruction were chosen as the minimal number of samples to achieve convincing description of the tooth germ and/or gene expression dynamics.
Data exclusions	no data exclusion
Replication	Samples for transcriptomics were taken in duplicates + with close timepoints, so that the temporal continuity also testifies for the quality of the sample and/or robustness of the result.<br>For in situ hybridizations, each timepoint was validated with at least an embryo of similar weight and several contiguous stages were used, so that signal continuity also served as a control.<br>For 3D reconstructions, a minimum of 3 replicates was used in an homologous time window, all were in agreement with bioinformatic results.
Randomization	not relevant in this study
Blinding	not relevant in this study

Reporting for specific materials, systems and methods

We require information from authors about some types of materials, experimental systems and methods used in many studies. Here, indicate whether each material, system or method listed is relevant to your study. If you are not sure if a list item applies to your research, read the appropriate section before selecting a response.

Materials & experimental systems

n/a	Involved in the study
☐	☒ Antibodies
☒	☐ Eukaryotic cell lines
☒	☐ Palaeontology and archaeology
☐	☒ Animals and other organisms
☒	☐ Clinical data
☒	☐ Dual use research of concern
☒	☐ Plants

Methods

n/a	Involved in the study
☒	☐ ChIP-seq
☒	☐ Flow cytometry
☒	☐ MRI-based neuroimaging

Antibodies

Antibodies used	Primary - Mouse-P cadherin (goat) antibody, R&D Systems, #AF761<br>Secondary - Alexa Fluor 488 Donkey anti-goat antibody, Jacson Immunoresearch #705-545-147
Validation	validated antibodies from the manufacturer

Animals and other research organisms

Policy information about studies involving animals; ARRIVE guidelines recommended for reporting animal research, and Sex and Gender in Research

Laboratory animals	Mouse: mus musculus domesticus CD1strain from Charles River Laooratories<br>Hamster: mesocricetus auratus RjHan:AURA from Janvier
Wild animals	*Provide details on animals observed in or captured in the field; report species and age where possible. Describe how animals were caught and transported and what happened to captive animals after the study (if killed, explain why and describe method; if released, say where and when) OR state that the study did not involve wild animals.*
Reporting on sex	Only female embryos were used for the transcriptomic dataset.<br>Embryos from both sexes (unknown) were used for the rest of the study as there is no morphological difference between male and female molars in these species.
Field-collected samples	NA
Ethics oversight	This study was performed in strict accordance with the European guidelines 2010/63/UE and was approved by the Animal Experimentation Ethics Committee CECCAPP (Lyon, France).

Note that full information on the approval of the study protocol must also be provided in the manuscript.

Plants

Seed stocks	no plant research in this study
Novel plant genotypes	no plant research in this study
Authentication	no plant research in this study
