# Supplementary material for: Comparative transcriptomics in serial organs uncovers early and pan-organ developmental changes associated with organ-specific morphological adaptation
Source: Nat Commun. 2025 Jan 17;16:768. doi: 10.1038/s41467-025-55826-w (PMC11742040; doi:10.1038/s41467-025-55826-w)
Supplement: Supplementary file 6 — Source Data [file 41467_2025_55826_MOESM6_ESM.zip › source data/Code_et_data_for_Fig/Code_and_data_for_fig6/Shh_fig6_panelA.pdf]

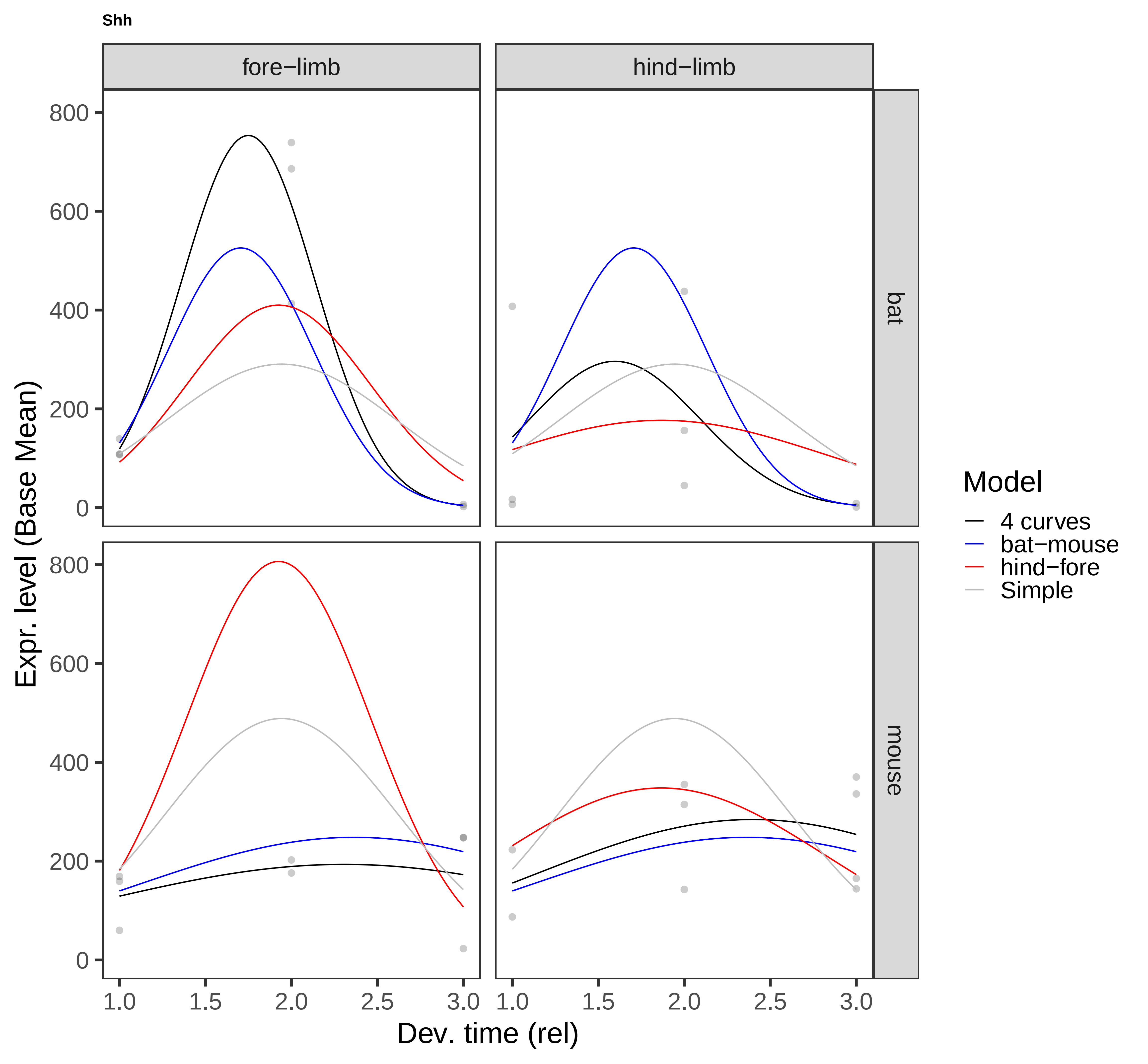

Shh
fore−limb
hind−limb
bat
mouse
Expr. level (Base Mean)
800
600
400
200
0
1.0
1.5
2.0
2.5
3.0
Dev. time (rel)
Model
4 curves
bat−mouse
hind−fore
Simple
