# Supplementary figures and images for: Comparative transcriptomics in serial organs uncovers early and pan-organ developmental changes associated with organ-specific morphological adaptation
Source: Nat Commun. 2025 Jan 17;16:768. doi: 10.1038/s41467-025-55826-w (PMC11742040; doi:10.1038/s41467-025-55826-w)

Biological Process enrichment

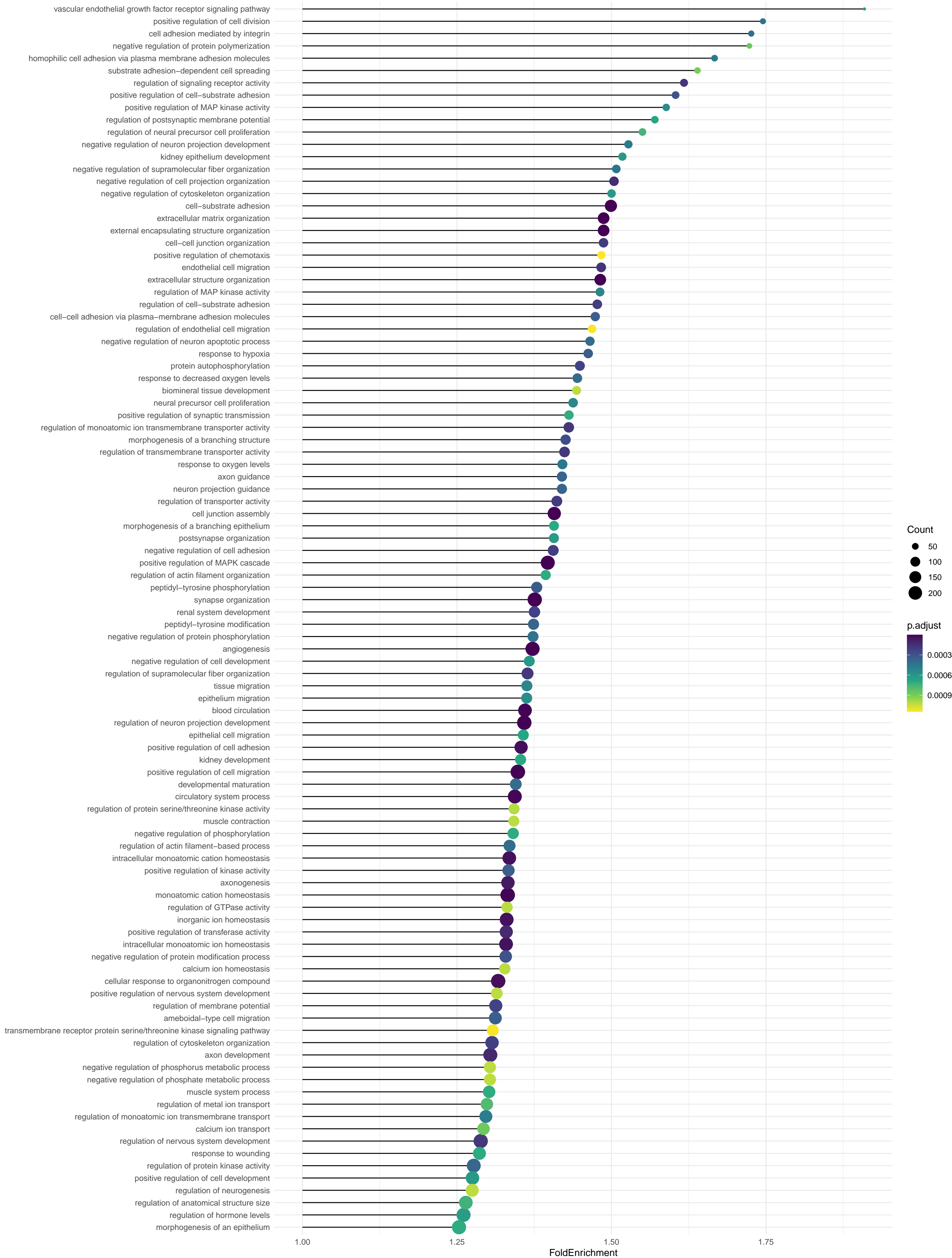

Supplement: Supplementary file 6 — Source Data [file 41467_2025_55826_MOESM6_ESM.zip › source data/Code_et_data_for_Fig/Code_and_data_for_Sfig4/FigS4_panelA_v2.pdf]

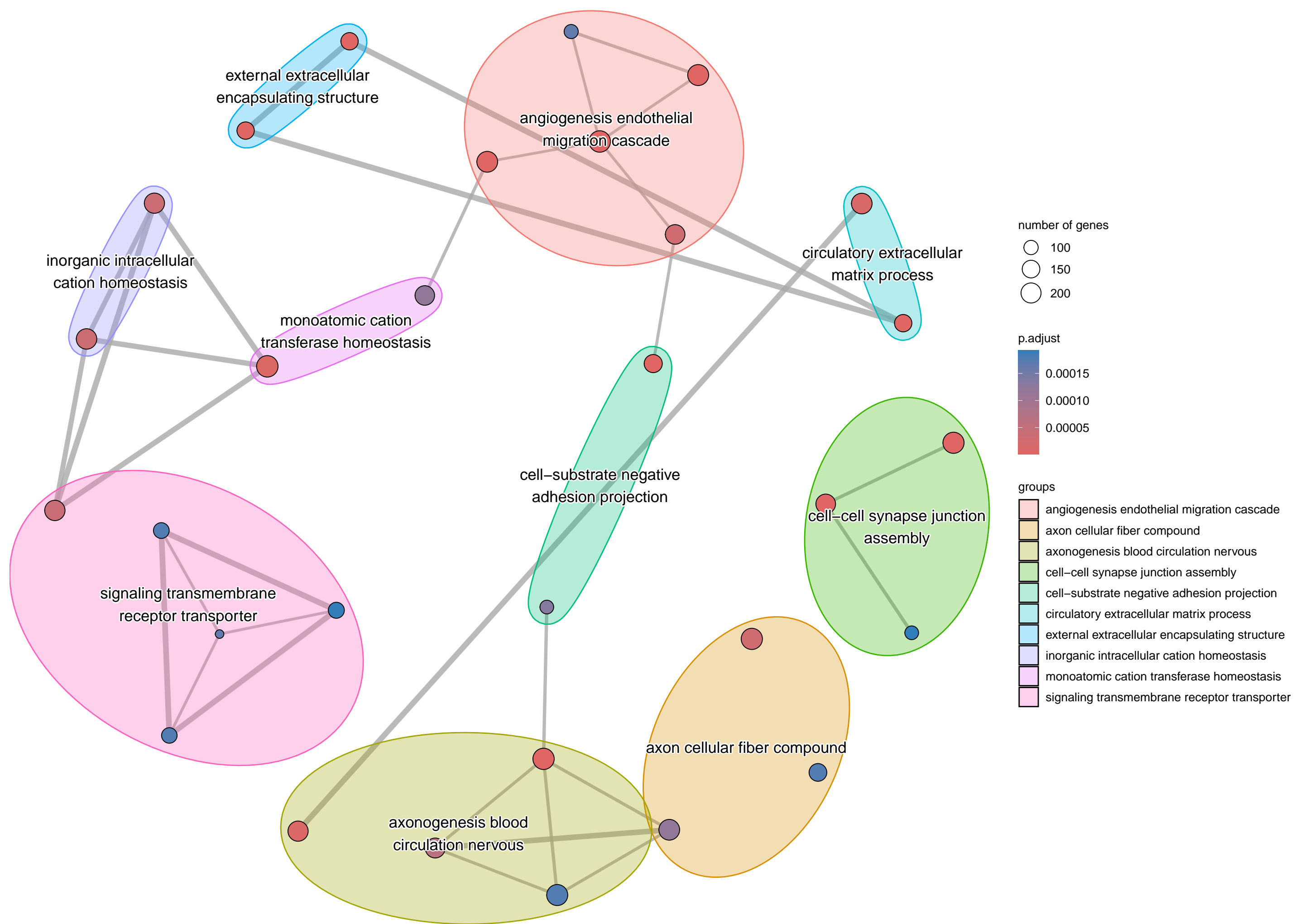

Supplement: Supplementary file 6 — Source Data [file 41467_2025_55826_MOESM6_ESM.zip › source data/Code_et_data_for_Fig/Code_and_data_for_Sfig4/FigS4_panelA.pdf]

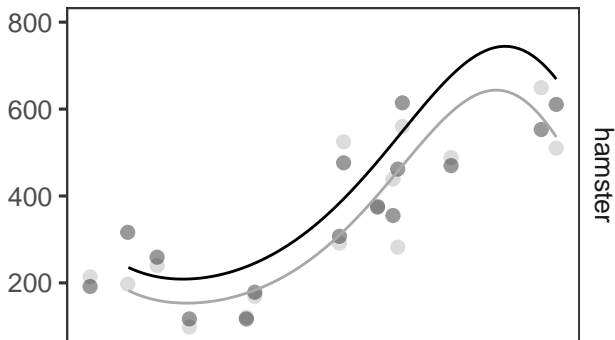

tooth

— ham lower

— ham upper

— mus lower

— mus upper

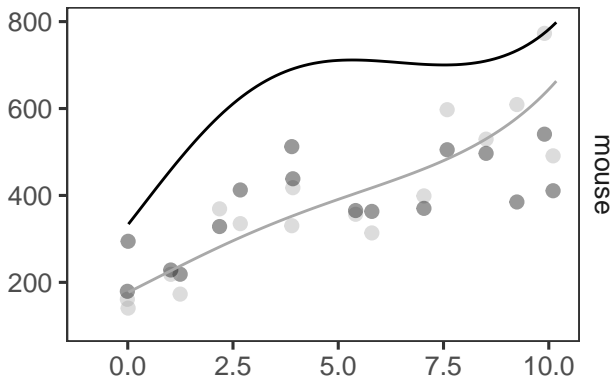

Supplement: Supplementary file 6 — Source Data [file 41467_2025_55826_MOESM6_ESM.zip › source data/Code_et_data_for_Fig/Code_and_data_for_Sfig4/FigS4_panelC.pdf]

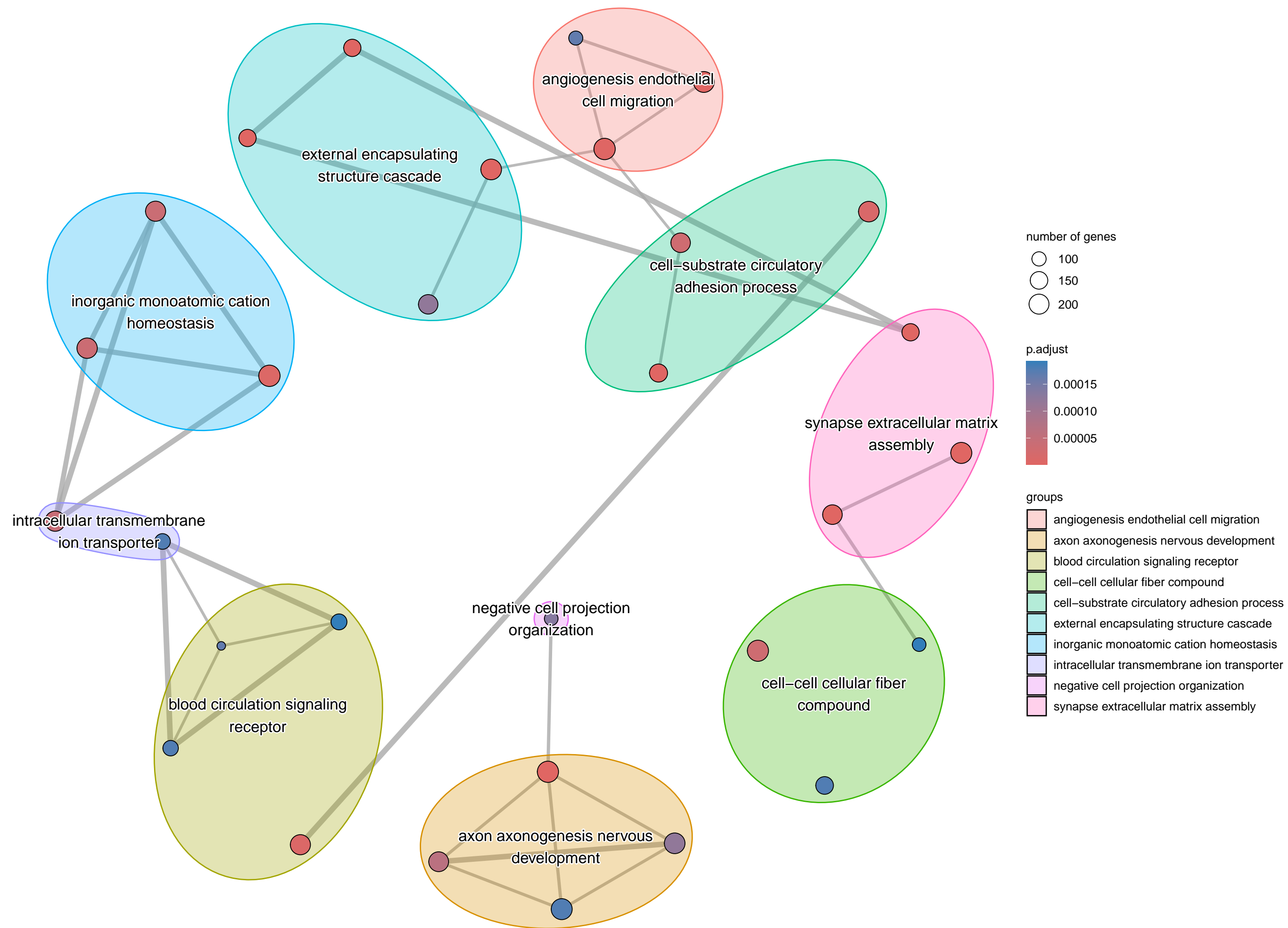

Supplement: Supplementary file 6 — Source Data [file 41467_2025_55826_MOESM6_ESM.zip › source data/Code_et_data_for_Fig/Code_and_data_for_Sfig4/FigS4_panelA_v1.pdf]

pathway enrichment

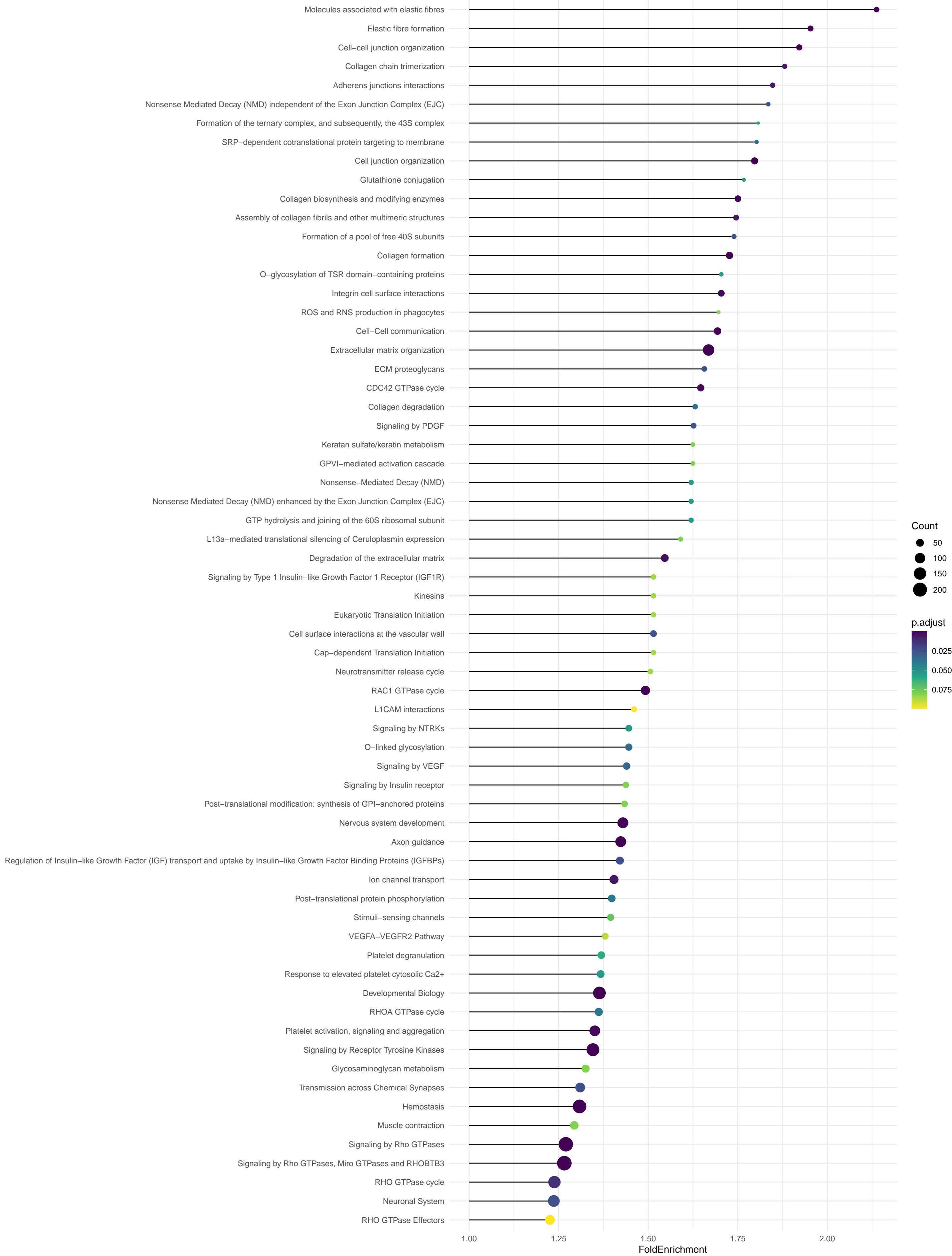

Supplement: Supplementary file 6 — Source Data [file 41467_2025_55826_MOESM6_ESM.zip › source data/Code_et_data_for_Fig/Code_and_data_for_Sfig4/FigS4_panelB.pdf]

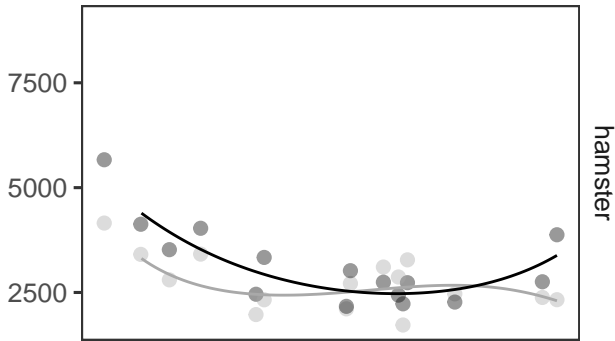

tooth

ham lower

ham upper

mus lower

mus upper

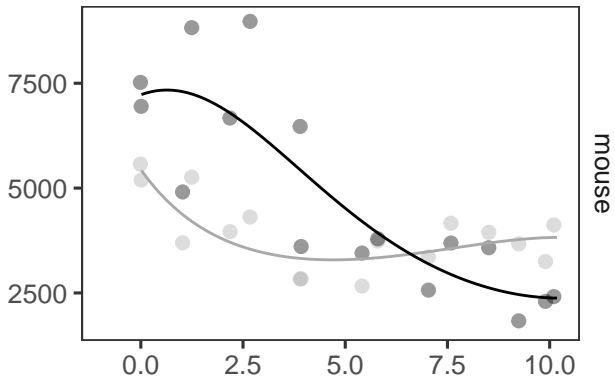

Supplement: Supplementary file 6 — Source Data [file 41467_2025_55826_MOESM6_ESM.zip › source data/Code_et_data_for_Fig/Code_and_data_for_Sfig3/figS3_panelB.pdf]

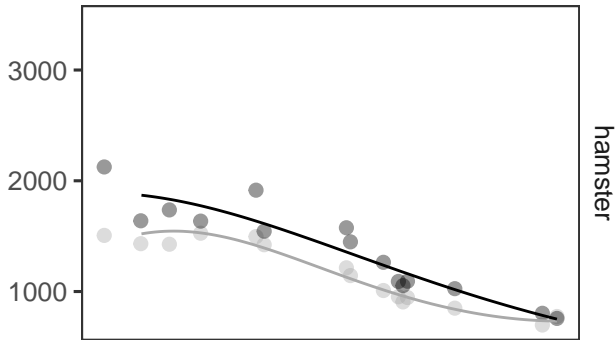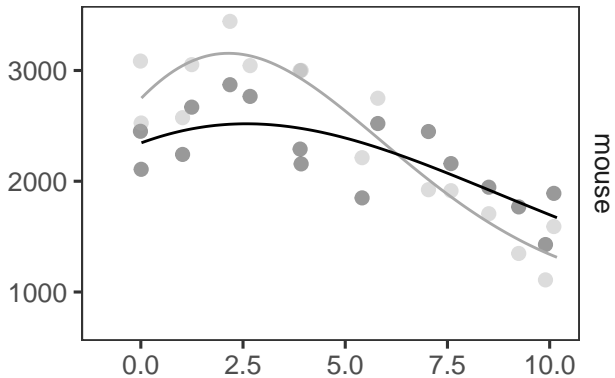

Supplement: Supplementary file 6 — Source Data [file 41467_2025_55826_MOESM6_ESM.zip › source data/Code_et_data_for_Fig/Code_and_data_for_Sfig3/figS3_panelA.pdf]

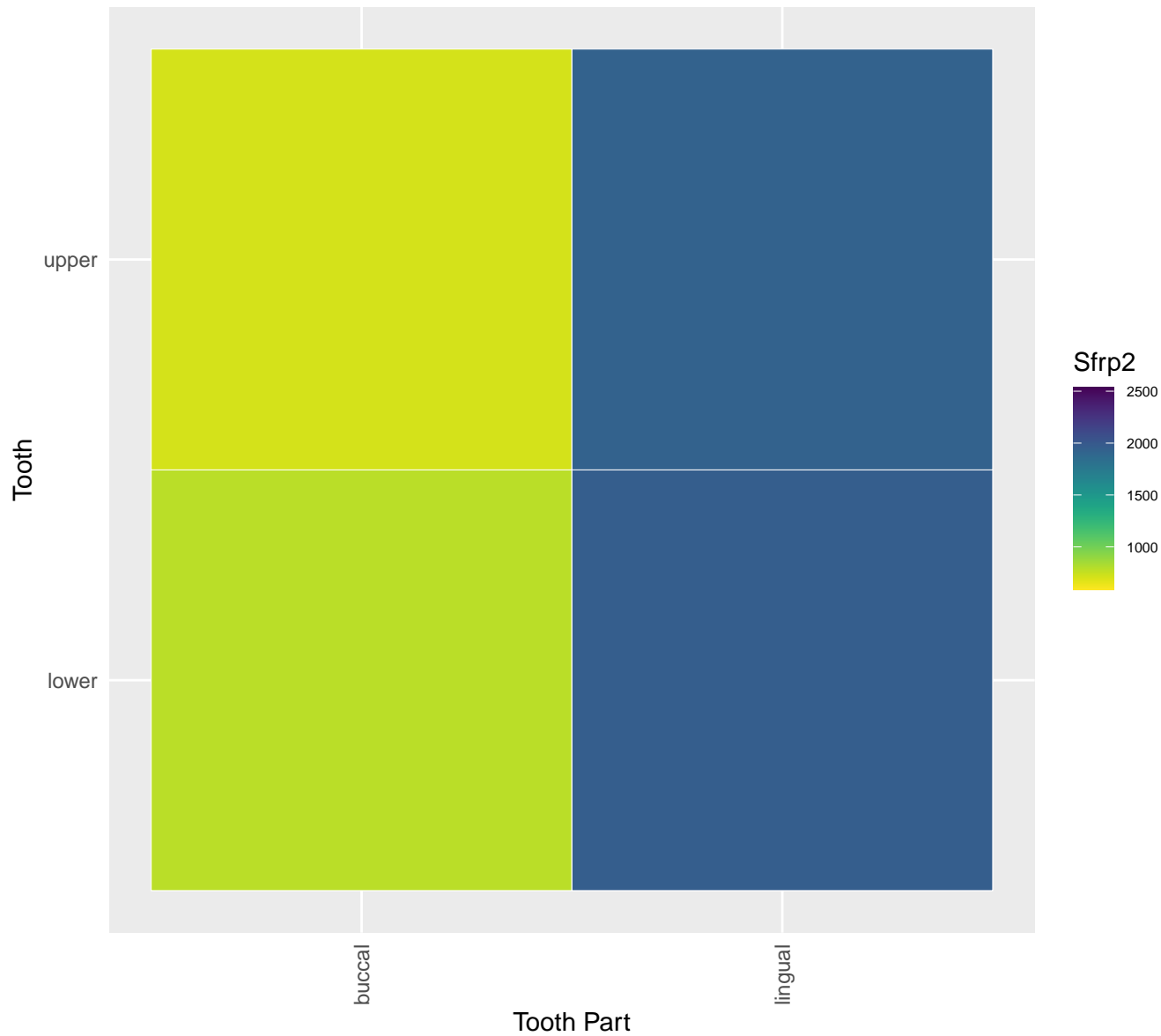

Supplement: Supplementary file 6 — Source Data [file 41467_2025_55826_MOESM6_ESM.zip › source data/Code_et_data_for_Fig/Code_and_data_for_Sfig3/FigS3_panel_bottom_Osr2.pdf]

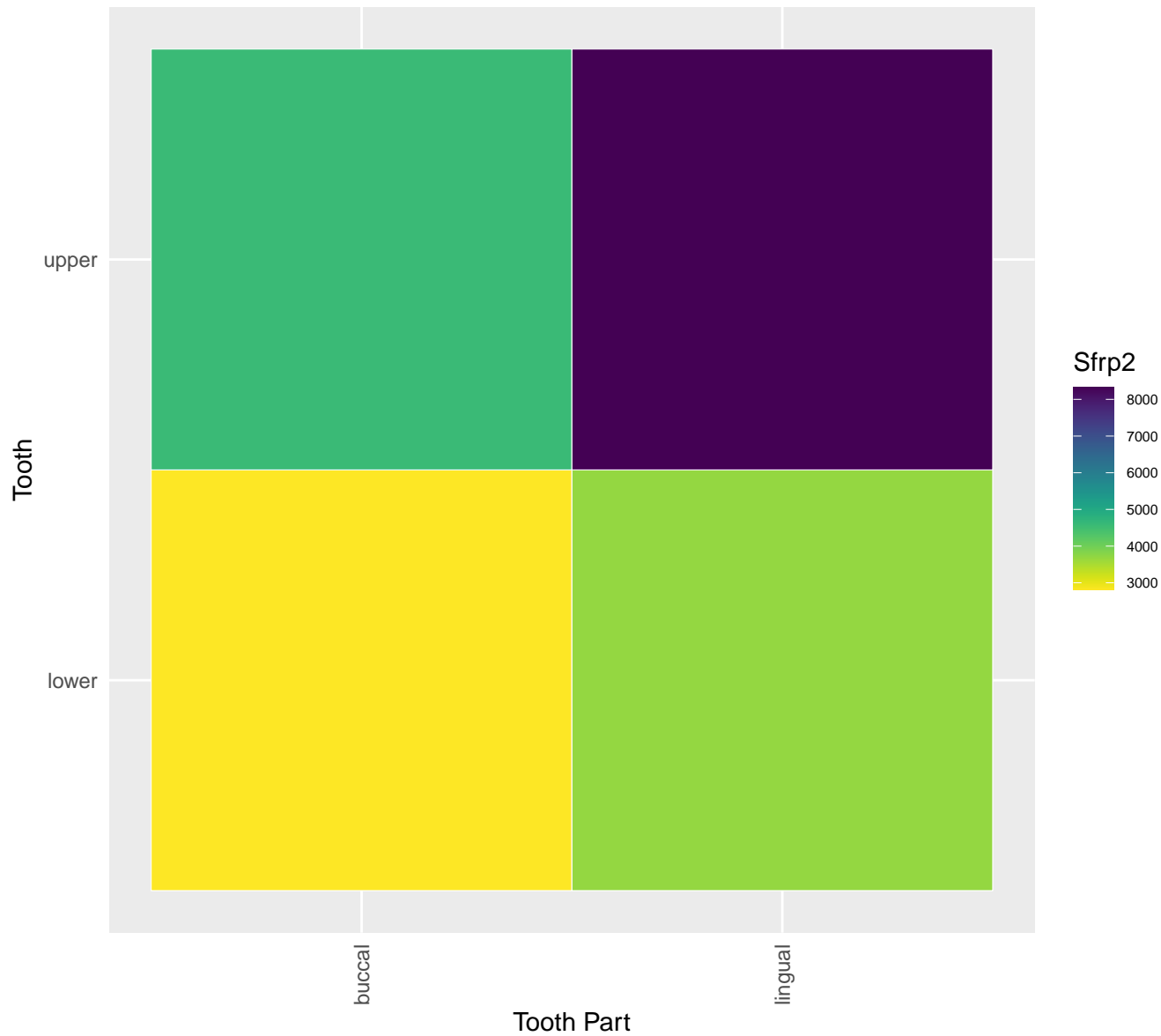

Supplement: Supplementary file 6 — Source Data [file 41467_2025_55826_MOESM6_ESM.zip › source data/Code_et_data_for_Fig/Code_and_data_for_Sfig3/FigS3_panel_bottom_Sfrp2.pdf]

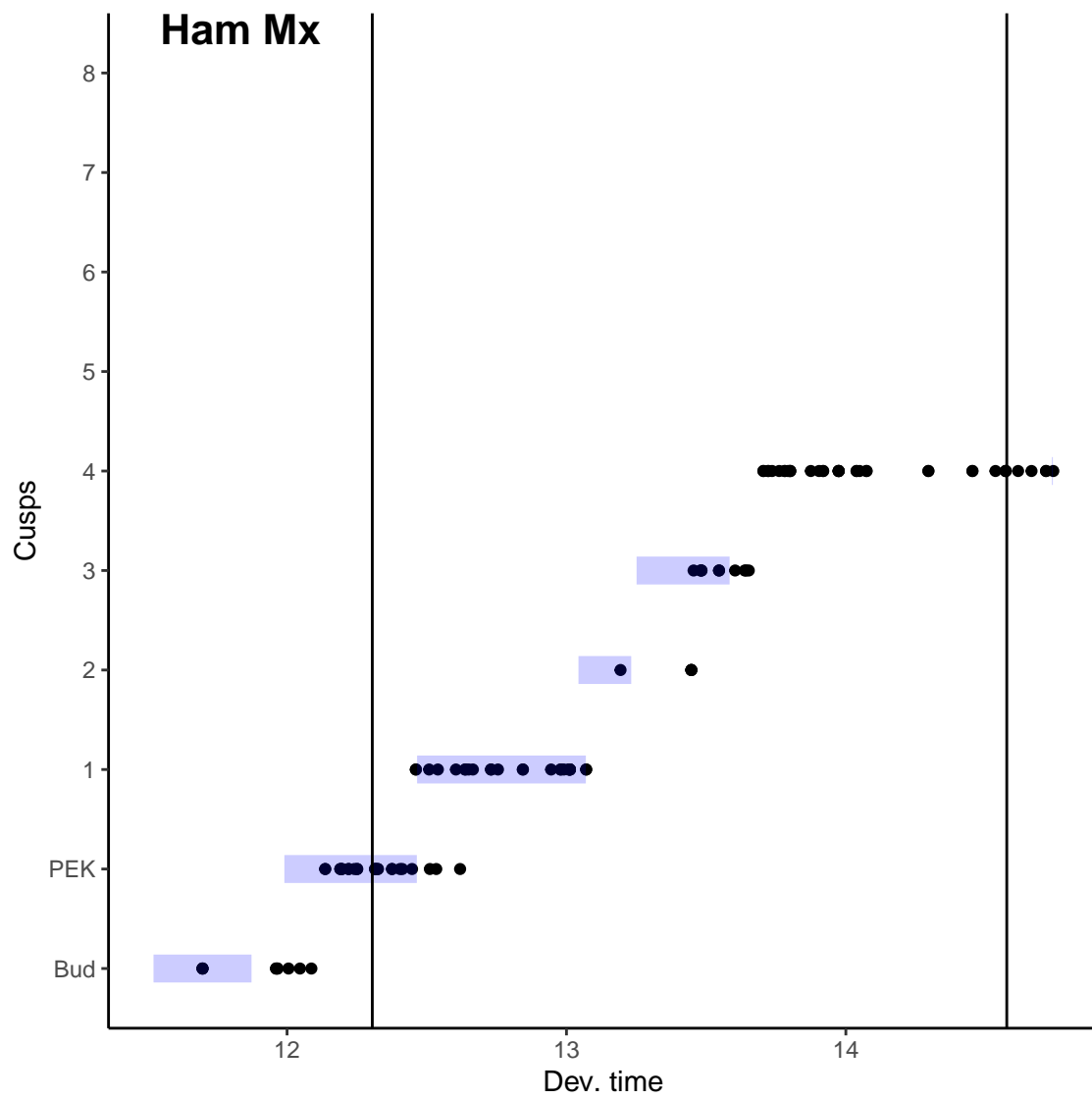

Supplement: Supplementary file 6 — Source Data [file 41467_2025_55826_MOESM6_ESM.zip › source data/Code_et_data_for_Fig/Code_and_data_for_Sfig2/figS2.pdf]

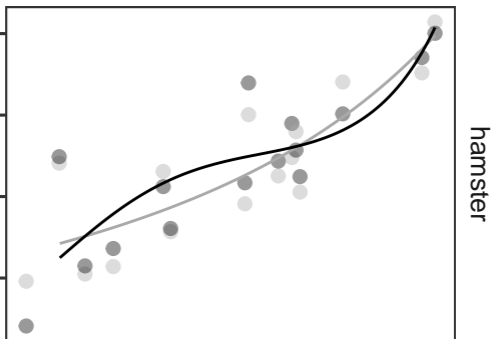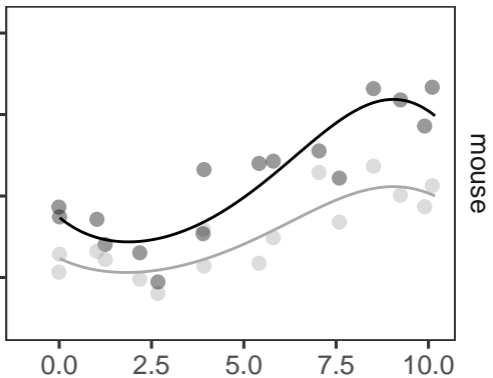

Supplement: Supplementary file 6 — Source Data [file 41467_2025_55826_MOESM6_ESM.zip › source data/Code_et_data_for_Fig/Code_and_data_for_Sfig5/Dkk1.pdf]

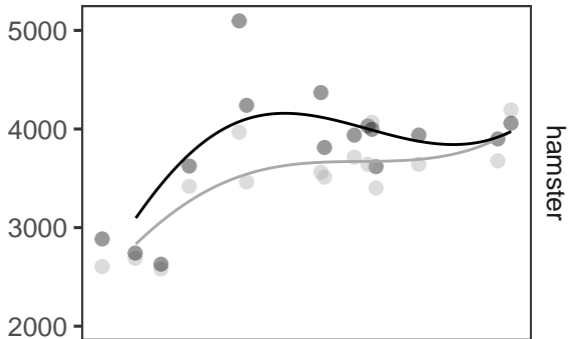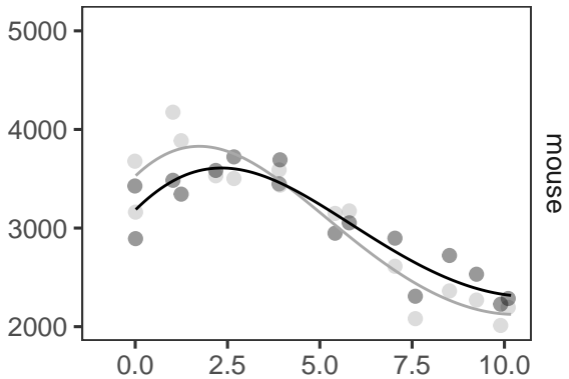

Supplement: Supplementary file 6 — Source Data [file 41467_2025_55826_MOESM6_ESM.zip › source data/Code_et_data_for_Fig/Code_and_data_for_Sfig5/Bmp4.pdf]

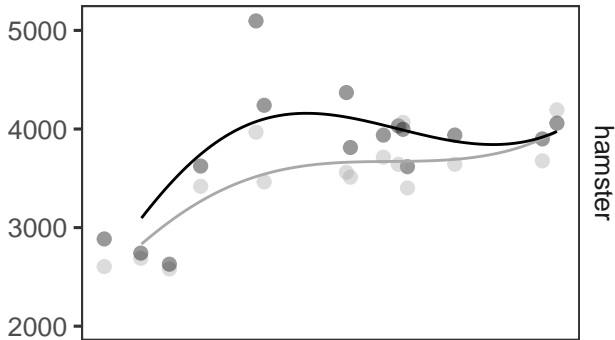

tooth

— ham lower

— ham upper

— mus lower

— mus upper

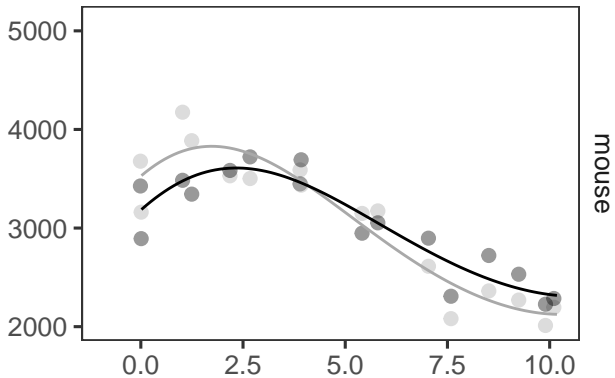

Supplement: Supplementary file 6 — Source Data [file 41467_2025_55826_MOESM6_ESM.zip › source data/Code_et_data_for_Fig/Code_and_data_for_Sfig5/figS5_panelA.pdf]

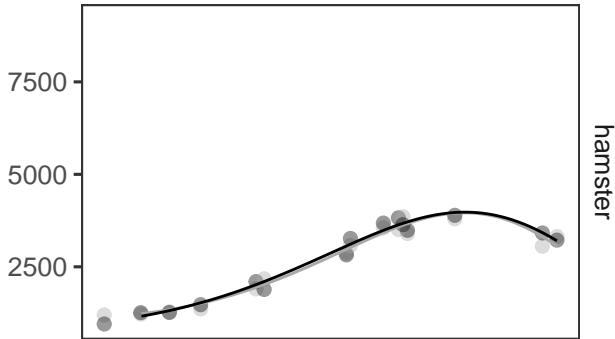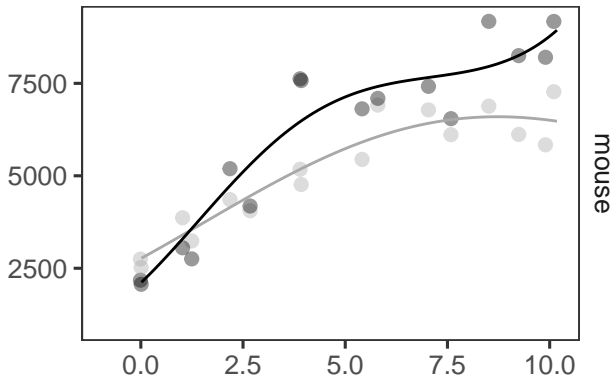

tooth

— ham lower

— ham upper

— mus lower

— mus upper

Supplement: Supplementary file 6 — Source Data [file 41467_2025_55826_MOESM6_ESM.zip › source data/Code_et_data_for_Fig/Code_and_data_for_Sfig5/figS5_panelC.pdf]

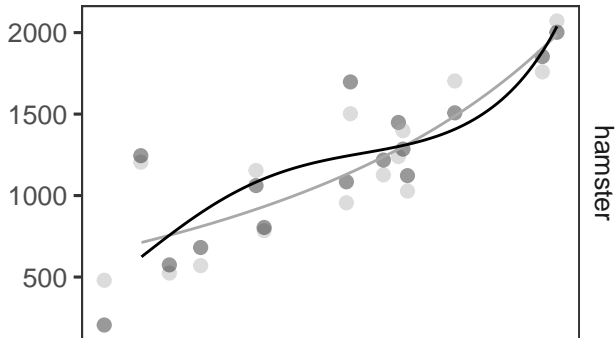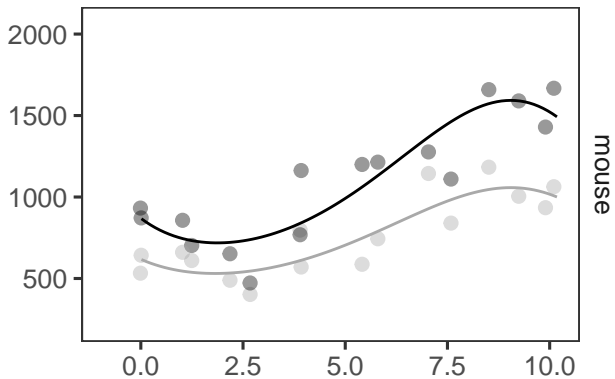

tooth

— ham lower

— ham upper

— mus lower

— mus upper

Supplement: Supplementary file 6 — Source Data [file 41467_2025_55826_MOESM6_ESM.zip › source data/Code_et_data_for_Fig/Code_and_data_for_Sfig5/figS5_panelB.pdf]

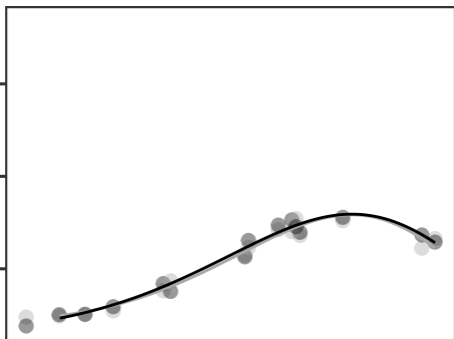

hamster

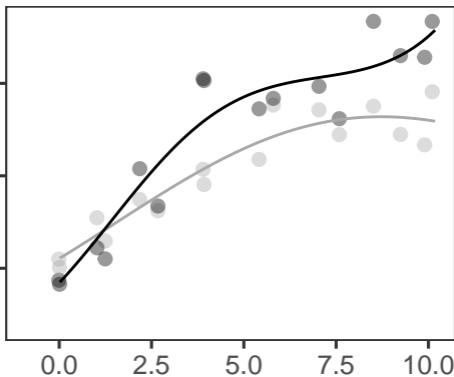

mouse

Supplement: Supplementary file 6 — Source Data [file 41467_2025_55826_MOESM6_ESM.zip › source data/Code_et_data_for_Fig/Code_and_data_for_Sfig5/Wif1.pdf]

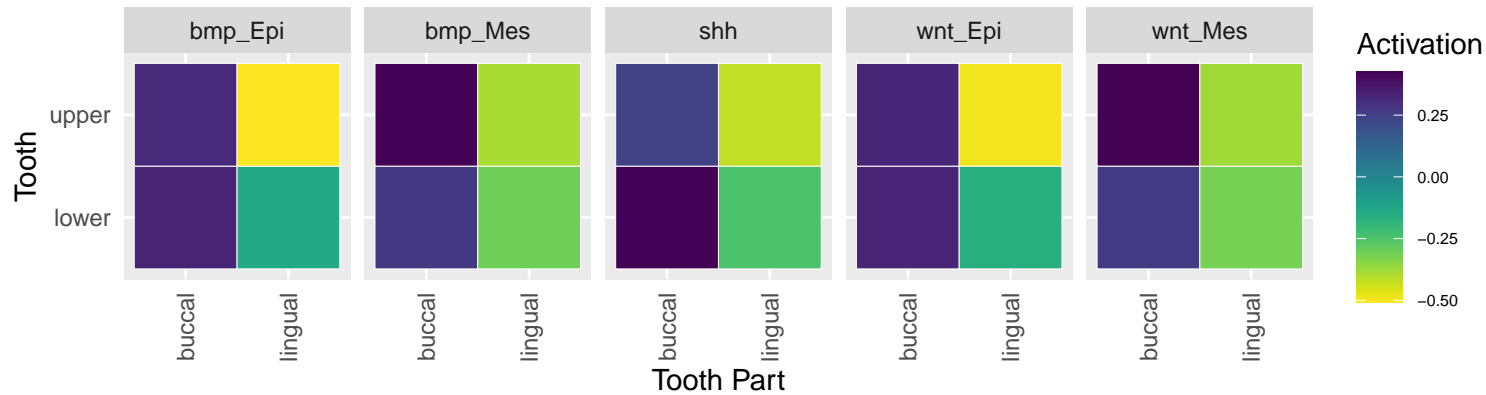

Supplement: Supplementary file 6 — Source Data [file 41467_2025_55826_MOESM6_ESM.zip › source data/Code_et_data_for_Fig/Code_and_data_for_fig3/fig3_panelC.pdf]

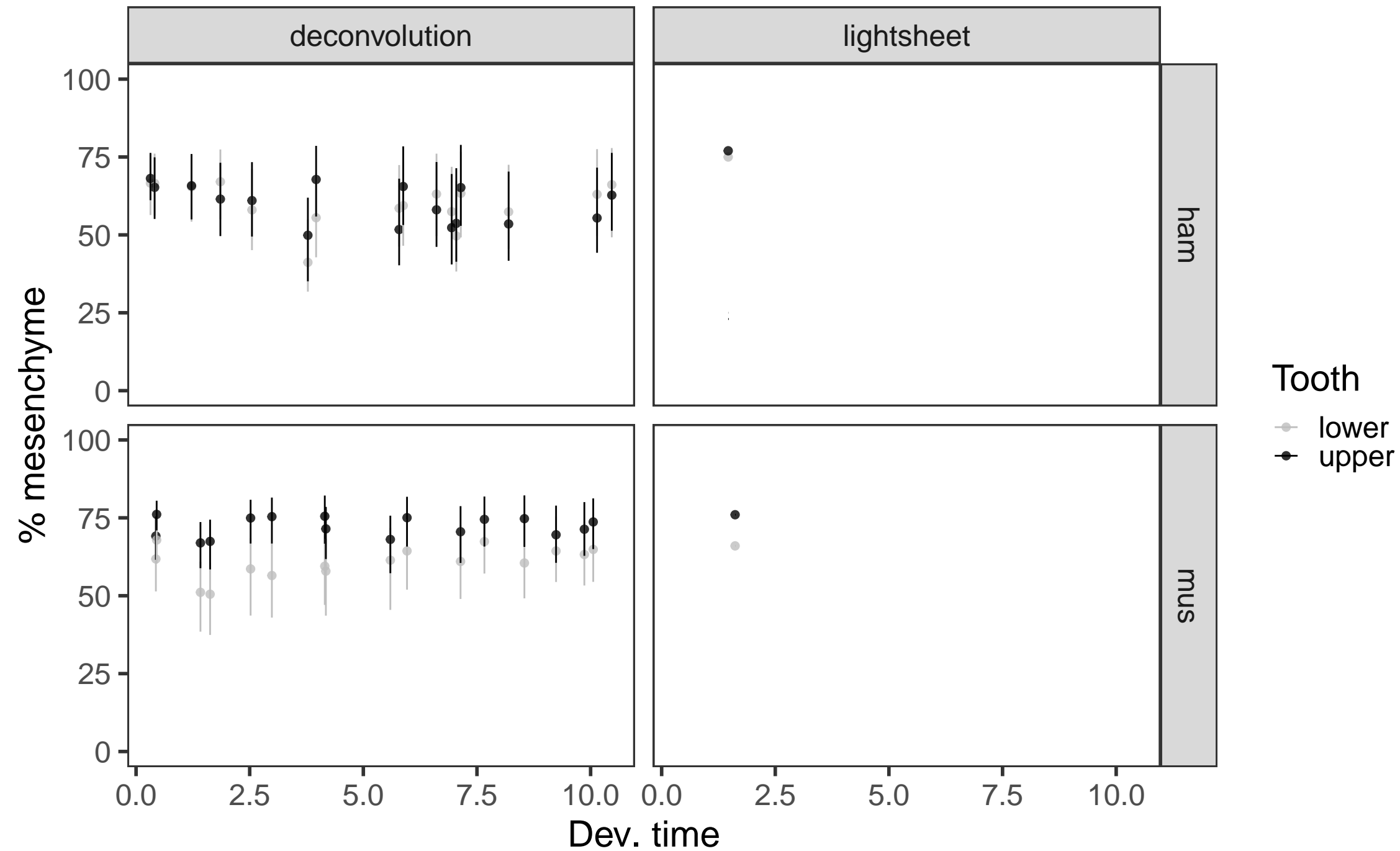

Supplement: Supplementary file 6 — Source Data [file 41467_2025_55826_MOESM6_ESM.zip › source data/Code_et_data_for_Fig/Code_and_data_for_fig3/fig3_panelA.pdf]

% lingual

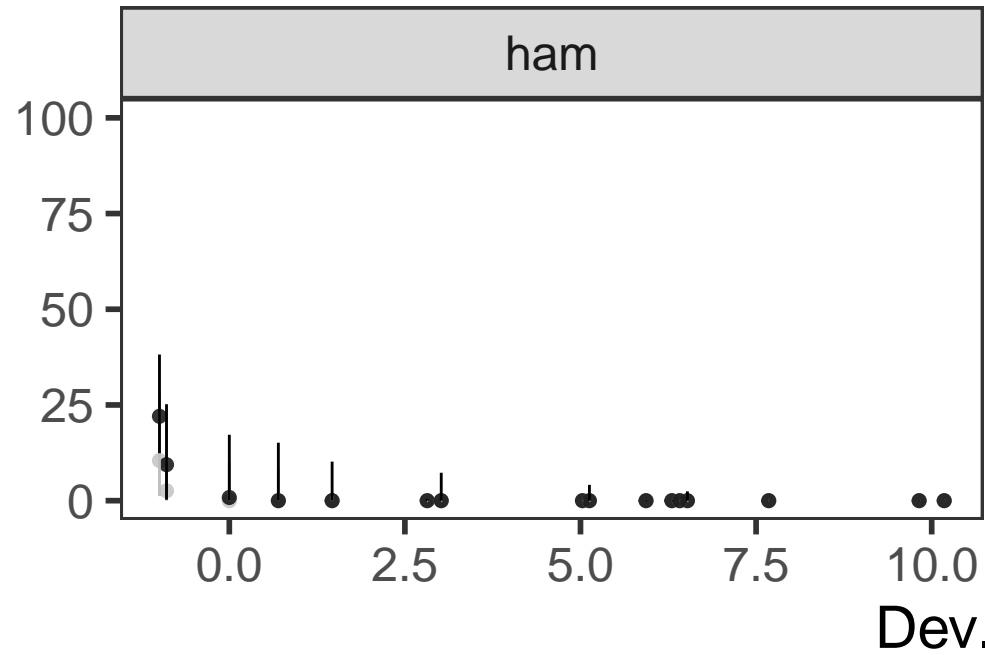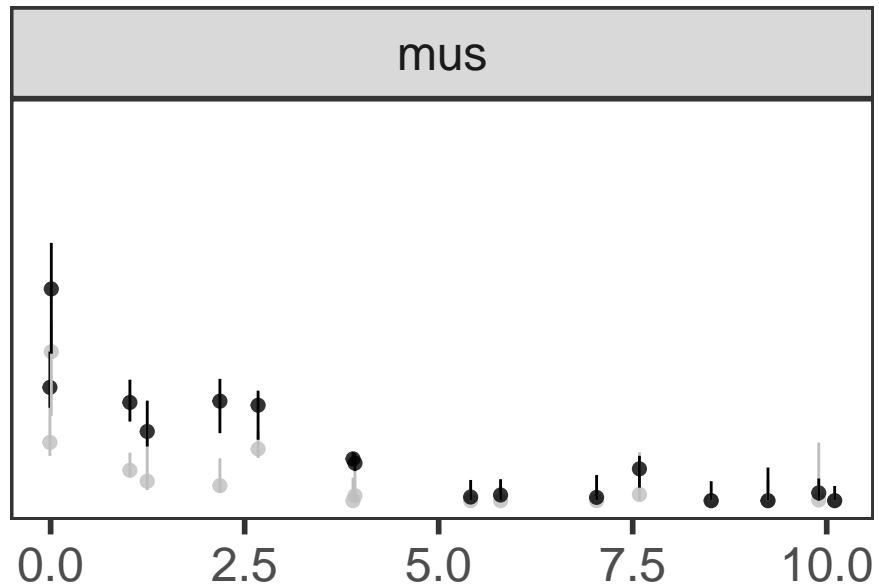

Tooth

lower  
upper

Supplement: Supplementary file 6 — Source Data [file 41467_2025_55826_MOESM6_ESM.zip › source data/Code_et_data_for_Fig/Code_and_data_for_fig3/fig3_panelD.pdf]

hamster

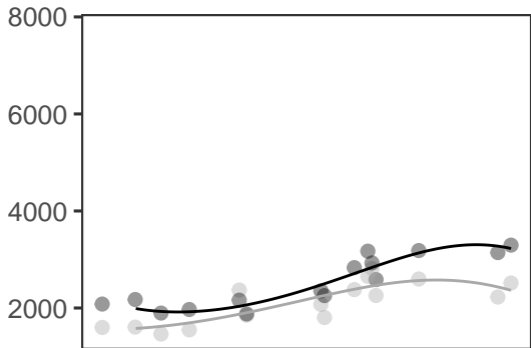

mouse

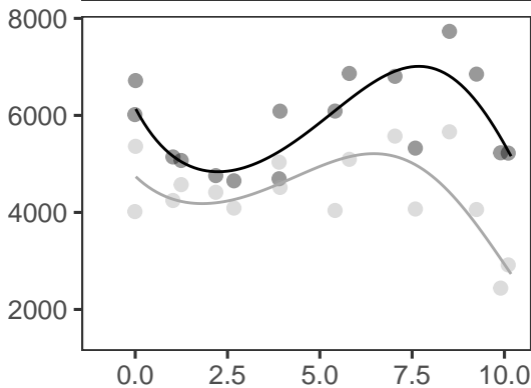

Supplement: Supplementary file 6 — Source Data [file 41467_2025_55826_MOESM6_ESM.zip › source data/Code_et_data_for_Fig/Code_and_data_for_fig4/Dlx1.pdf]

subset3

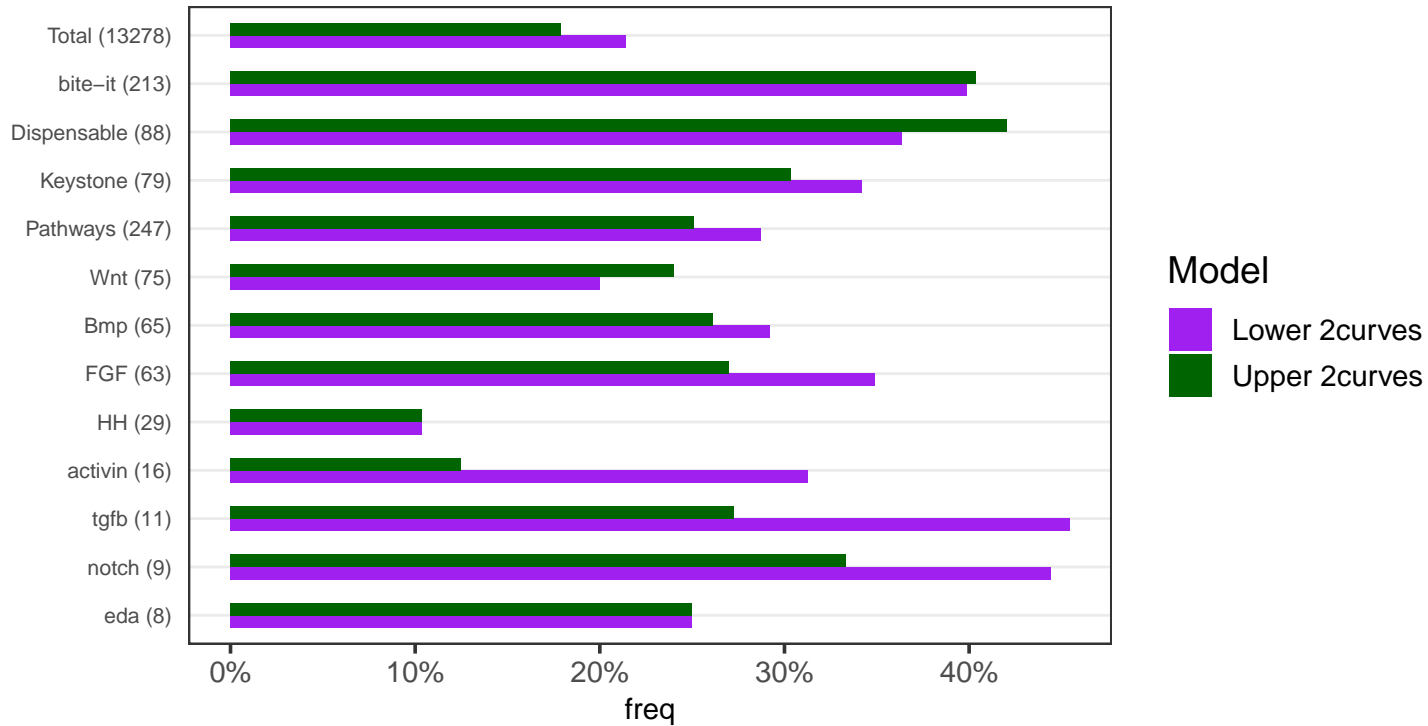

Supplement: Supplementary file 6 — Source Data [file 41467_2025_55826_MOESM6_ESM.zip › source data/Code_et_data_for_Fig/Code_and_data_for_fig4/fig4_panelE_left.pdf]

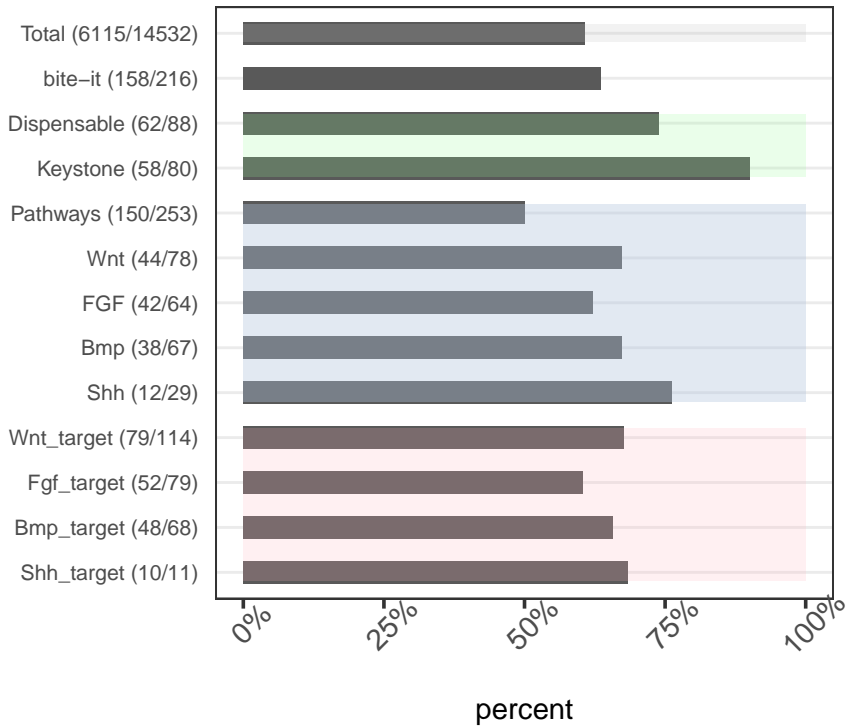

Supplement: Supplementary file 6 — Source Data [file 41467_2025_55826_MOESM6_ESM.zip › source data/Code_et_data_for_Fig/Code_and_data_for_fig4/fig4_panelA_right.pdf]

Bmp4

Expr. level (Base Mean)

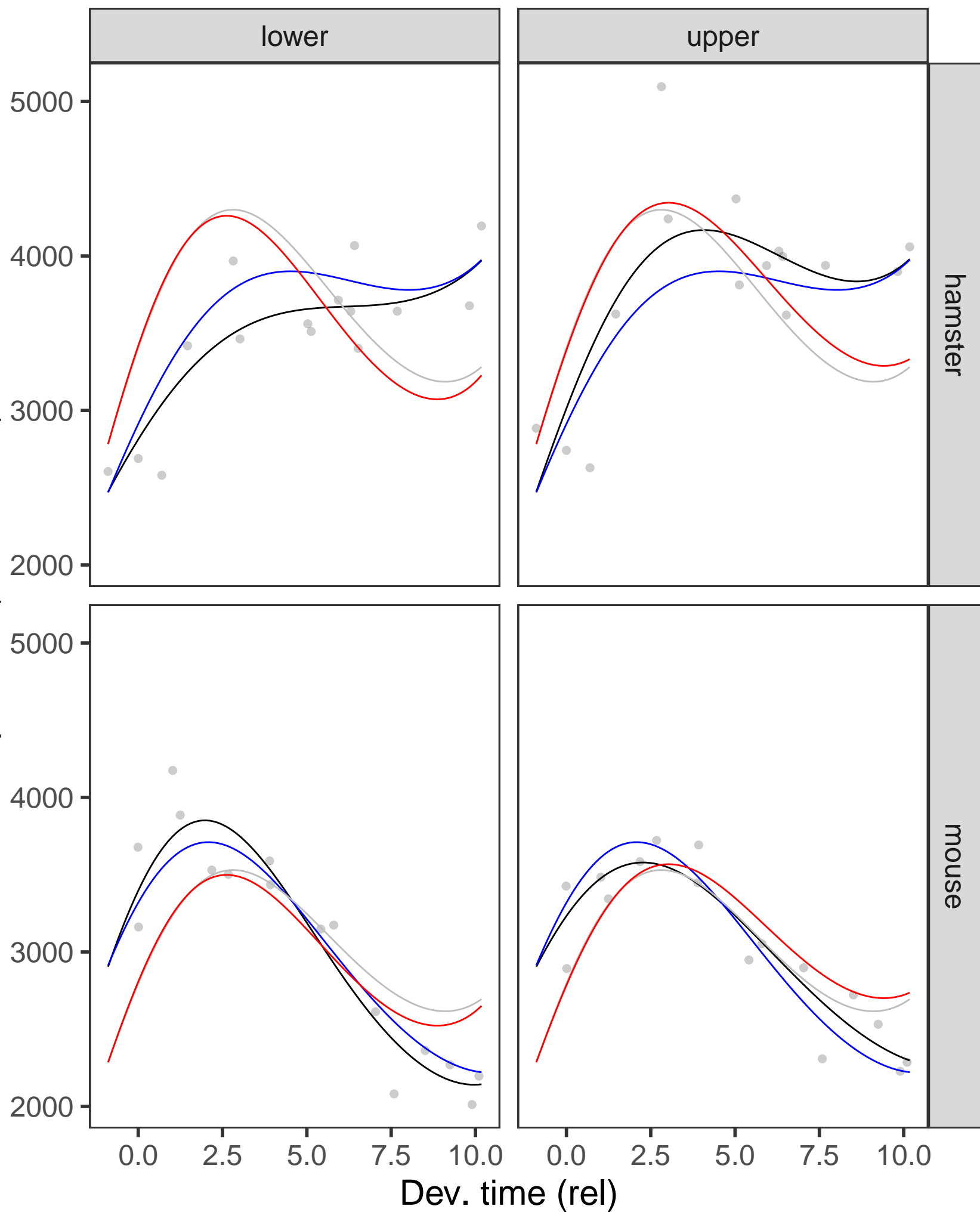

Model

- 4 teeth
- Hamster-mouse
- Simple
- Upper-lower

Supplement: Supplementary file 6 — Source Data [file 41467_2025_55826_MOESM6_ESM.zip › source data/Code_et_data_for_Fig/Code_and_data_for_fig4/fig4_panelA_left.pdf]

subset3

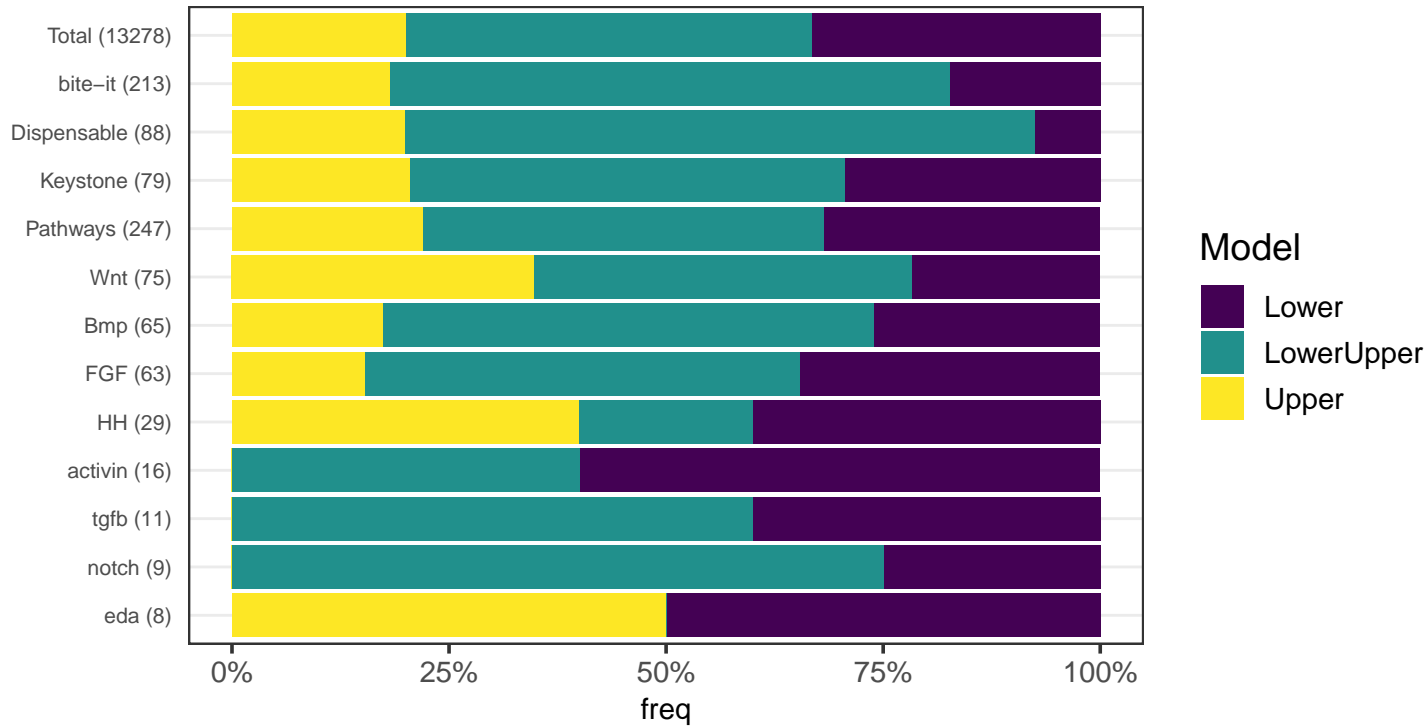

Supplement: Supplementary file 6 — Source Data [file 41467_2025_55826_MOESM6_ESM.zip › source data/Code_et_data_for_Fig/Code_and_data_for_fig4/fig4_panelE_right.pdf]

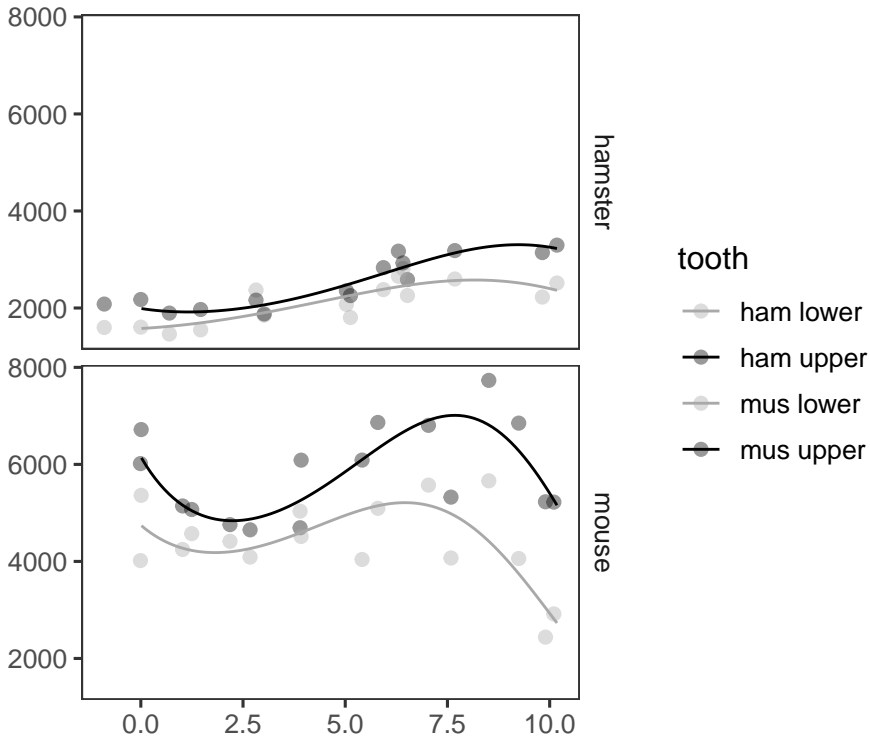

Supplement: Supplementary file 6 — Source Data [file 41467_2025_55826_MOESM6_ESM.zip › source data/Code_et_data_for_Fig/Code_and_data_for_fig4/fig4_panelD.pdf]

Bmp4 4vssp=0.097 4vsTooth=1e-20 Spvs1=6e-18 Tovs1=0.82

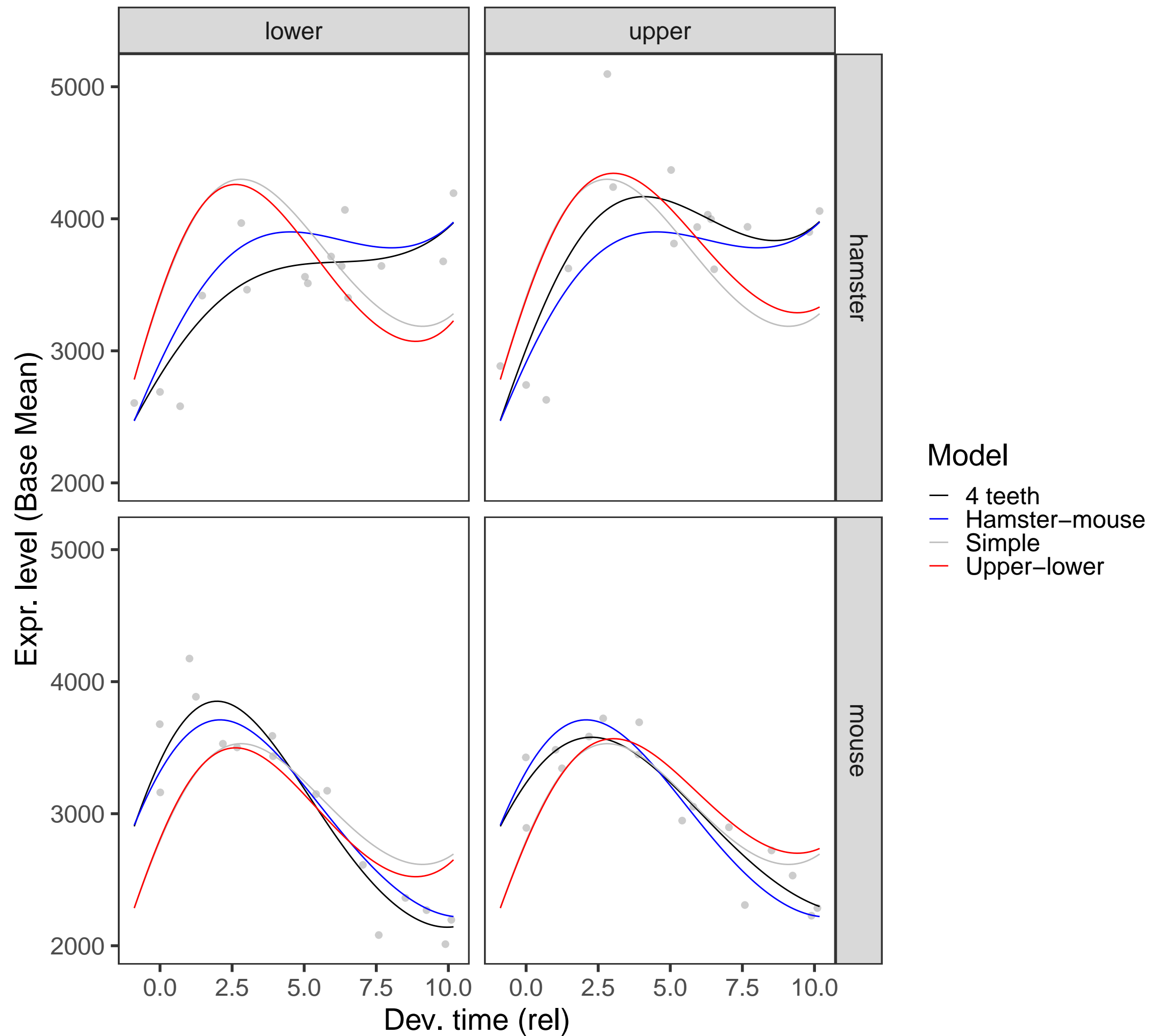

Supplement: Supplementary file 6 — Source Data [file 41467_2025_55826_MOESM6_ESM.zip › source data/Code_et_data_for_Fig/Code_and_data_for_fig4/Bmp4_fig4_panelA.pdf]

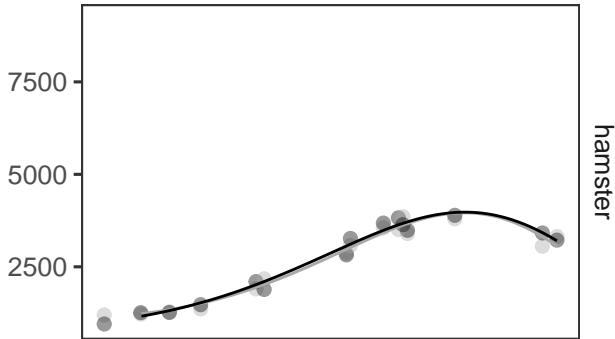

tooth

— ham lower

— ham upper

— mus lower

— mus upper

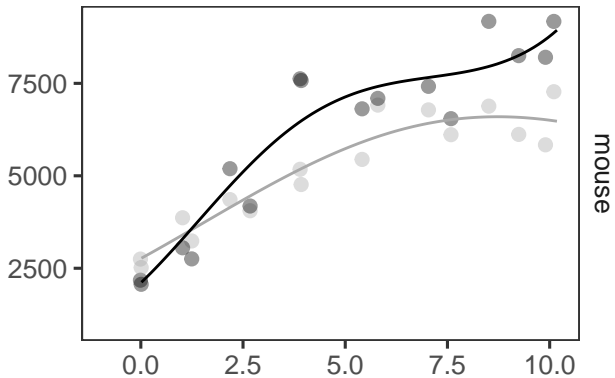

Supplement: Supplementary file 6 — Source Data [file 41467_2025_55826_MOESM6_ESM.zip › source data/Code_et_data_for_Fig/Code_and_data_for_fig4/fig4_panelC.pdf]

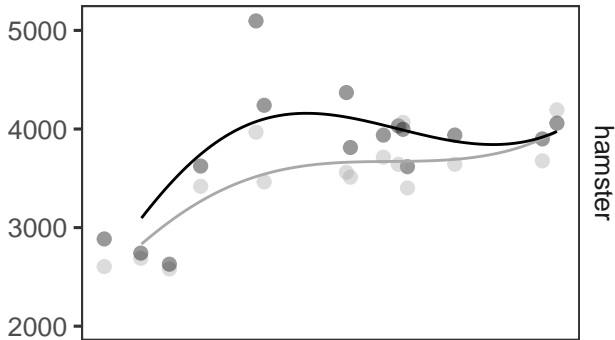

tooth

— ham lower

— ham upper

— mus lower

— mus upper

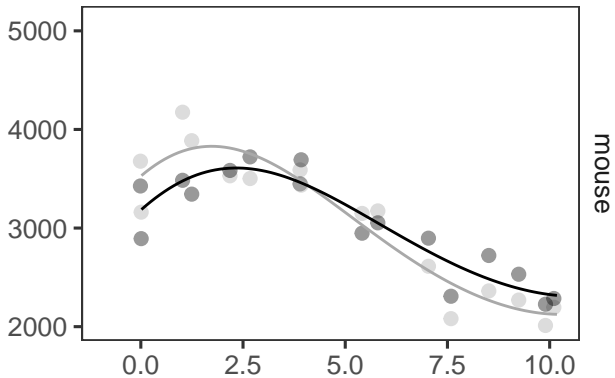

Supplement: Supplementary file 6 — Source Data [file 41467_2025_55826_MOESM6_ESM.zip › source data/Code_et_data_for_Fig/Code_and_data_for_fig4/fig4_panelB.pdf]

Qm

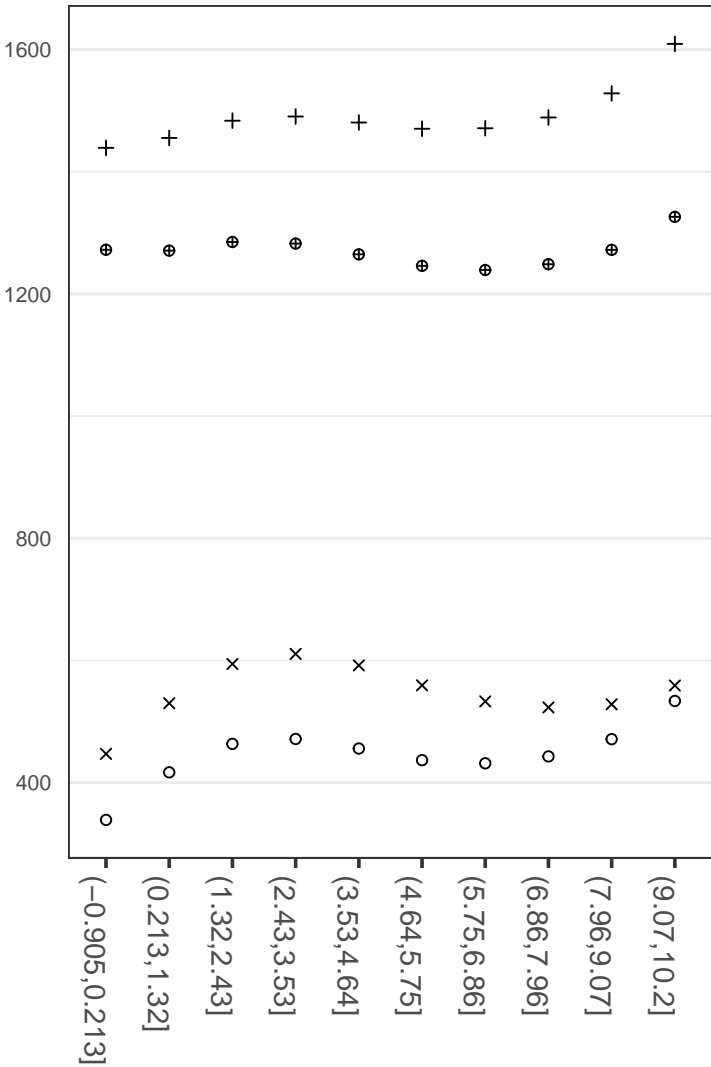

comparison

- ham
- ⊕ md
- × mus
- + mx

Supplement: Supplementary file 6 — Source Data [file 41467_2025_55826_MOESM6_ESM.zip › source data/Code_et_data_for_Fig/Code_and_data_for_fig2/fig2_panelD.pdf]

Axis1 46.9 %

Axis2 10.9 %

Dev. time

100

75

50

25

0

Tooth

● lower hamster

▲ lower mus

■ upper hamster

◆ upper mus

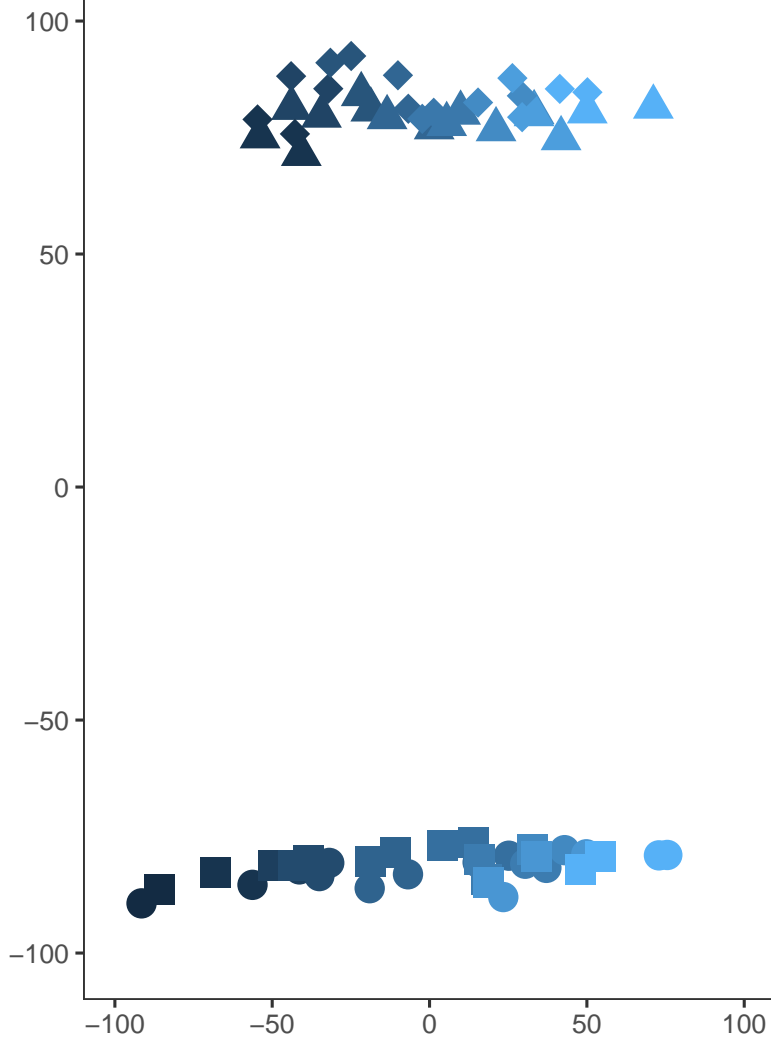

Supplement: Supplementary file 6 — Source Data [file 41467_2025_55826_MOESM6_ESM.zip › source data/Code_et_data_for_Fig/Code_and_data_for_fig2/fig2_panelB.pdf]

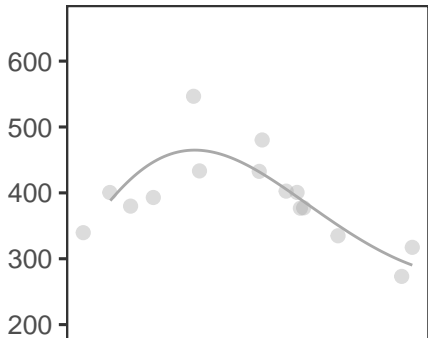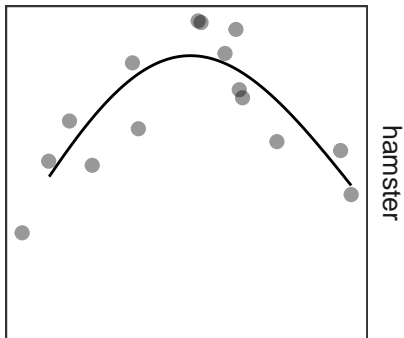

tooth

— ham lower

— ham upper

— mus lower

— mus upper

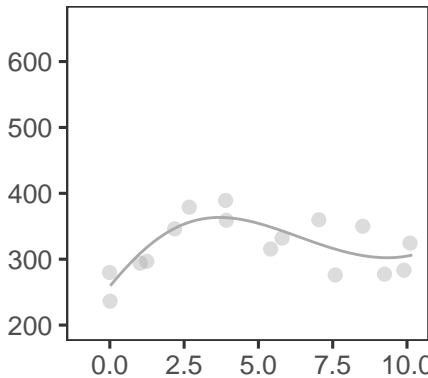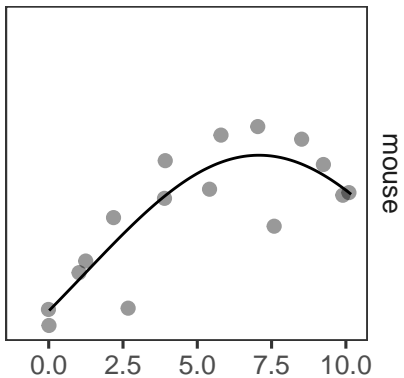

Supplement: Supplementary file 6 — Source Data [file 41467_2025_55826_MOESM6_ESM.zip › source data/Code_et_data_for_Fig/Code_and_data_for_fig2/fig2_panelC.pdf]

species

mus

ham

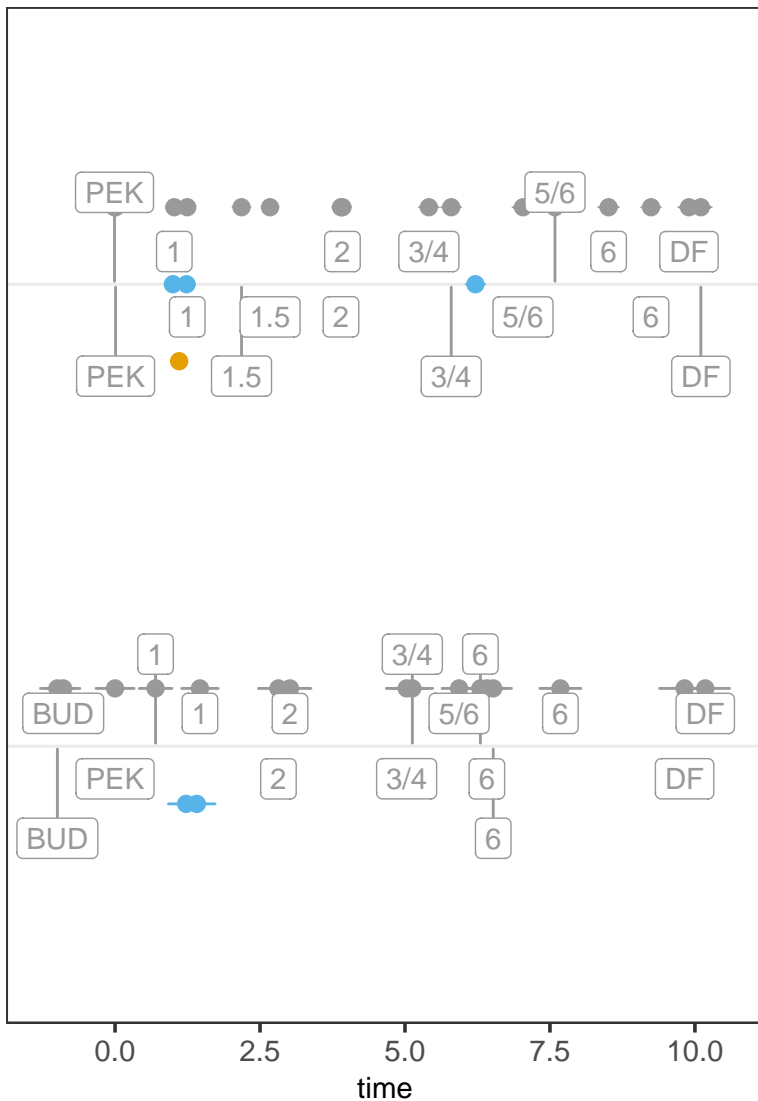

type

a

BL

a

EpiMes

a

Whole

Supplement: Supplementary file 6 — Source Data [file 41467_2025_55826_MOESM6_ESM.zip › source data/Code_et_data_for_Fig/Code_and_data_for_fig2/fig2_panelA.pdf]

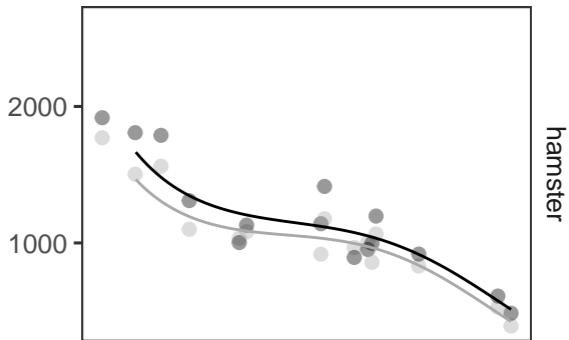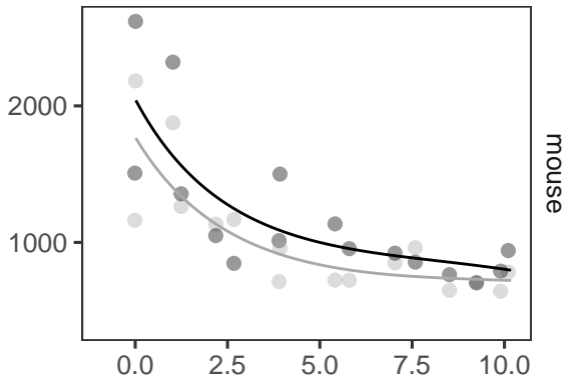

Supplement: Supplementary file 6 — Source Data [file 41467_2025_55826_MOESM6_ESM.zip › source data/Code_et_data_for_Fig/Code_and_data_for_Sfig7/Inhba.pdf]

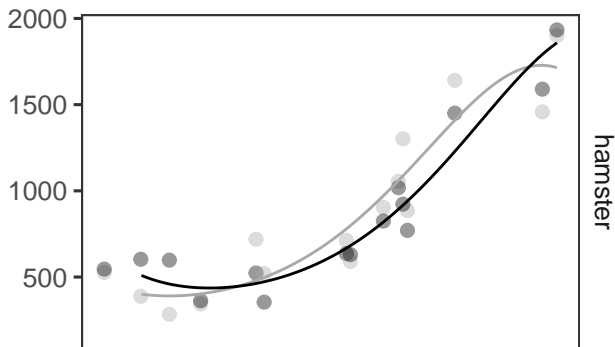

tooth

— ham lower

— ham upper

— mus lower

— mus upper

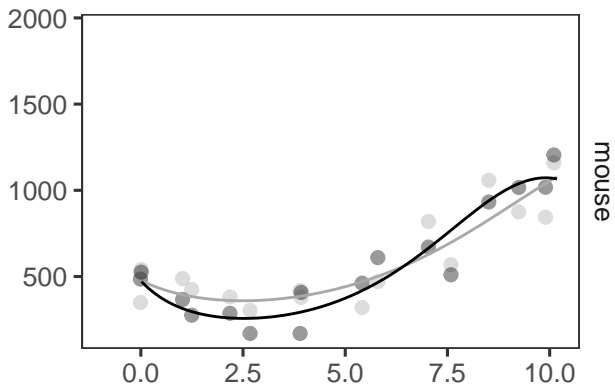

Supplement: Supplementary file 6 — Source Data [file 41467_2025_55826_MOESM6_ESM.zip › source data/Code_et_data_for_Fig/Code_and_data_for_Sfig7/figS7_panelA.pdf]

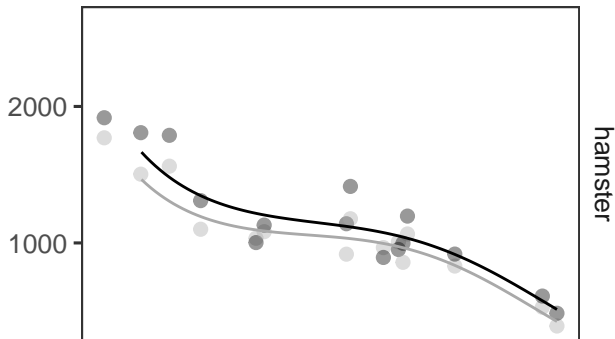

tooth

— ham lower

— ham upper

— mus lower

— mus upper

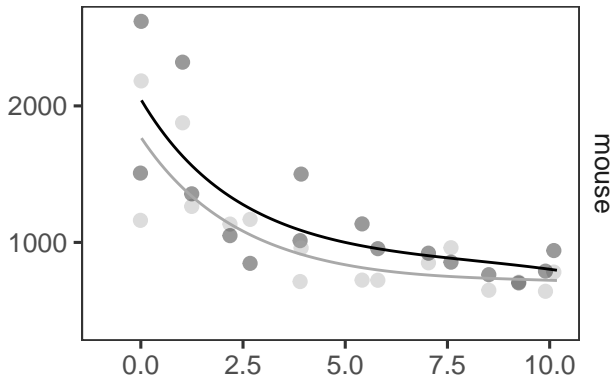

Supplement: Supplementary file 6 — Source Data [file 41467_2025_55826_MOESM6_ESM.zip › source data/Code_et_data_for_Fig/Code_and_data_for_Sfig7/figS7_panelB.pdf]

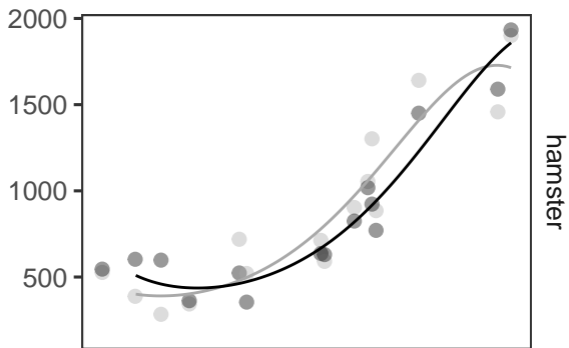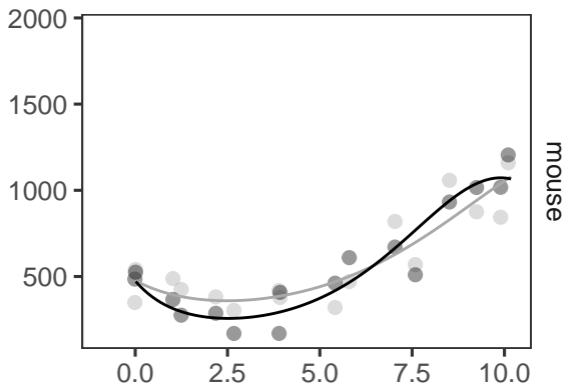

Supplement: Supplementary file 6 — Source Data [file 41467_2025_55826_MOESM6_ESM.zip › source data/Code_et_data_for_Fig/Code_and_data_for_Sfig7/Fgf3.pdf]

Weight and stage model MOUSE

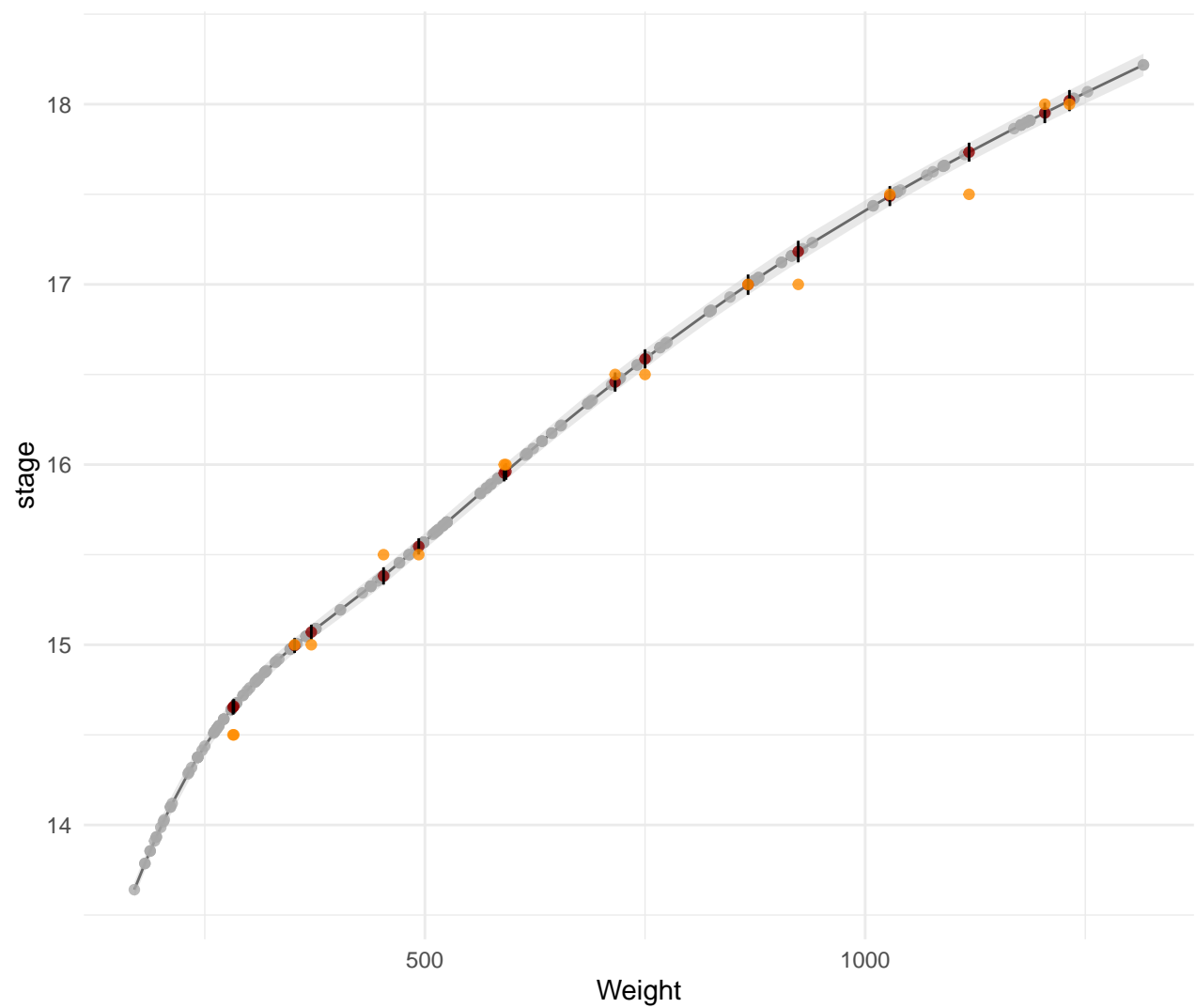

Supplement: Supplementary file 6 — Source Data [file 41467_2025_55826_MOESM6_ESM.zip › source data/Code_et_data_for_Fig/Code_and_data_for_Sfig1/FigS1_PanelC_left.pdf]

Weight and stage model MOUSE

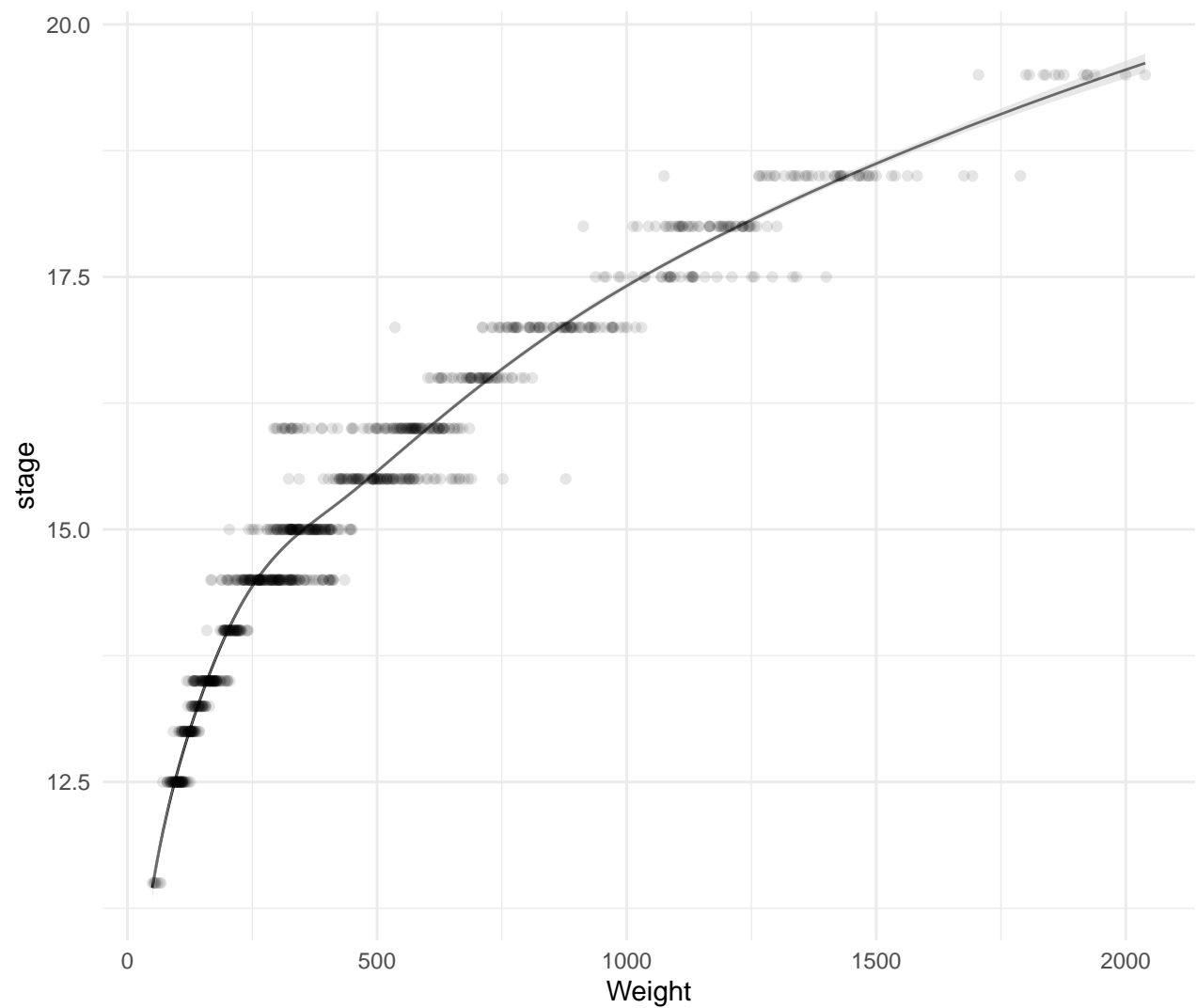

Supplement: Supplementary file 6 — Source Data [file 41467_2025_55826_MOESM6_ESM.zip › source data/Code_et_data_for_Fig/Code_and_data_for_Sfig1/FigS1_PanelB_left.pdf]

Weight and stage model HAMSTER

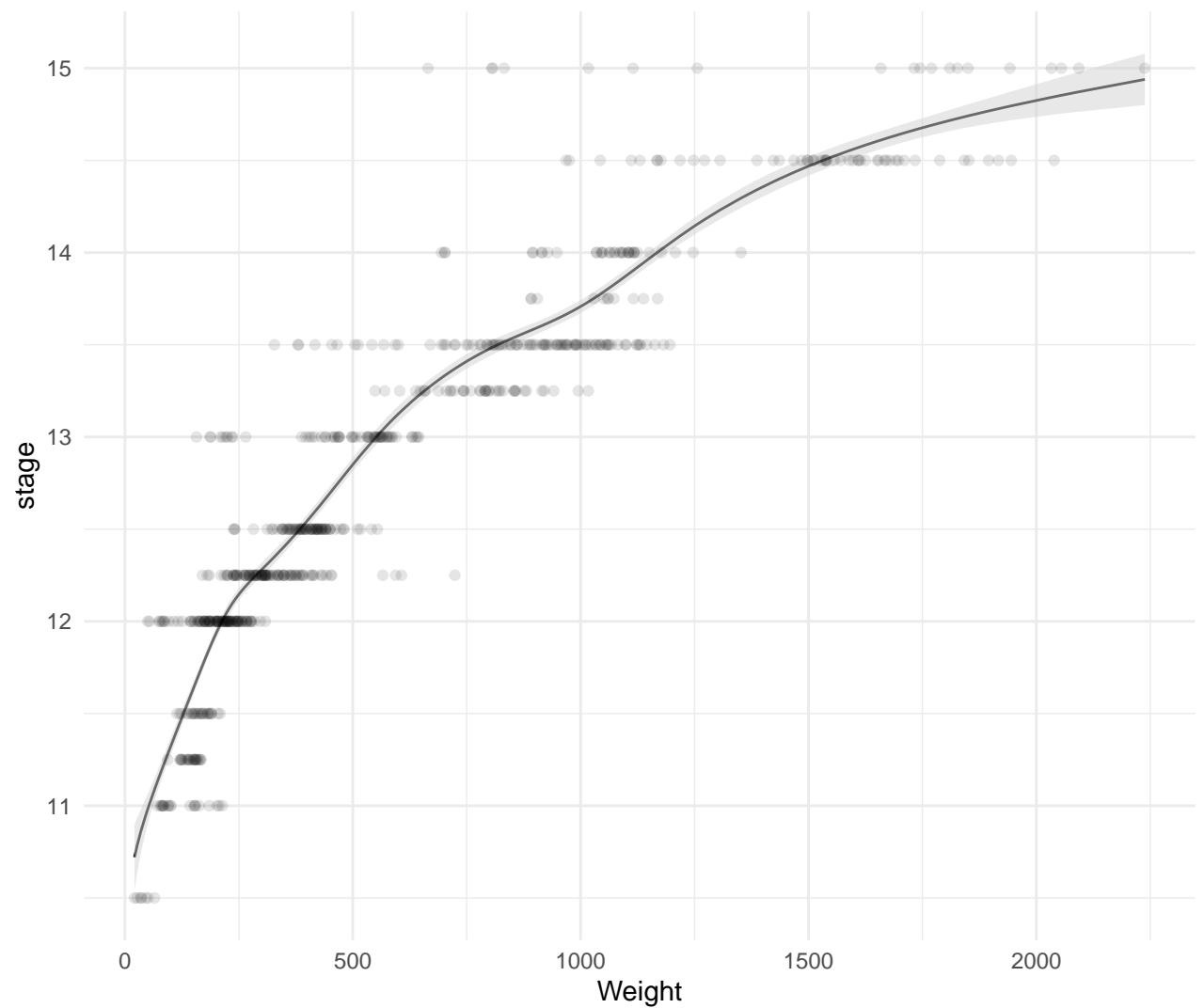

Supplement: Supplementary file 6 — Source Data [file 41467_2025_55826_MOESM6_ESM.zip › source data/Code_et_data_for_Fig/Code_and_data_for_Sfig1/FigS1_PanelB_right.pdf]

Weight and stage model HAMSTER

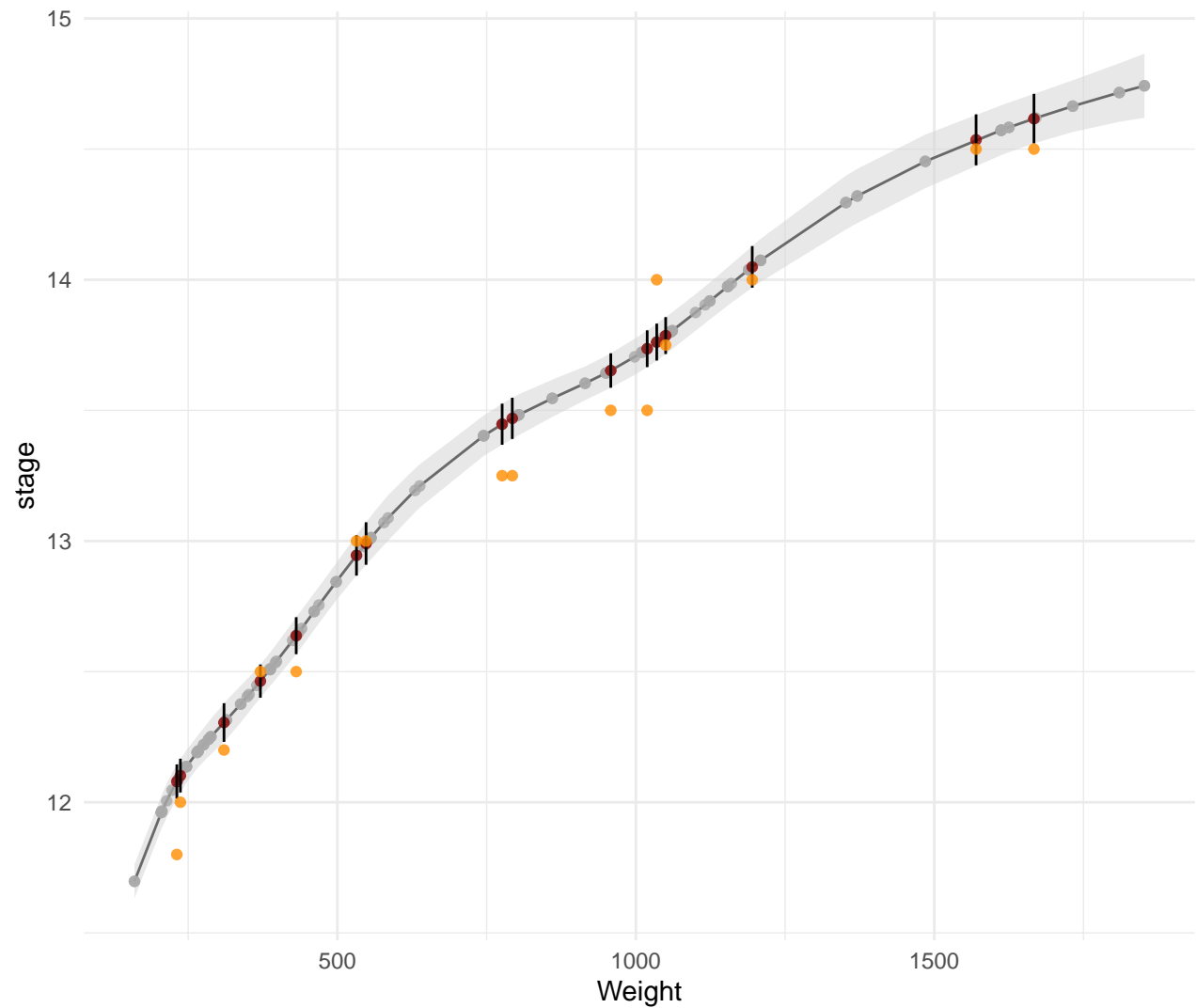

Supplement: Supplementary file 6 — Source Data [file 41467_2025_55826_MOESM6_ESM.zip › source data/Code_et_data_for_Fig/Code_and_data_for_Sfig1/FigS1_PanelC_right.pdf]

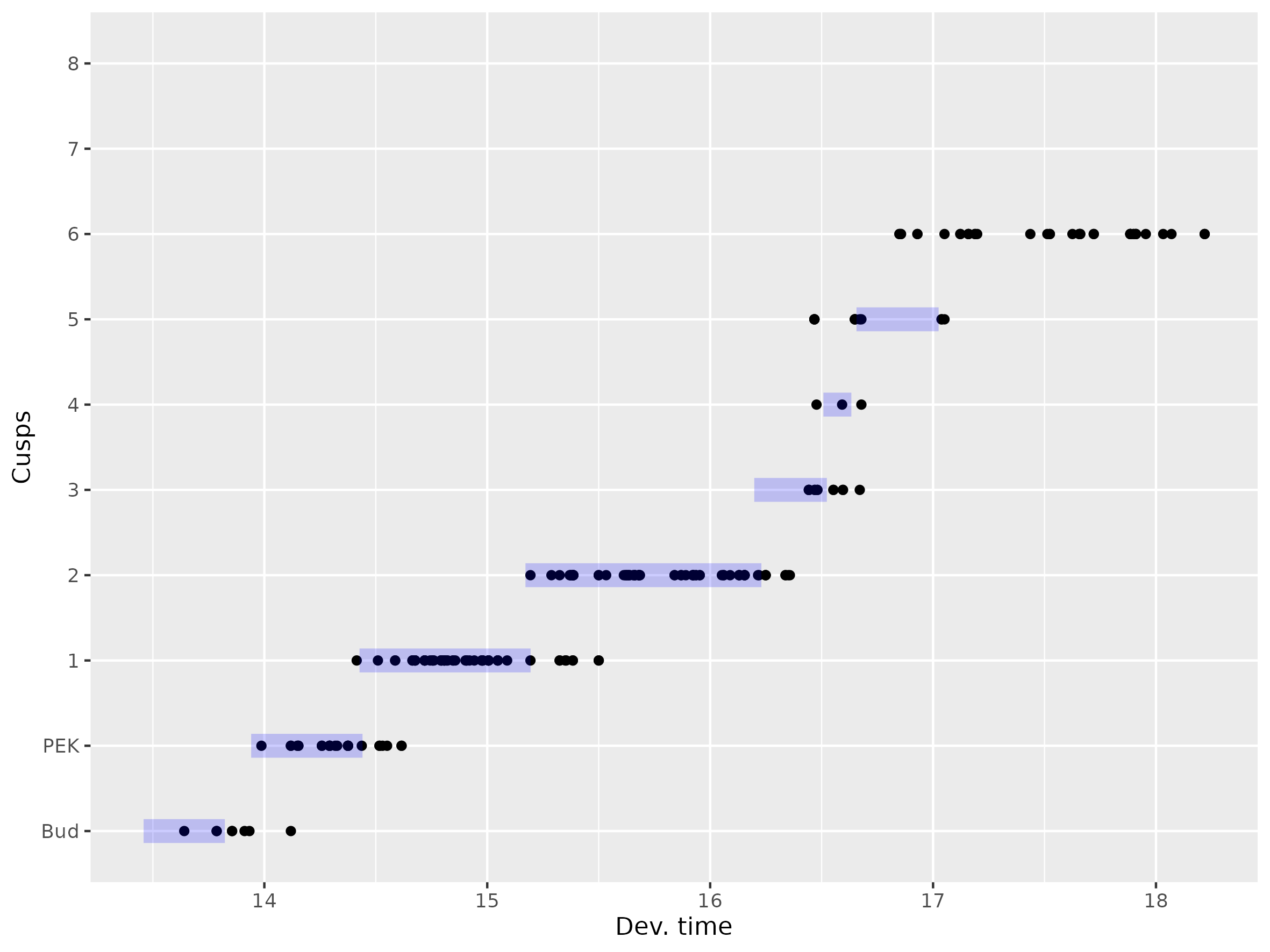

Supplement: Supplementary file 6 — Source Data [file 41467_2025_55826_MOESM6_ESM.zip › source data/Code_et_data_for_Fig/Code_and_data_for_fig1/Mus Lower.png]

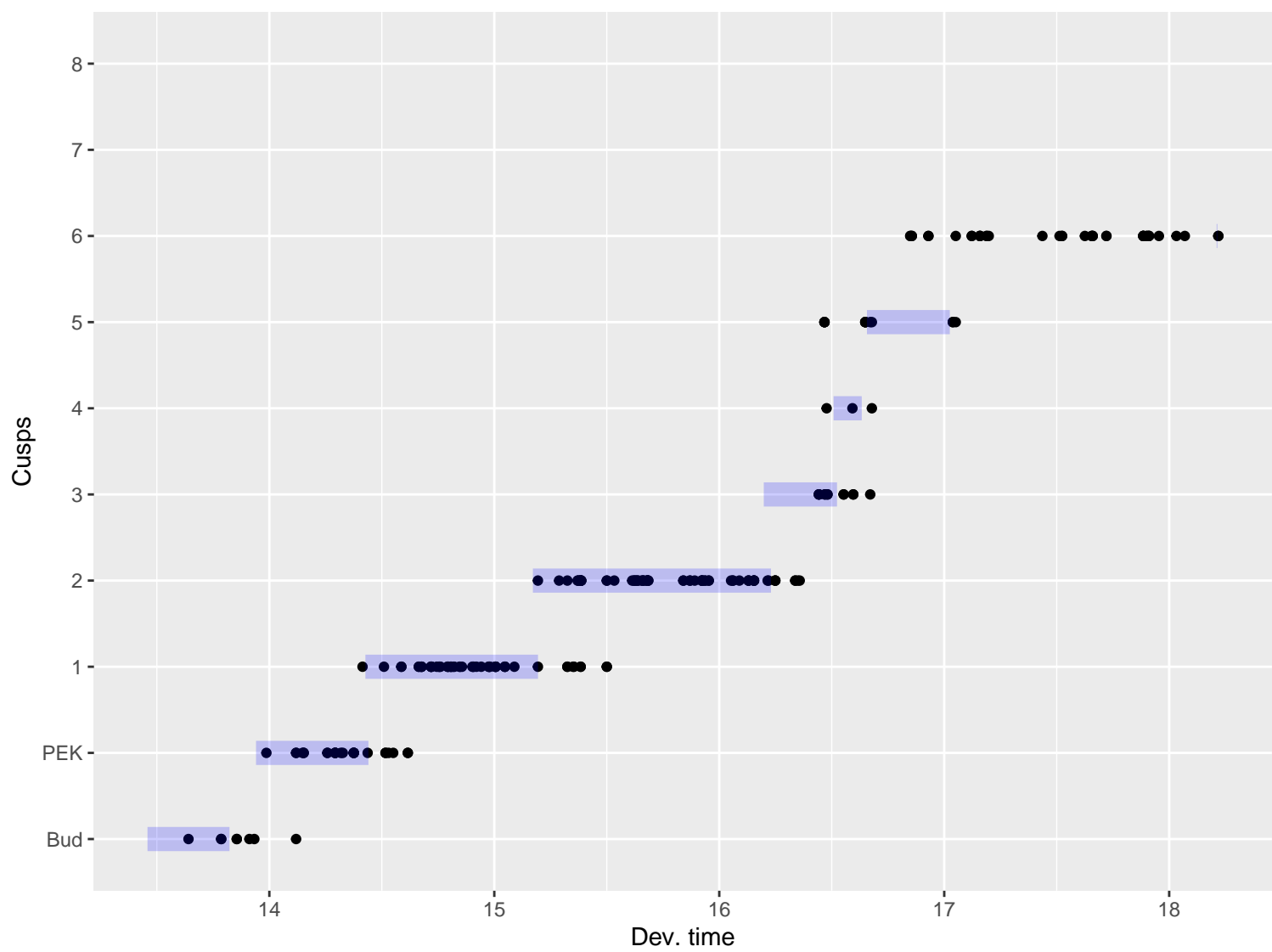

Supplement: Supplementary file 6 — Source Data [file 41467_2025_55826_MOESM6_ESM.zip › source data/Code_et_data_for_Fig/Code_and_data_for_fig1/Mus Lower.pdf]

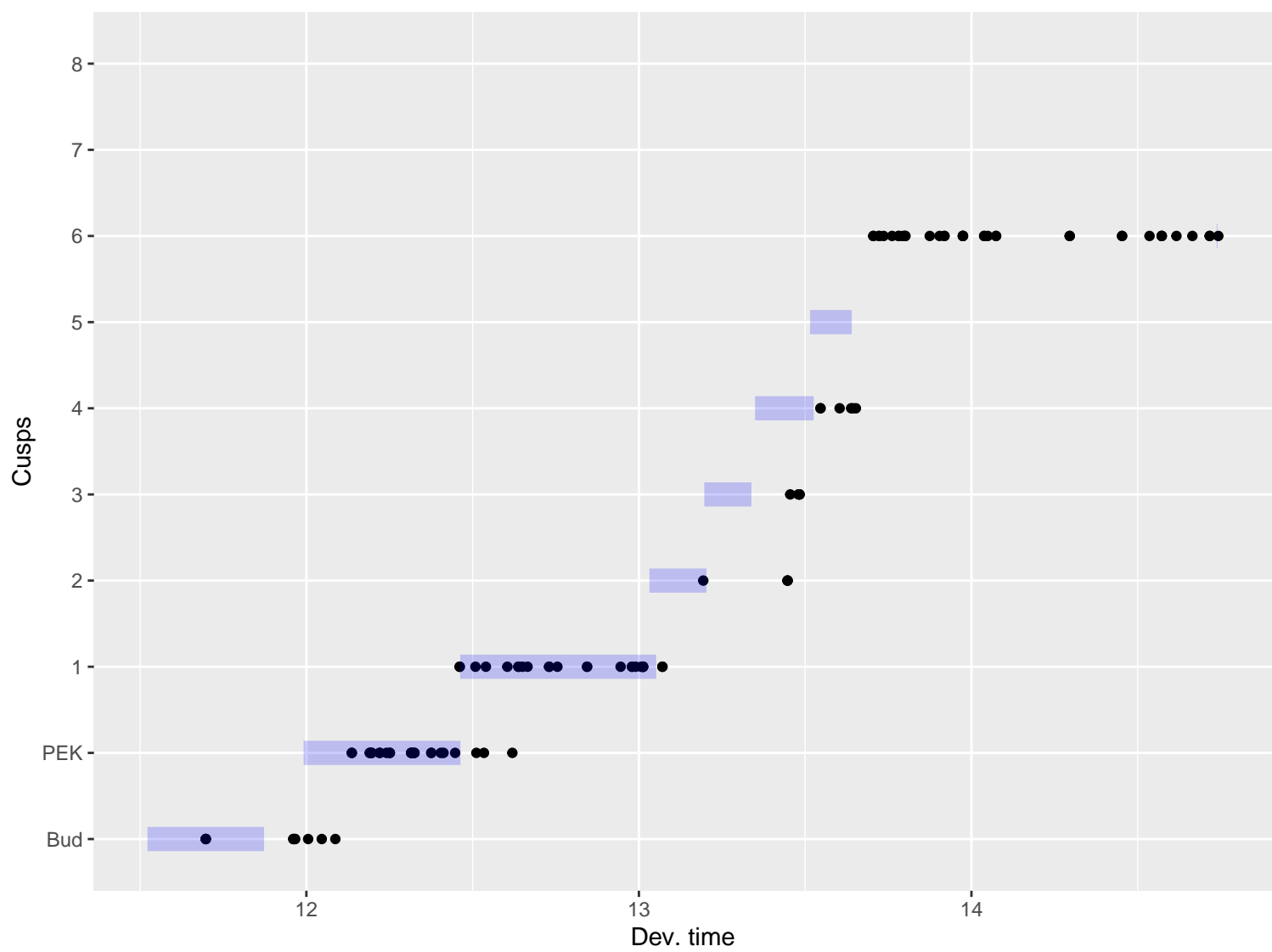

Supplement: Supplementary file 6 — Source Data [file 41467_2025_55826_MOESM6_ESM.zip › source data/Code_et_data_for_Fig/Code_and_data_for_fig1/Ham Upper.pdf]

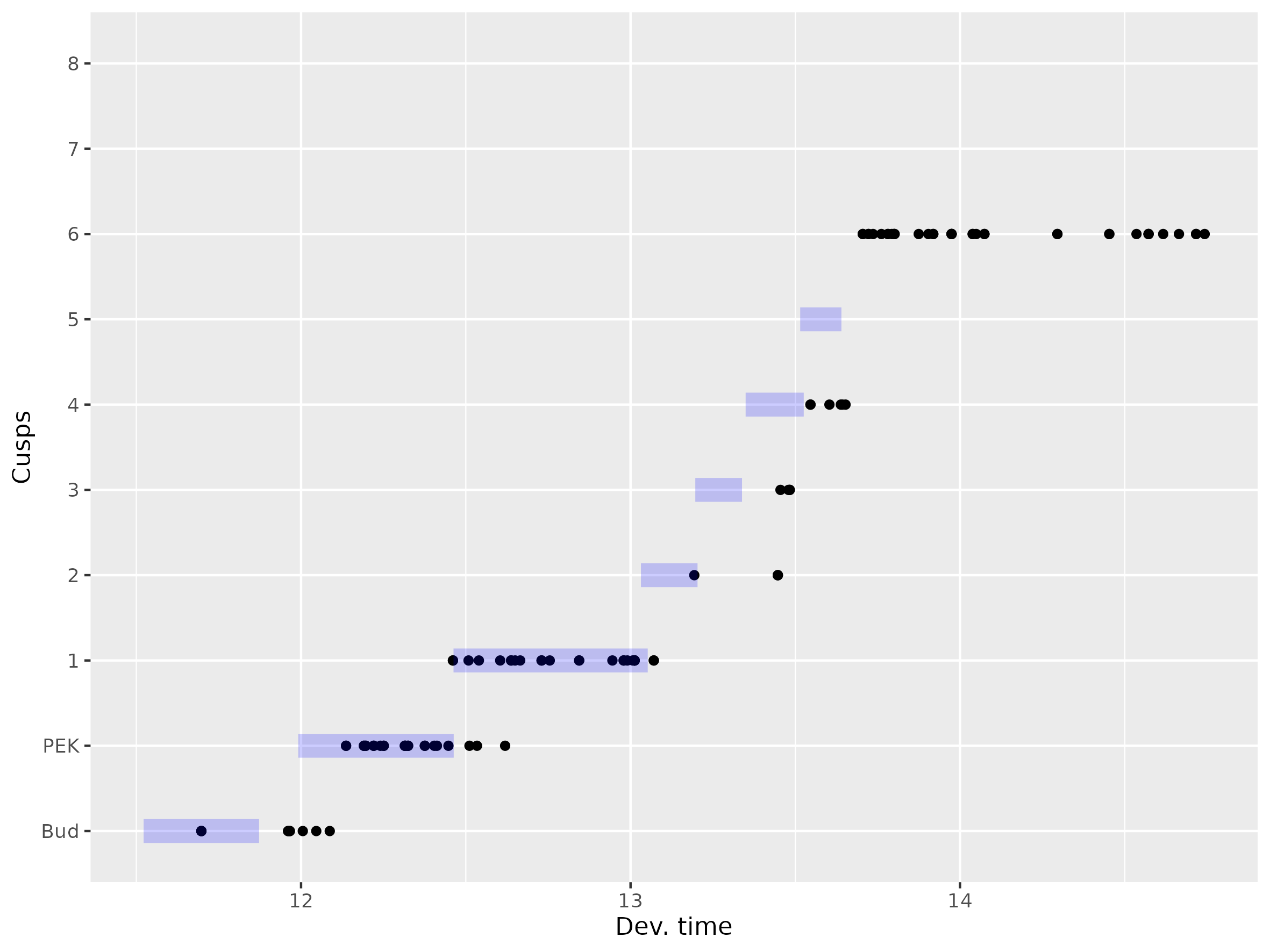

Supplement: Supplementary file 6 — Source Data [file 41467_2025_55826_MOESM6_ESM.zip › source data/Code_et_data_for_Fig/Code_and_data_for_fig1/Ham Upper.png]

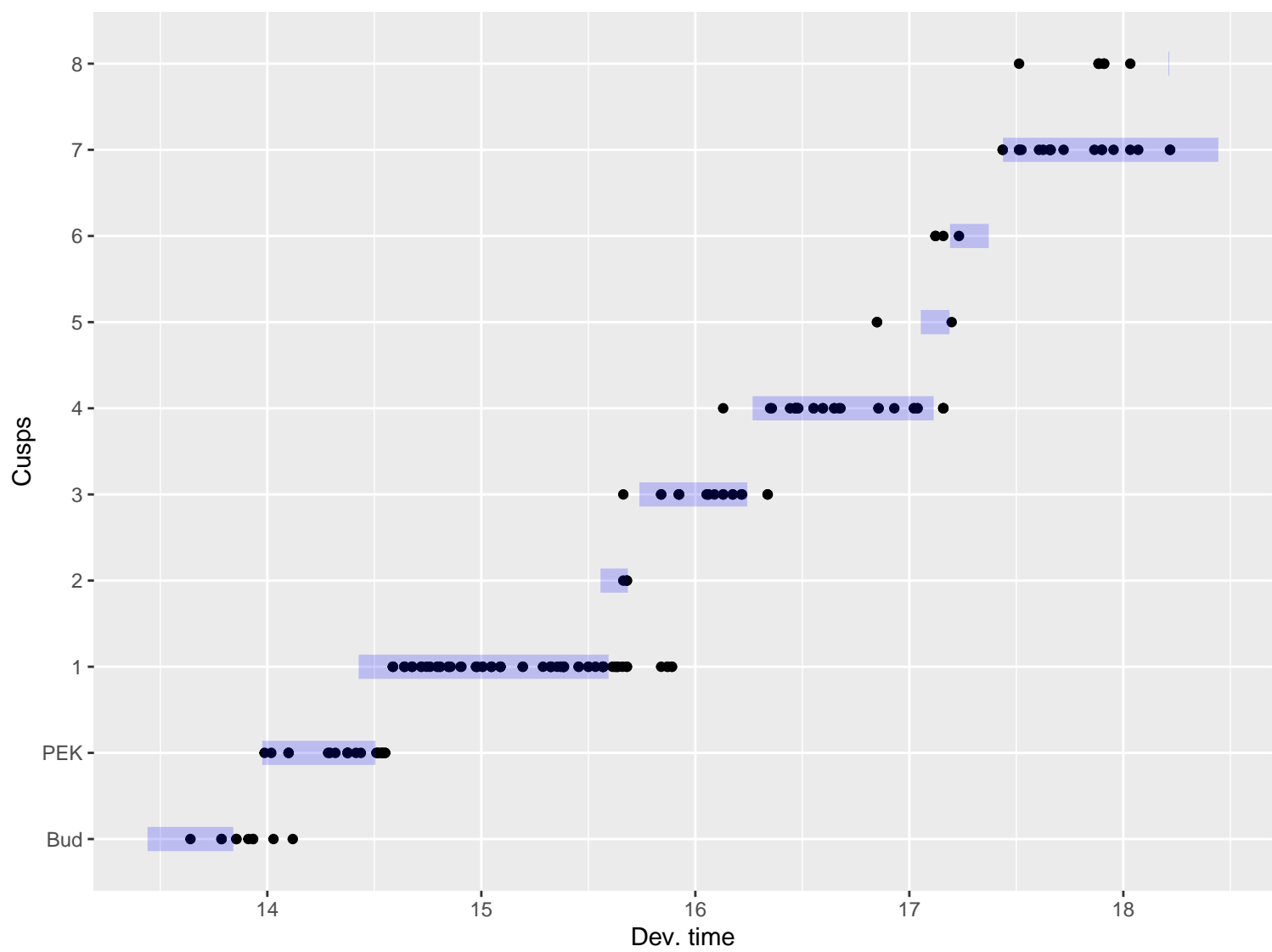

Supplement: Supplementary file 6 — Source Data [file 41467_2025_55826_MOESM6_ESM.zip › source data/Code_et_data_for_Fig/Code_and_data_for_fig1/Mus Upper.pdf]

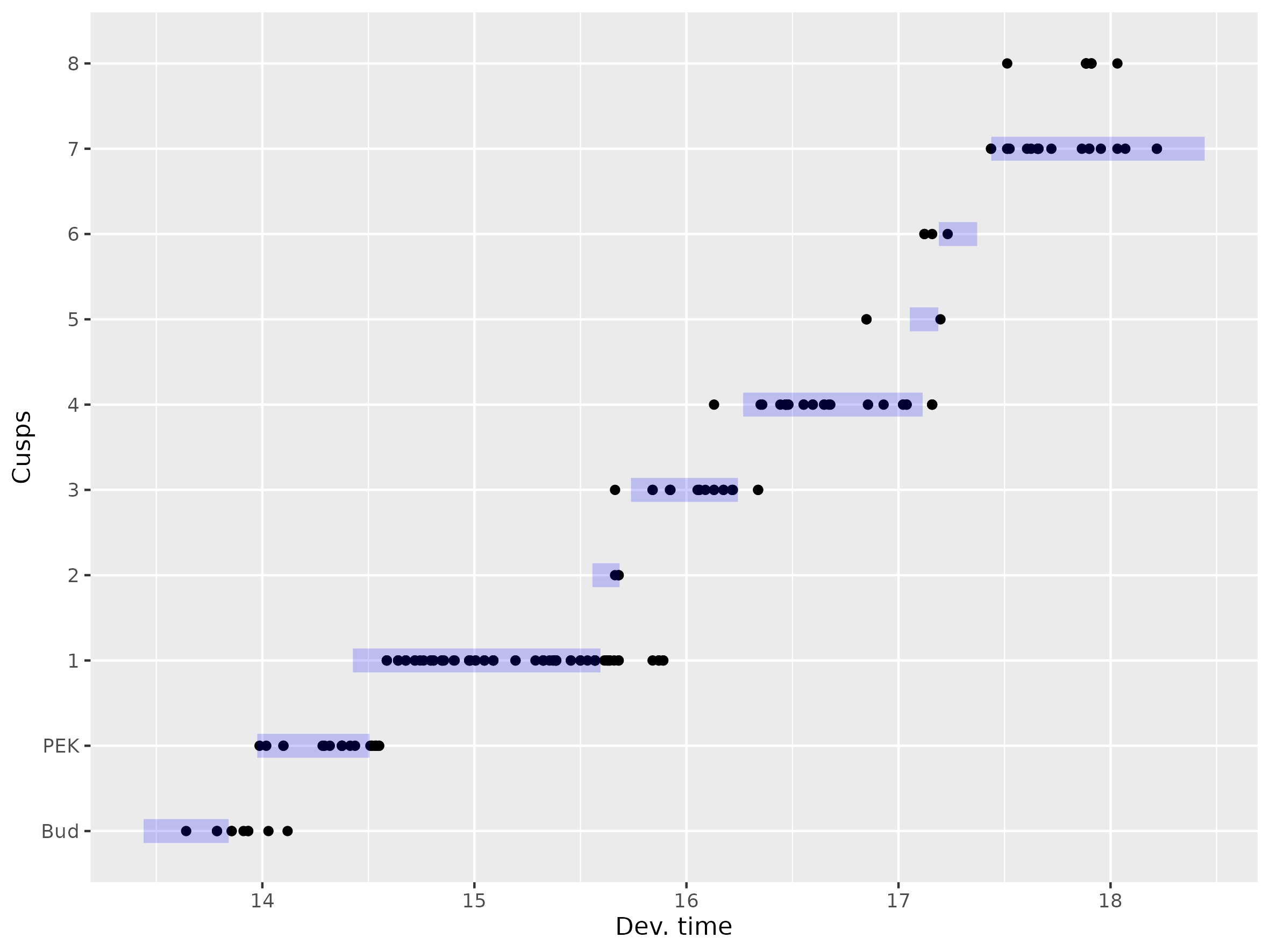

Supplement: Supplementary file 6 — Source Data [file 41467_2025_55826_MOESM6_ESM.zip › source data/Code_et_data_for_Fig/Code_and_data_for_fig1/Mus Upper.png]

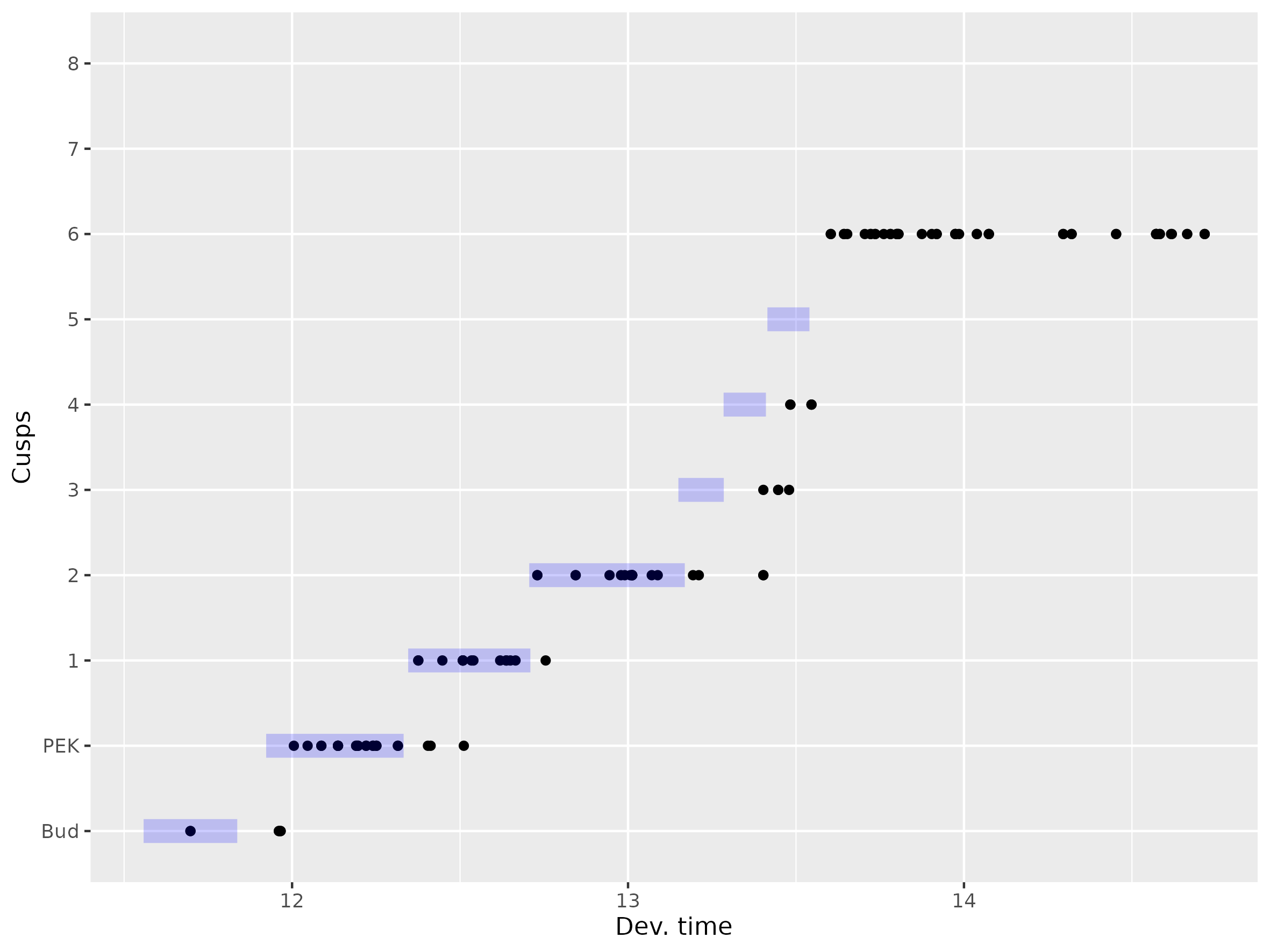

Supplement: Supplementary file 6 — Source Data [file 41467_2025_55826_MOESM6_ESM.zip › source data/Code_et_data_for_Fig/Code_and_data_for_fig1/Ham Lower.png]

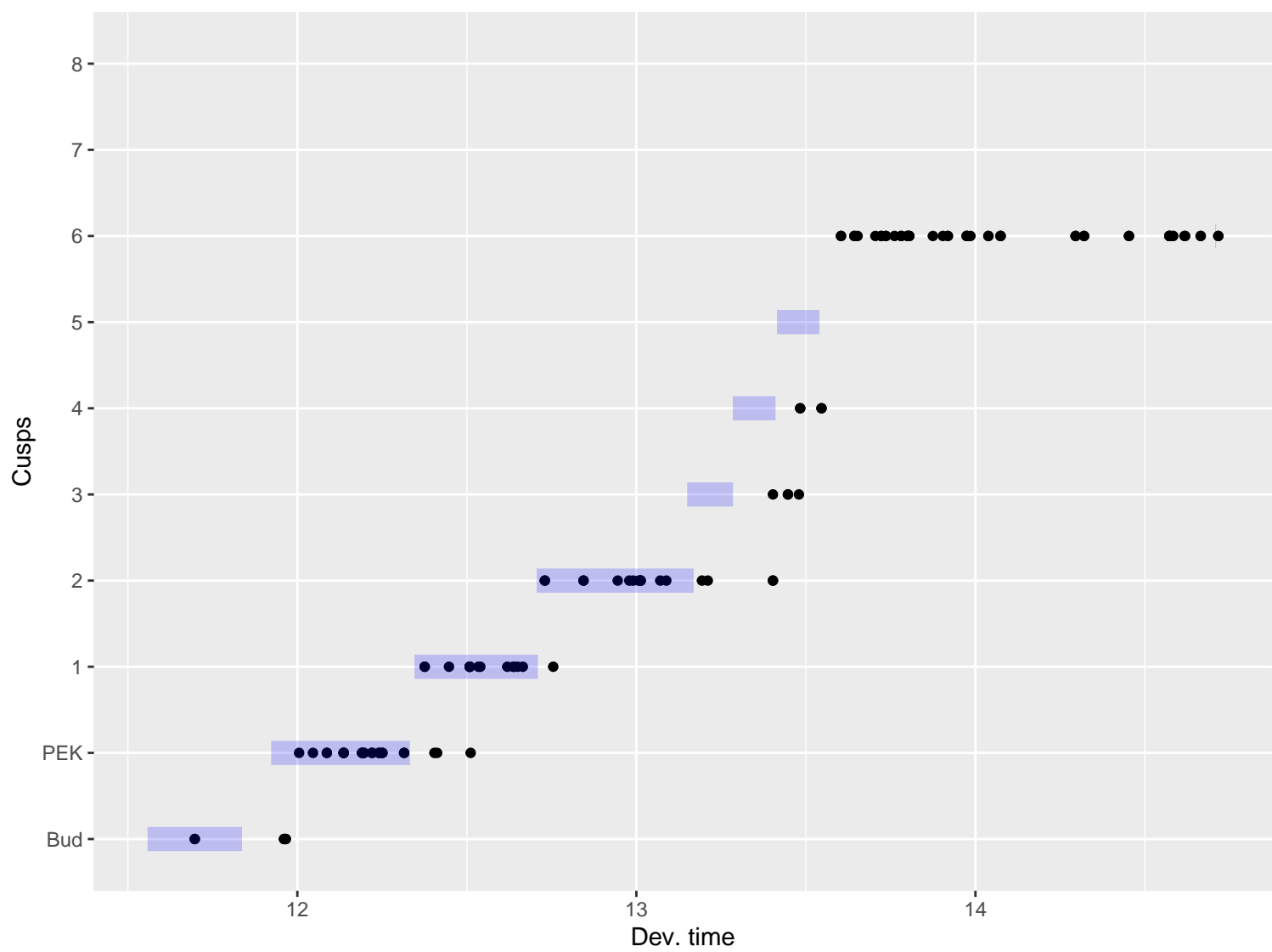

Supplement: Supplementary file 6 — Source Data [file 41467_2025_55826_MOESM6_ESM.zip › source data/Code_et_data_for_Fig/Code_and_data_for_fig1/Ham Lower.pdf]

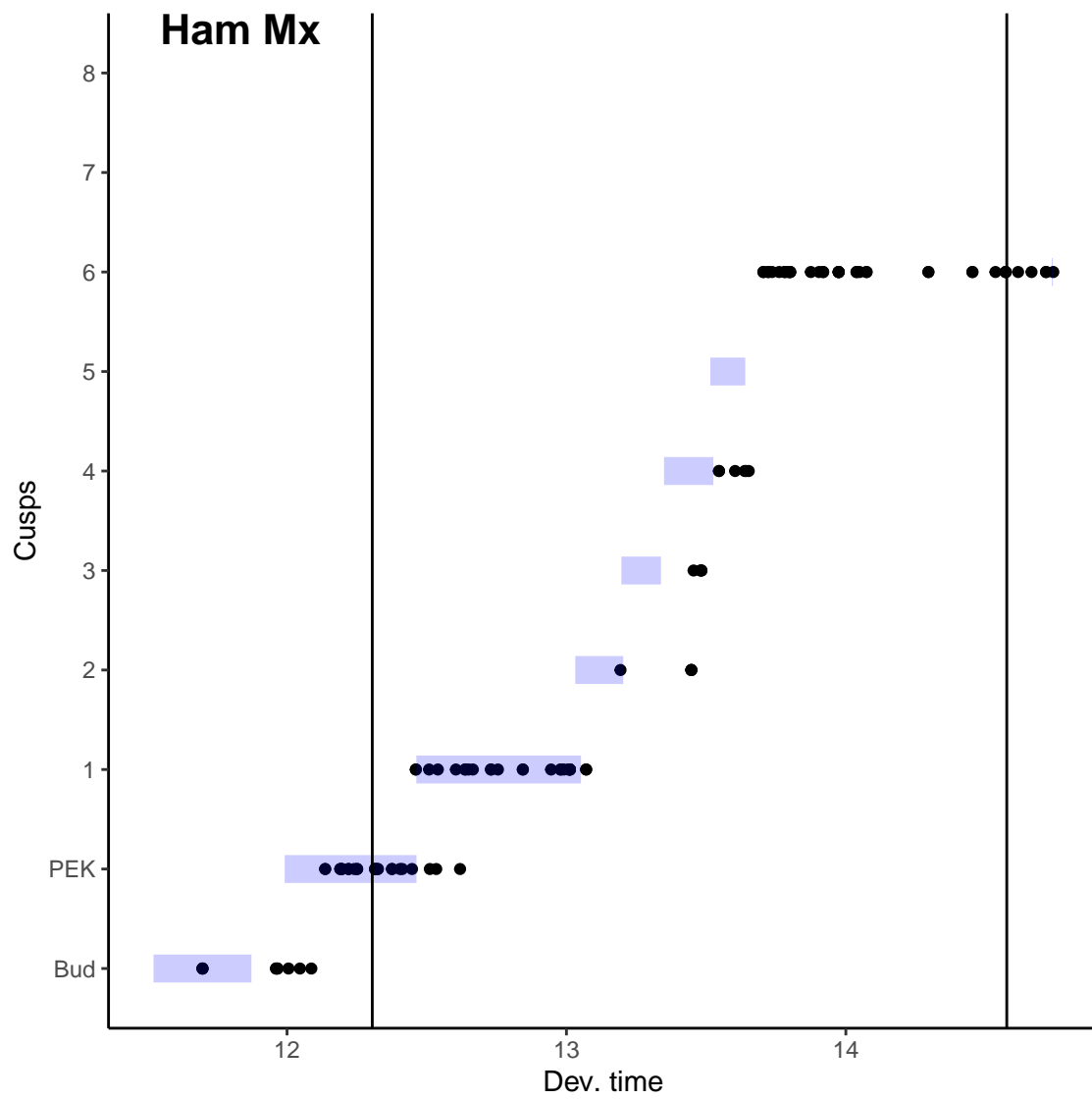

Supplement: Supplementary file 6 — Source Data [file 41467_2025_55826_MOESM6_ESM.zip › source data/Code_et_data_for_Fig/Code_and_data_for_fig1/fig1_panelB.pdf]

Shh

Expr. level (Base Mean)

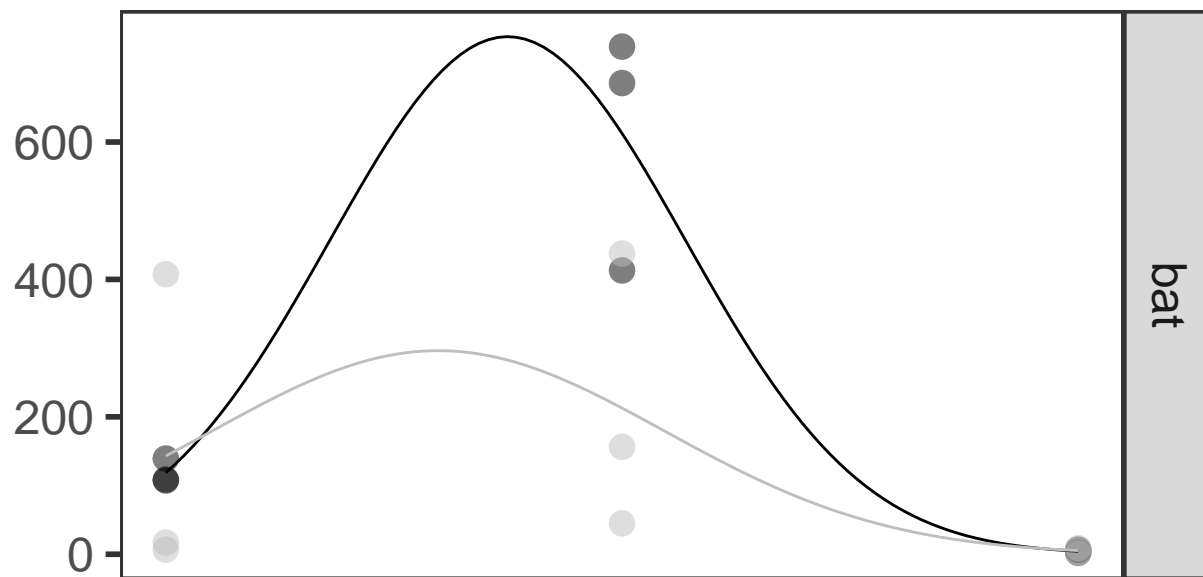

bat

Limb

● fore-limb  
● hind-limb

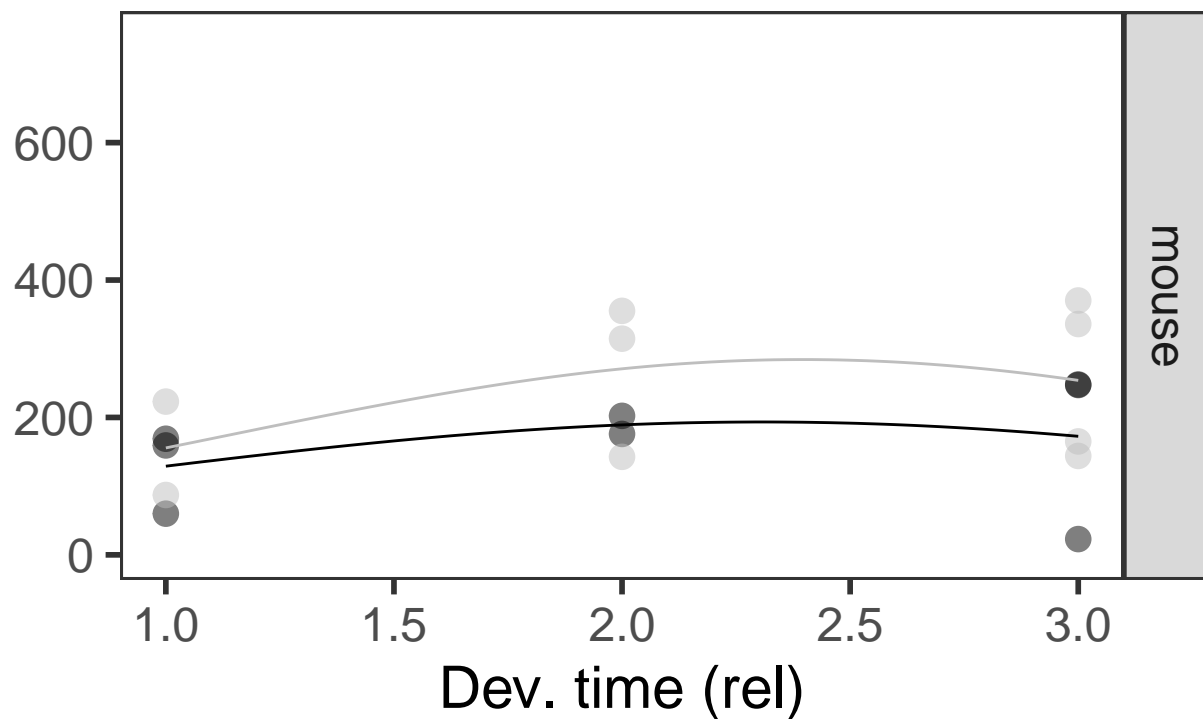

mouse

Supplement: Supplementary file 6 — Source Data [file 41467_2025_55826_MOESM6_ESM.zip › source data/Code_et_data_for_Fig/Code_and_data_for_fig6/Shh_fig6_panelBD.pdf]

Grem1

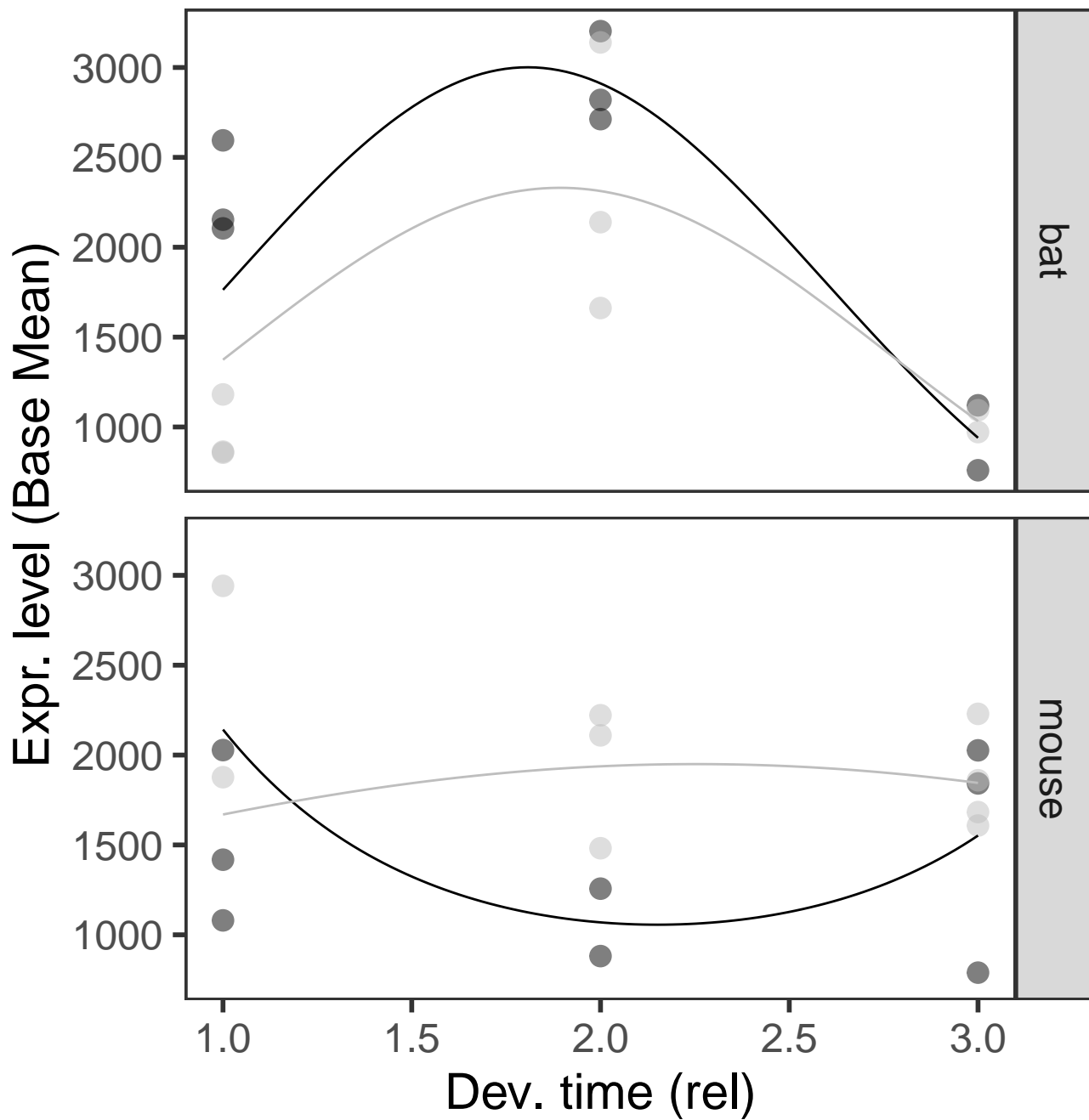

Supplement: Supplementary file 6 — Source Data [file 41467_2025_55826_MOESM6_ESM.zip › source data/Code_et_data_for_Fig/Code_and_data_for_fig6/fig6_panelD.pdf]

Grem1

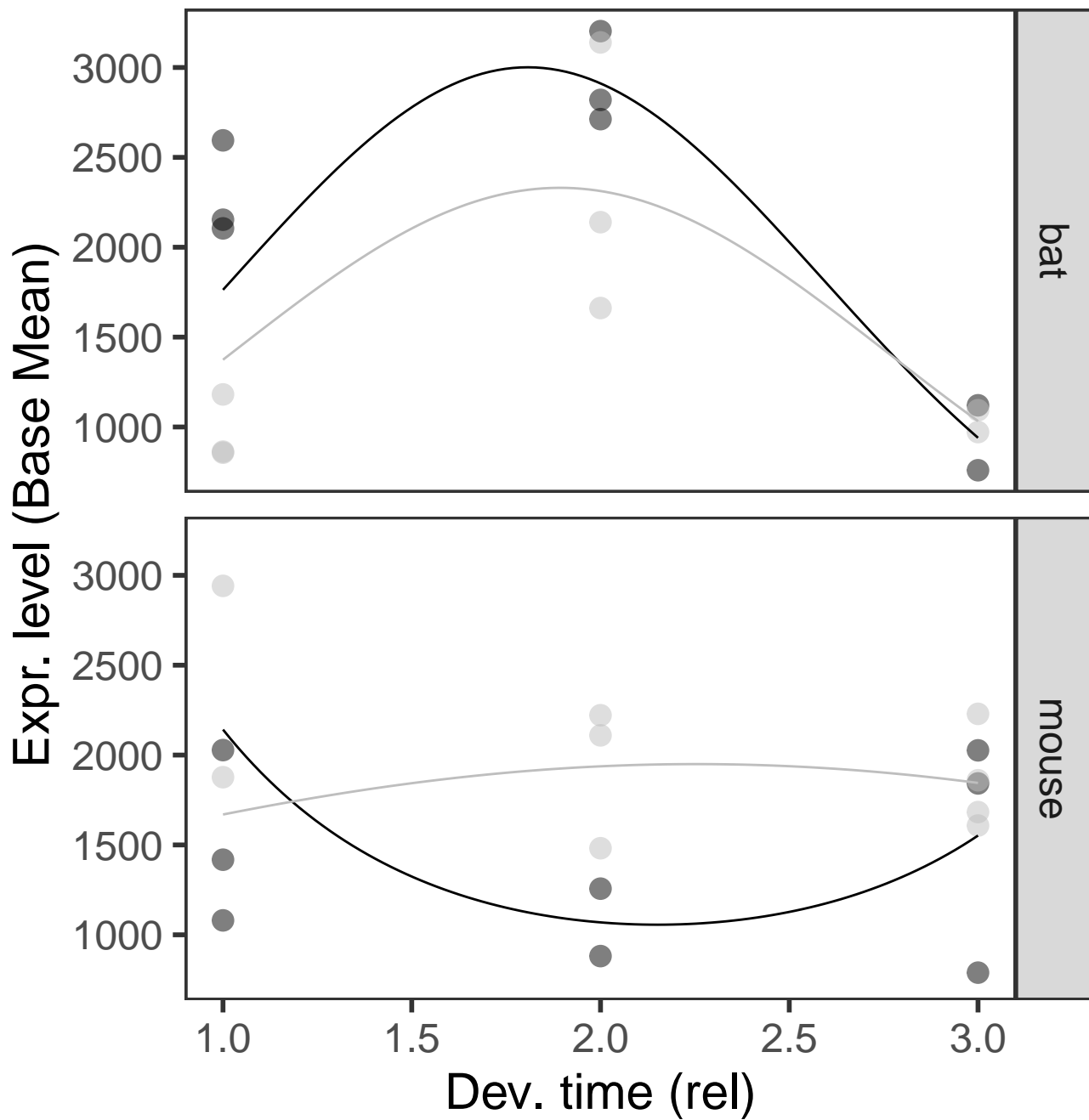

Limb

- fore-limb
- hind-limb

Supplement: Supplementary file 6 — Source Data [file 41467_2025_55826_MOESM6_ESM.zip › source data/Code_et_data_for_Fig/Code_and_data_for_fig6/Grem1_fig6_panelBD.pdf]

**Fgf8**

Expr. level (Base Mean)

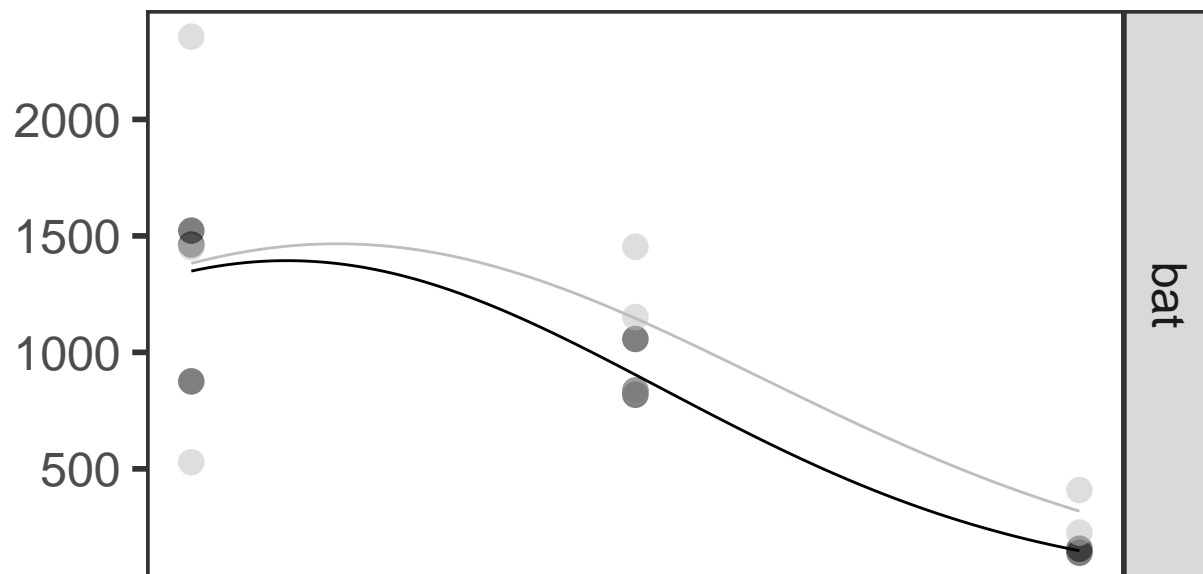

**Limb**

- fore-limb
- hind-limb

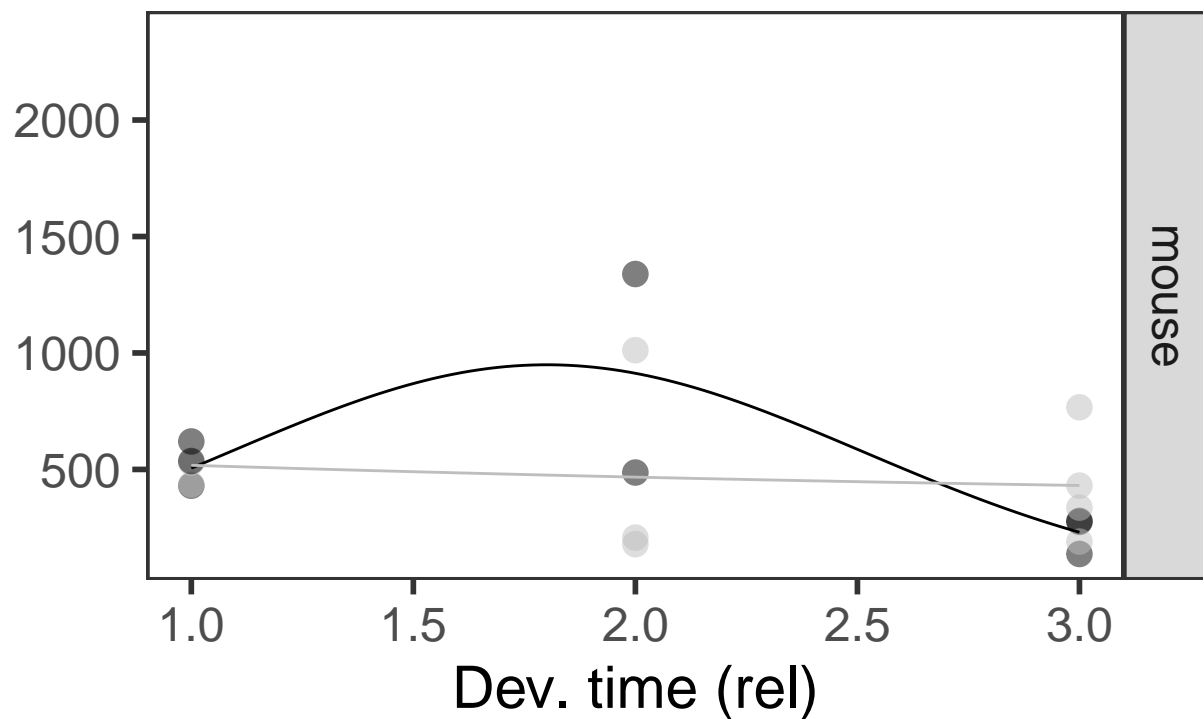

Supplement: Supplementary file 6 — Source Data [file 41467_2025_55826_MOESM6_ESM.zip › source data/Code_et_data_for_Fig/Code_and_data_for_fig6/Fgf8_fig6_panelBD.pdf]

Shh

Expr. level (Base Mean)

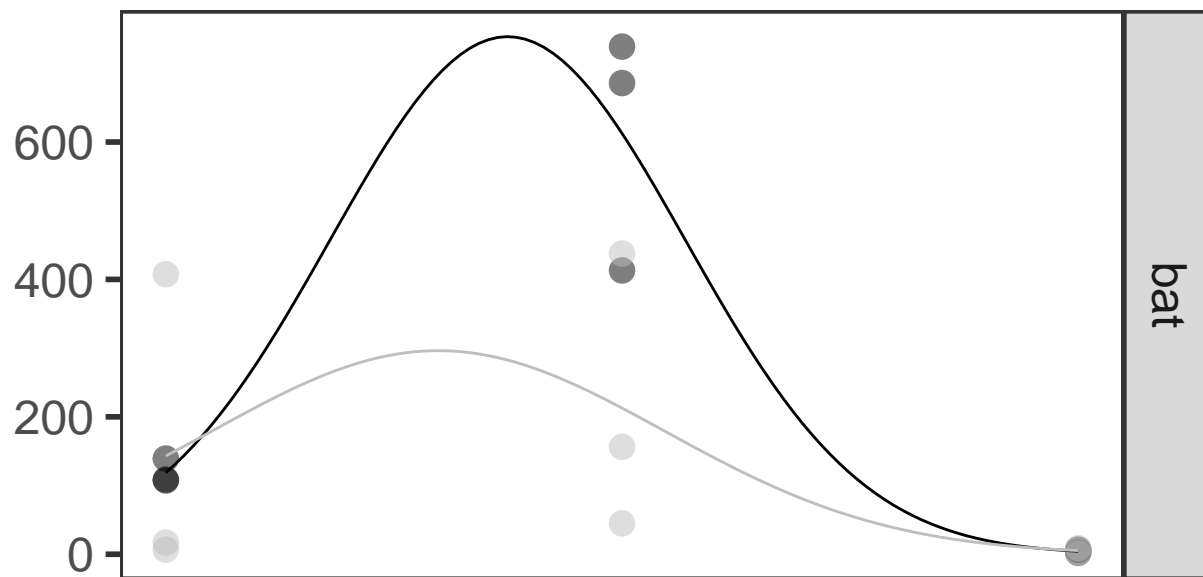

bat

Limb

- fore-limb
- hind-limb

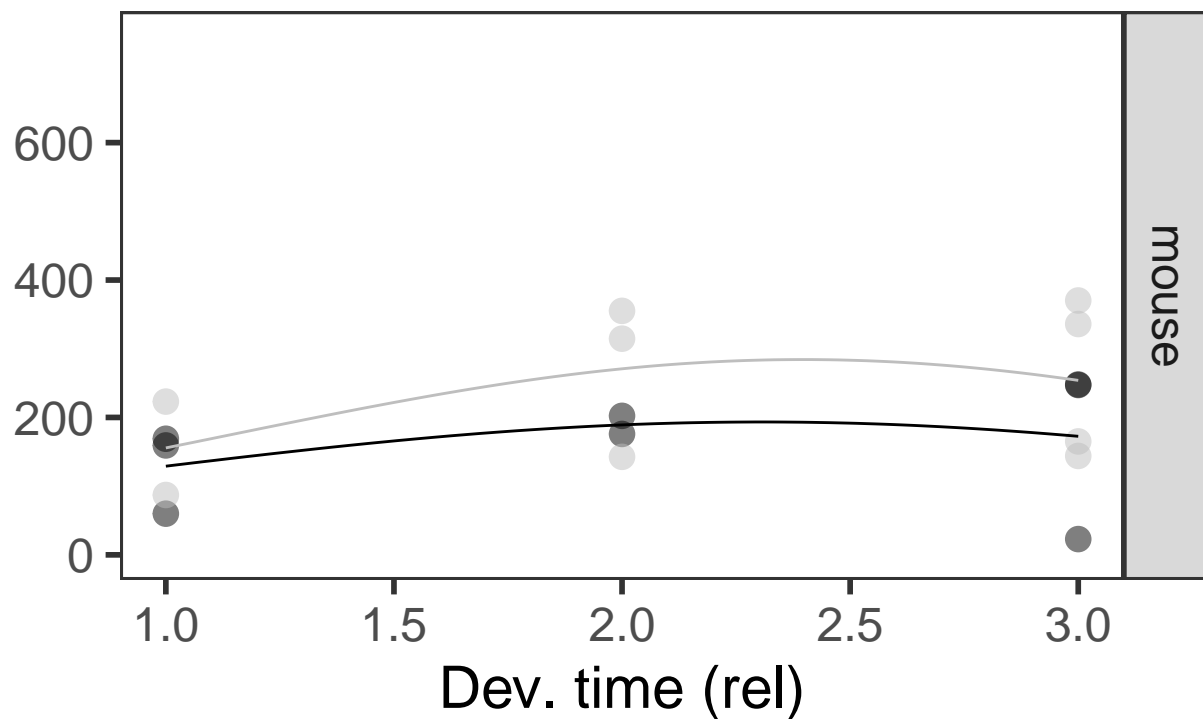

mouse

Supplement: Supplementary file 6 — Source Data [file 41467_2025_55826_MOESM6_ESM.zip › source data/Code_et_data_for_Fig/Code_and_data_for_fig6/fig6_panelB.pdf]

**Fgf8**

Expr. level (Base Mean)

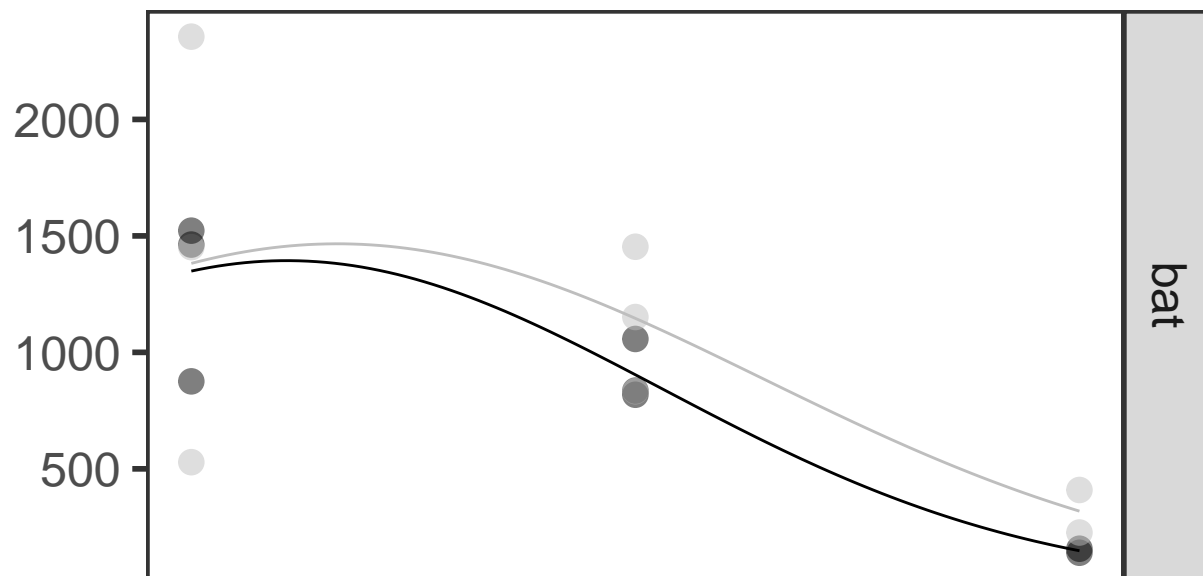

**Limb**

- fore-limb
- hind-limb

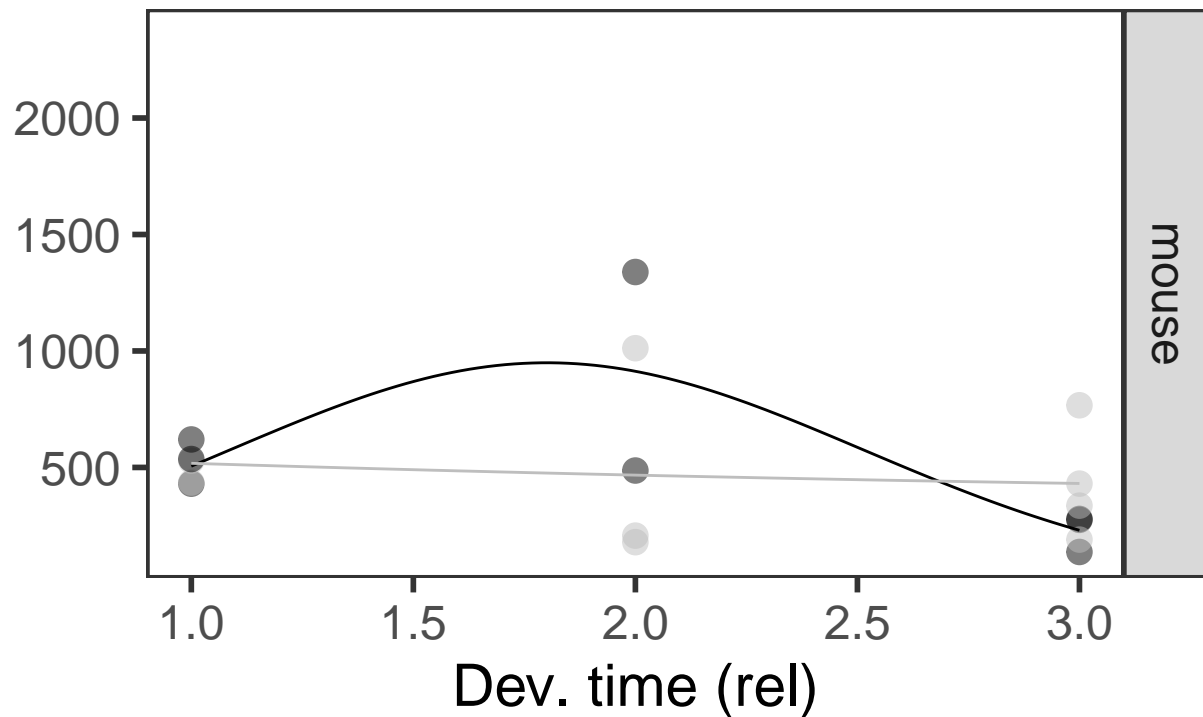

Supplement: Supplementary file 6 — Source Data [file 41467_2025_55826_MOESM6_ESM.zip › source data/Code_et_data_for_Fig/Code_and_data_for_fig6/fig6_panelC.pdf]
